# Iminoboronates are efficient intermediates for selective, rapid and reversible *N*-terminal cysteine functionalisation†
†Electronic supplementary information (ESI) available. See DOI: 10.1039/c6sc01520d


**DOI:** 10.1039/c6sc01520d

**Published:** 2016-06-16

**Authors:** Hélio Faustino, Maria J. S. A. Silva, Luís F. Veiros, Gonçalo J. L. Bernardes, Pedro M. P. Gois

**Affiliations:** a Research Institute for Medicines (iMed.ULisboa) , Faculty of Pharmacy , Universidade de Lisboa , Lisbon , Portugal . Email: pedrogois@ff.ulisboa.pt; b Centro de Química Estrutural , Instituto Superior Técnico , Universidade de Lisboa , Av. Rovisco Pais 1 , 1049-001 Lisbon , Portugal; c Department of Chemistry , University of Cambridge , Lensfield Road , CB2 1EW , Cambridge , UK; d Instituto de Medicina Molecular , Faculdade de Medicina , Universidade de Lisboa , Avenida Professor Egas Moniz , 1649-028 , Lisboa , Portugal

## Abstract

We show that formyl benzeno boronic acids (2FBBA) selectively react with *N*-terminal cysteines to yield a boronated thiazolidine featuring a B–N bond. The reaction exhibits a very rapid constant rate (2.38 ± 0.23 × 10^2^ M^–1^ s^–1^) under mild aqueous conditions (pH 7.4, 23 °C) and tolerates different amino acids at the position adjacent to the *N*-cysteine. DFT calculations highlighted the diastereoselective nature of this ligation reaction and support the involvement of the proximal boronic acid in the activation of the imine functionality and the stabilisation of the boronated thiazolidine through a chelate effect. The 2FBBA reagent allowed the effective functionalisation of model peptides (C-ovalbumin and a laminin fragment) and the boronated thiazolidine construct was shown to be stable over time, though the reaction was reversible in the presence of benzyl hydroxylamine. The reaction proved to be highly chemoselective, and 2FBBA was used to functionalize the *N*-terminal cysteine of calcitonin in the presence of a potentially competing in-chain thiol group. This exquisite selectivity profile enabled the dual functionalisation of calcitonin and the interactive orthogonal modification of this peptide when 2FBBA was combined with conventional maleimide chemistry. These results highlight the potential of this methodology to construct complex and well-defined bioconjugates.

## Abstract 2

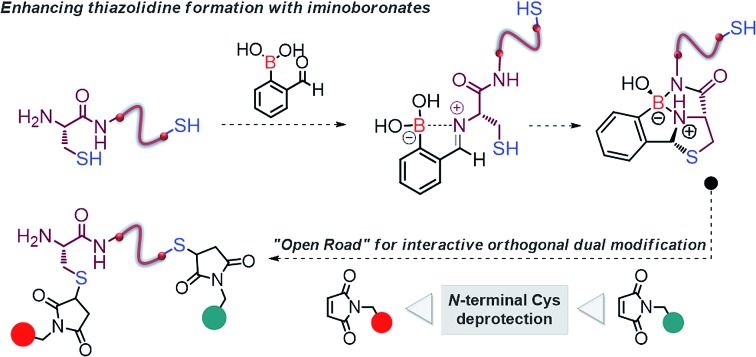
Formyl benzeno boronic acids rapidly and selectively react with *N*-terminal cysteines to yield a reversible boronated thiazolidine that may be used in the interactive orthogonal modification of peptides.

## Introduction

In recent years, the chemical functionalisation of peptides and proteins targeting the side chains of naturally occurring amino acids has developed into a powerful strategy for studying fundamental biological processes, as well as for constructing therapeutic drugs and functional hybrid materials without the need for more specialized techniques.^1^ Among the 20 canonic amino acids, cysteine (Cys) is a residue with low abundance (<2%) that exhibits a highly reactive sulfhydryl side chain.^1^,^2^ For these reasons, native or engineered Cys on the protein surface has emerged as a preferred “hot-spot” for the site-selective modification of proteins and the construction of well-defined bioconjugates.^1^–^3^ The functionalisation of this residue has been effectively achieved by promoting the thiol group elimination to generate dehydroalanine, *via* the thiol–ene reaction or by exploring the nucleophilicity of thiol in the presence of electrophiles.^1^–^3^ These chemistries specifically target the sulfhydryl side chain and are mostly unselective when similar reactive thiol groups are present at the surface of the biomolecule.^2^ Therefore, the construction of more complex and well-defined bioconjugates, without resorting to mutant proteins,^4^ the genetic encoding of specific amino acid sequences^5^ or using multivalent reactive handles,3b–d is dependent on the discovery of new chemical methods that can orthogonally functionalize Cys residues.^6^

**Scheme 1:**
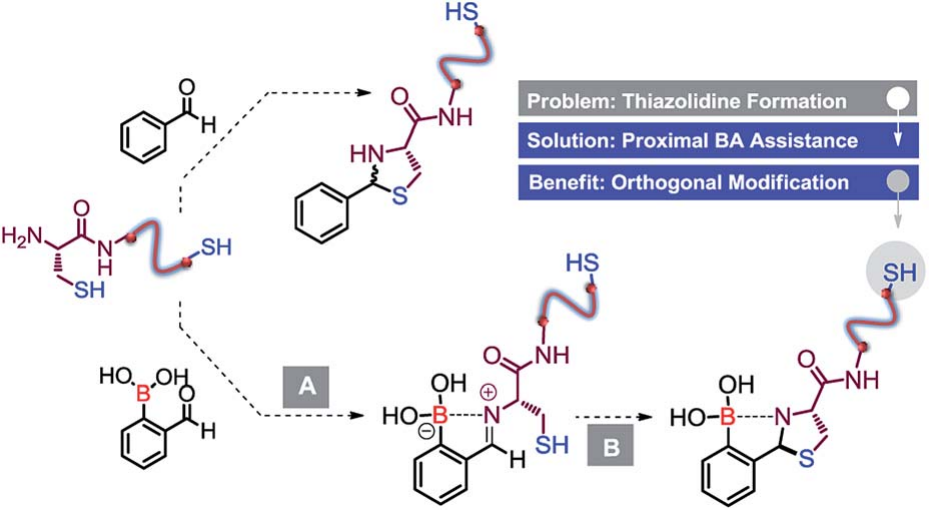
*N*-Terminal cysteine modification *via* thiazolidine and proposed proximal boronic acid assisted thiazolidine formation.

One of the most attractive ways to differentiate sulfhydryl side chains is to chemically target the *N*-terminal Cys residue with thioesters (native chemical ligation),^7^ aromatic cyanides^8^ or aldehydes,^9^ as these reagents selectively modify the 1,2-aminothiol in the presence of competing in-chain or C-terminal sulfhydryl side chains. Among these, thiazolidine formation is a potentially powerful strategy to modify this residue,^9^ because it uses widely available, stable and structurally diverse aldehydes as a condensation partner. Taking this into consideration, and with the exception of a few examples,^9^,^10^ thiazolidine formation reactions have not emerged as useful site-selective bioconjugation tools because the conditions required for the condensation are typically incompatible with a wide range of biomolecules. Namely, this reaction proceeds at an acidic pH of 4–5, requires long reaction times (up to several days) and multiple equivalents of reactants, and if successful it generates the thiazolidine as a mixture of diastereoisomers (Scheme 1).^1^,^9^,^10^

## Results and discussion

Recognizing the potential of the thiazolidine method for orthogonal Cys bioconjugation, we initiated a study to improve the profile of this reaction. Recently we showed that 2-acetyl benzeno boronic acids (2ABBA) reversibly functionalize protein exposed lysine residues *via* the formation of iminoboronates.^11^ The boronic acid *ortho* to the carbonyl is key for the success of this bioconjugation as it promotes the imine formation and stabilizes the linkage by forming a N–B dative bond.^11^ This proximal boronic acid assistance was recently used by others to enhance the formation of oximes, hydrazones and benzothiazoles.^12^ Based on this, we reasoned that the condensation reaction of aldehydes with *N*-terminal Cys residues could also be significantly improved by generating a transient iminoboronate *en route* to the cyclisation (Scheme 1).

While submitting this manuscript, Gao *et al.* reported the labelling of *N*-terminal cysteines with 2FBBA. Whereas the disclosed data is consistent with our results, herein we considerably expand the scope of this reaction, exploring the orthogonality and reversibility of the system in the selective dual labelling of peptides. In addition, we present a detailed study based on DFT calculations that highlight the invaluable role of the proximal boronic acid in the reaction mechanism.12*d*

**Scheme 2:**
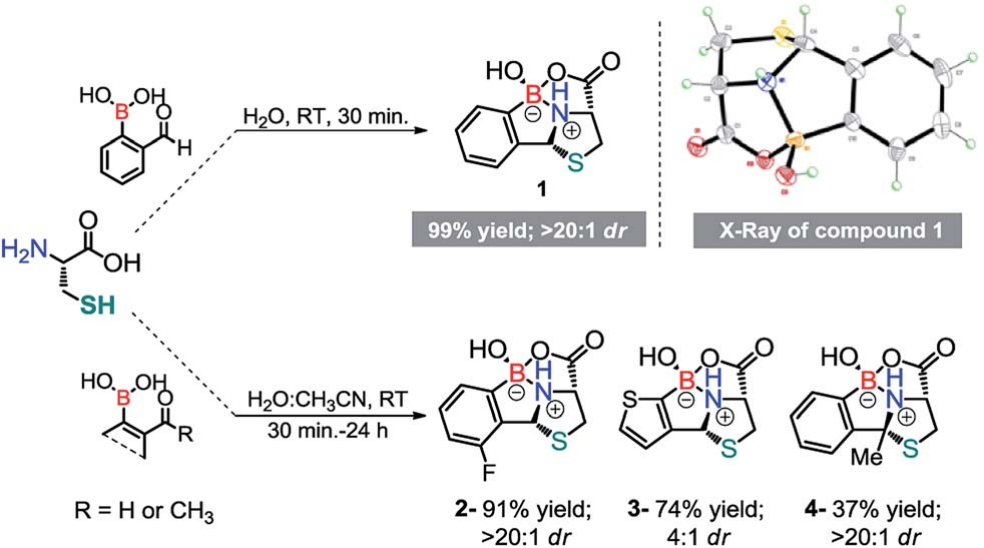
Reaction of Cys with different boronic acid scaffolds.

To test our idea, we started by performing a reaction between equimolar amounts of 2-formyl benzeno boronic acid (2FBBA) and Cys in water. To our surprise, the reaction proceeded smoothly at room temperature, and after 30 min compound **1** precipitated from the reaction mixture. The desired product was collected by simple decantation with a 99% yield, high purity and as almost a single diastereoisomer (>20 : 1 dr). The unanticipated geometry of this tricyclic fused heterocycle, featuring an sp^3^ boron centre involved in a N–B bond, was further elucidated based on analysis of the X-ray structure depicted in Scheme 2. Motivated by the efficiency of this assemblage, we tested the possibility of using different boronic acid scaffolds, as shown in Scheme 2. Fluorination of the aromatic ring had no impact on the reaction (**2**), while the condensation using 3-formyl-2-thienylboronic acid was significantly less diastereoselective (**3**). Surprisingly, 2ABBA also reacted under these rather mild conditions, yielding heterocycle **4** with a fully substituted quaternary carbon centre in 37% yield.

**Scheme 3:**
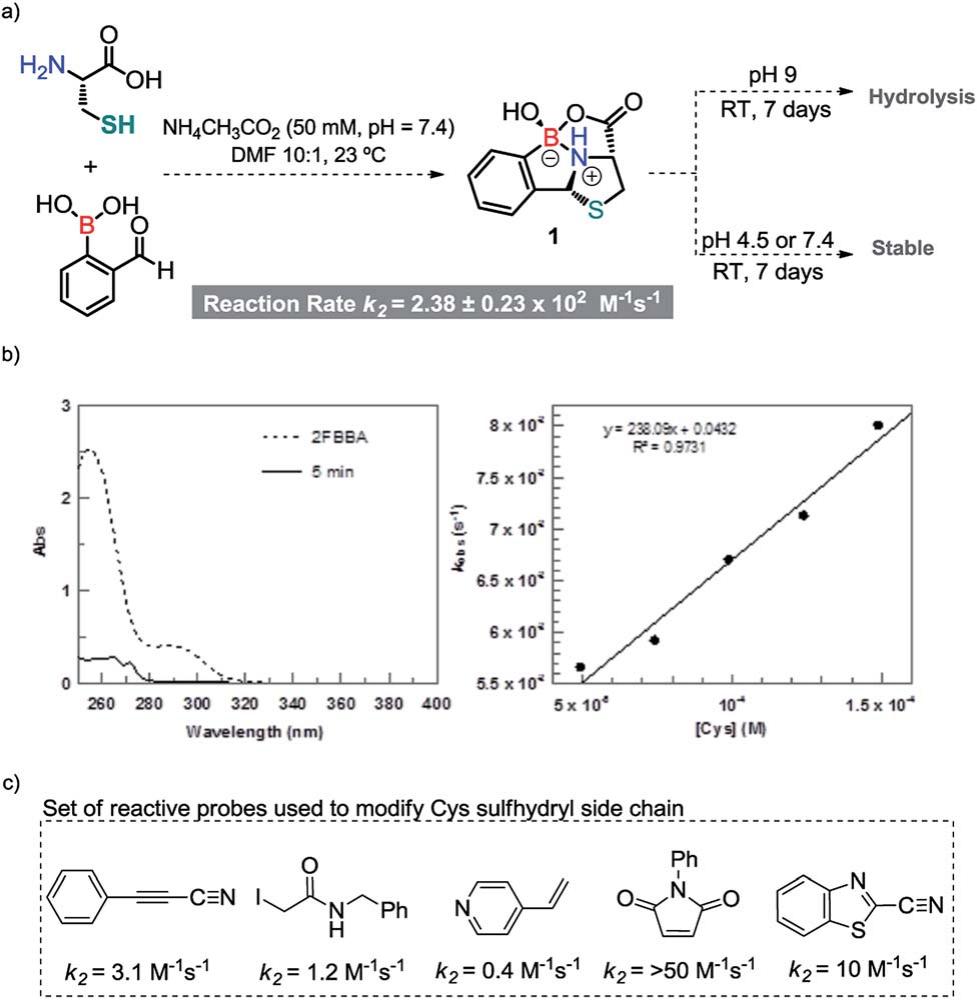
(a) Thiazolidine formation and stability at various pH values. (b) Left – UV-vis absorption of 2-FBBA and the reaction mixture at 5 min; right – *k*_obs_ values plotted against the concentration of cysteine to yield the second order rate constant (*k*_2_, M^–1^ s^–1^) from the slope of the line. (c) Probes for the modification of cysteine.

Next, we studied the impact of the proximal boronic acid on the condensation rate. To assess this, we monitored the reaction between 2FBBA (200 μM) and Cys (1.5 equiv.) in acetate buffer (50 mM) : DMF (10 : 1) at pH 7.4 using UV-Vis. As shown in Scheme 3a, this is a very fast reaction in which the aldehyde is readily consumed to yield the desired product in less than 5 min. In contrast, the reaction of benzaldehyde with l-Cys failed to deliver the thiazolidine product under the same reaction conditions, even when left for longer periods of time (see Fig. S1 and S2, ESI†).

Interestingly, the free energy balances calculated for the two reactions by DFT^13^ corroborate these results, with the reaction of 2FBBA with Cys being more favourable (by 17 kcal mol^–1^) than the benzaldehyde one (see Fig. S29, ESI†). Product **1** is stabilized by the chelate effect on the boron atom, providing an extra thermodynamic drive to the reaction. These results demonstrated the importance of the neighbouring boronic acid for the success of the condensation reaction; hence, the reaction rate was determined under pseudo first order conditions. As shown in Scheme 3b, the condensation reaction was confirmed as a very fast process exhibiting a rate constant of 2.38 ± 0.23 × 10^2^ M^–1^ s^–1^, which compares well with standard reagents used for bioconjugation of Cys residues (Scheme 3c). In particular, 2FBBA is 20 times faster than cyanobenzothiazole (10 M^–1^ s^–1^), which is commonly used for bioconjugation of terminal 1,2-aminothiols.^14^

Once we had established the formation of this construct, we decided to also evaluate the stability of the heterocycle in ammonium acetate buffer. Despite exhibiting a rather complex structure, **1** proved to be stable (over 7 days) under acidic or neutral conditions, and only at pH 9 did **1** slowly hydrolyze into its individual components (see ESI, Fig. S7†).

**Scheme 4:**
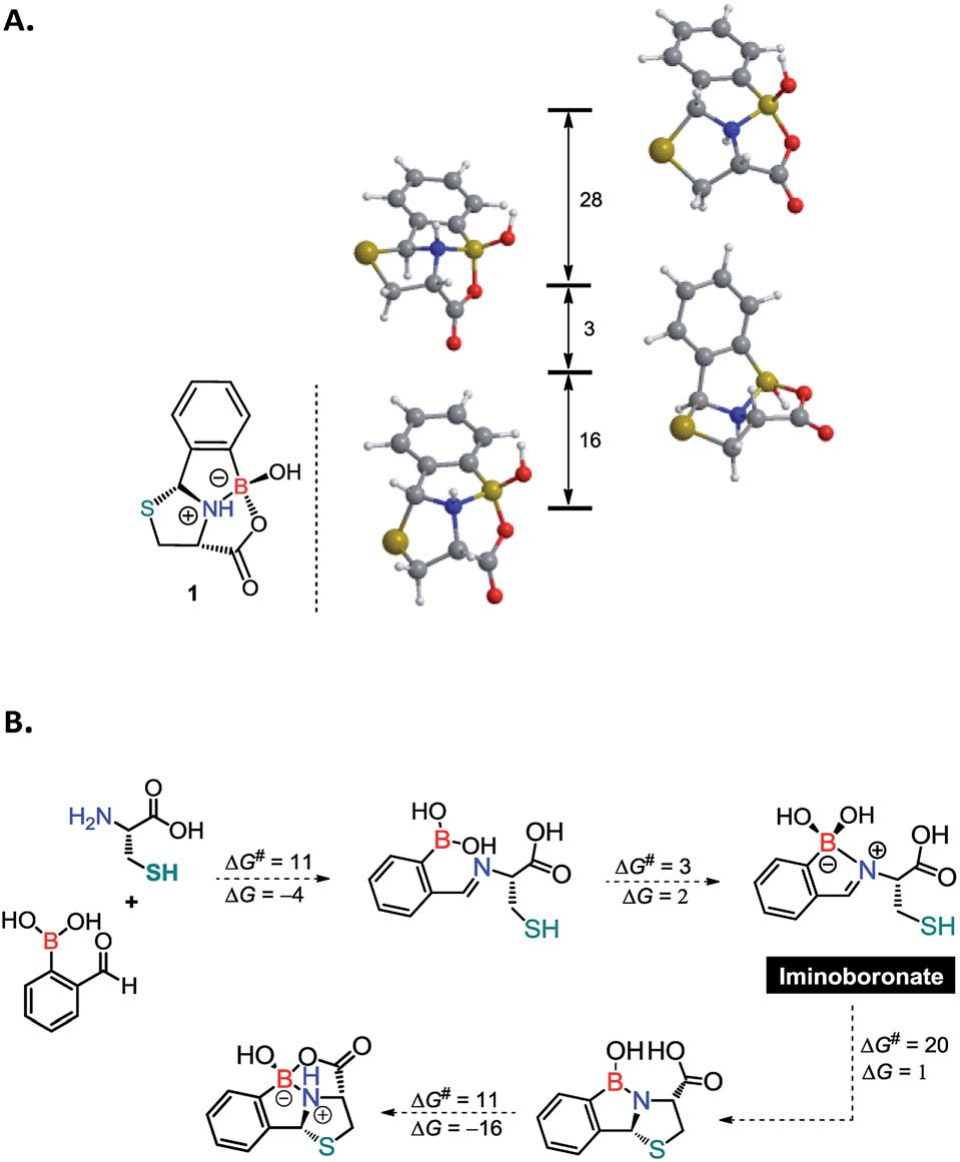
(A) Difference in stability between the four more stable diastereomers of product **1**. (B) Schematic representation of the most important intermediates in the mechanism of formation of **1** from Cys and 2FBBA. Free energy values in kcal mol^–1^.

As previously mentioned, the thiazolidine condensation often yields conjugates as an inseparable mixture of diastereoisomers. However, the condensation between 2FBBA and Cys afforded the construct **1** in a highly diastereoselective manner. To elucidate this aspect of the reaction, the four more stable stereoisomers of product **1**, each with a different configuration on the N-atom or on the C2-atom of the thiazolidine ring, were studied using DFT calculations.^15^ The results obtained are depicted in Scheme 4A and indicate a clear preference for the diastereoisomer observed experimentally. This corresponds to the less strained structure, *i.e.*, the one with a better balance of the ring tension due to the presence of three fused 5-membered rings around the N-atom. The results in Scheme 4 show that the product obtained corresponds to the most stable diastereoisomer and indicate that the reaction is thermodynamically controlled.

Motivated by these results, we next used DFT calculations to elucidate the role of the proximal boronic acid in the reaction mechanism. A full account of the mechanism is given in the ESI† including all calculated energy profiles (see ESI, Fig. S30 and S31†) and detailed representations of the path (see ESI, Schemes S1 and S2†). The more important features of the proposed mechanism are presented in Scheme 4B. The mechanism calculated for the formation of **1** from 2FBBA and Cys comprises four main parts: the first corresponds to the condensation reaction between the amine group of *N*-Cys and the aldehyde of the boronic acid to produce an imine, involving the two initial reactants. This first section of the mechanism has a rather accessible barrier of Δ*G*^‡^ = 11 kcal mol^–1^ and, overall, is exergonic by 4 kcal mol^–1^, as expected for a reversible reaction. In the second part of the mechanism the formation of a bond between the imine N-atom and the boron occurs, resulting in a 5-membered B-chelate ring. In this intermediate there is a tetravalent, formally negative B-atom and an iminium group. This is a very facile step, almost barrierless (Δ*G*^‡^ = 3 kcal mol^–1^) and only slightly endergonic (Δ*G* = 2 kcal mol^–1^). The activation of the C-atom of the iminium group, accomplished through the coordination of the nitrogen to the boron atom, makes possible the thiol attack and the resulting S–C in the next step of the mechanism. Formation of the new S–C bond occurs simultaneously with proton transfer from the SH group to one of the OH groups attached to the B-atom and subsequent loss of the resulting water molecule. Thus, in the following intermediate there is a second 5-membered ring fused to the first one by the C–N bond and a trivalent (neutral) B-atom stabilized by two coordinating atoms, the O-atom of the OH group and the N-atom of the amine. This section of the mechanism has the highest barrier of the entire path (Δ*G*^‡^ = 20 kcal mol^–1^) but it is essentially thermoneutral (Δ*G* = 1 kcal mol^–1^). The last part of the mechanism corresponds to attack on the B-atom by the carboxylic group with formation of a new B–O bond and concurrent proton transfer to the amine N-atom. Thus, in the product **1** there is a third 5-membered ring fused with the previous two, which is also another B-chelating ring. The barrier for this last section is moderate, Δ*G*^‡^ = 11 kcal mol^–1^, and the corresponding free energy balance is clearly exergonic (Δ*G* = –16 kcal mol^–1^).

The overall path indicates a feasible reaction with a barrier of Δ*G*^‡^ = 21 kcal mol^–1^ and a clearly favourable free energy balance of Δ*G* = –19 kcal mol^–1^, indicating the stability of the product as the main driving force for the reaction. Importantly, the B-atom has a two-fold role in the reaction mechanism. On the one hand, it provides activation of the imine group by means of N–B coordination, promoting the formation of the S–C bond, and on the other, it affords additional stabilisation of the final product through multiple boron-coordination and the corresponding chelate effect.

**Scheme 5:**
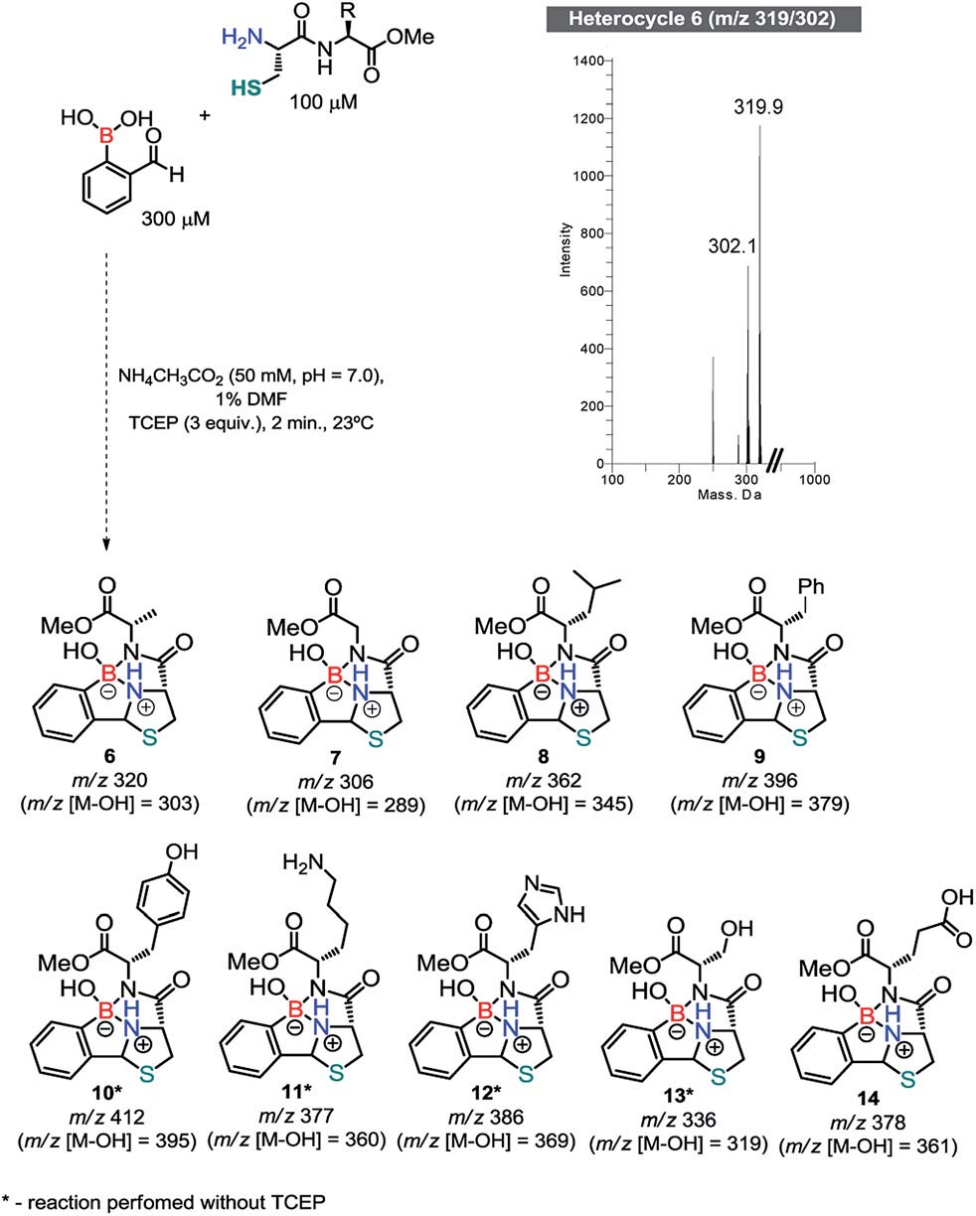
Conjugation of 2FBBA with a variety of *N*-terminal cysteine dipeptides.

Once we had established the key features of this condensation, we evaluated the reaction between 2FBBA and model dipeptides featuring a *N*-terminal Cys. We started by reacting Cys-Ala-OMe (100 μM) with 3 equiv. of 2FBBA in ammonium acetate buffer (20 mM, pH 7.0) at 23 °C. Gratifyingly, the more complex structure of **5** was not detrimental for the reaction and the tricyclic heterocycle **6** (*m*/*z* 319/302) was readily assembled under these conditions in less than 5 min. Based on this result, other peptides were prepared and reacted with 2FBBA. As shown in Scheme 5, all peptides reacted efficiently under these conditions and the assemblage was complete within 5 min at 23 °C. Peptides constructed with amino acids such as glutamate (Glu), tyrosine (Tyr), serine (Ser) and Lys, presenting functionalities that could in principle disturb the assemblage of the heterocyclic framework, were relatively innocent in this process, demonstrating the robustness and generality of this bioconjugation method (see ESI, Fig. S8–16†).

**Scheme 6:**
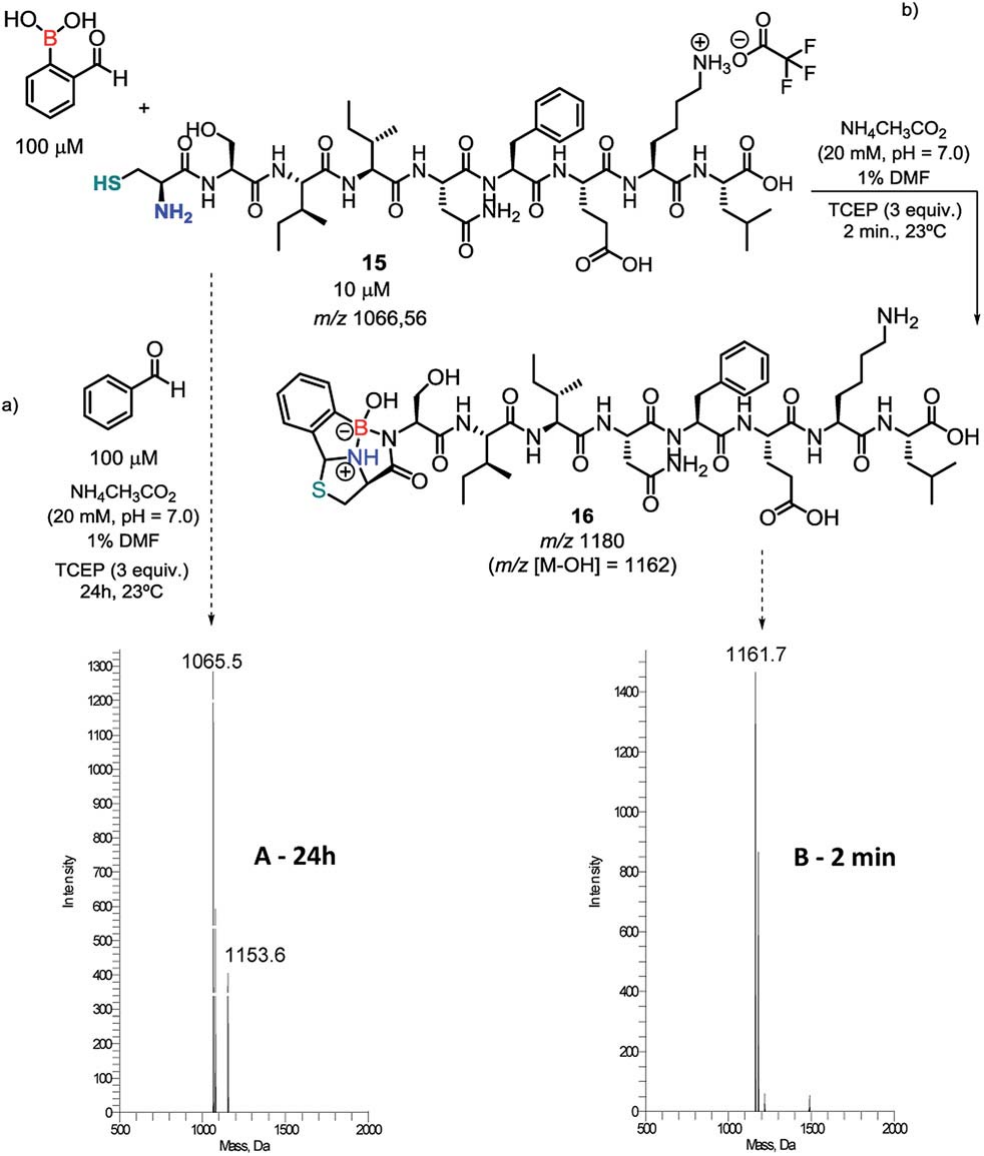
(a) Reaction of C-ovalbumin with benzaldehyde; (b) reaction of C-ovalbumin with 2FBBA.

Encouraged by these results, an ovalbumin derived peptide exhibiting an *N*-terminal Cys residue was constructed and submitted to conjugation with 10 equiv. of 2FBBA. As shown in Scheme 6, the expected construct (*m*/*z* 1180/1162) was immediately formed at pH 7 and remained stable in solution for up to 24 h in this medium (see ESI, Fig. S17†). Similar results were obtained when using only 3 equiv. of the 2FBBA reagent (Scheme 6). Interestingly, the presence of a Lys residue did not affect the assemblage of **16**, probably due to higher reversibility of the iminoboronate formed with the lysine ε-amino group.

To further demonstrate the importance of the proximal boronic acid for the conjugation, benzaldehyde was reacted with peptide **15** and the reaction was monitored by ESI-MS. As shown in Scheme 6a, the thiazolidine (*m*/*z* 1154) was only detected in the reaction mixture after 24 h, corroborating that the standard thiazolidine condensation is not a useful bioconjugation tool under these conditions.

**Scheme 7:**
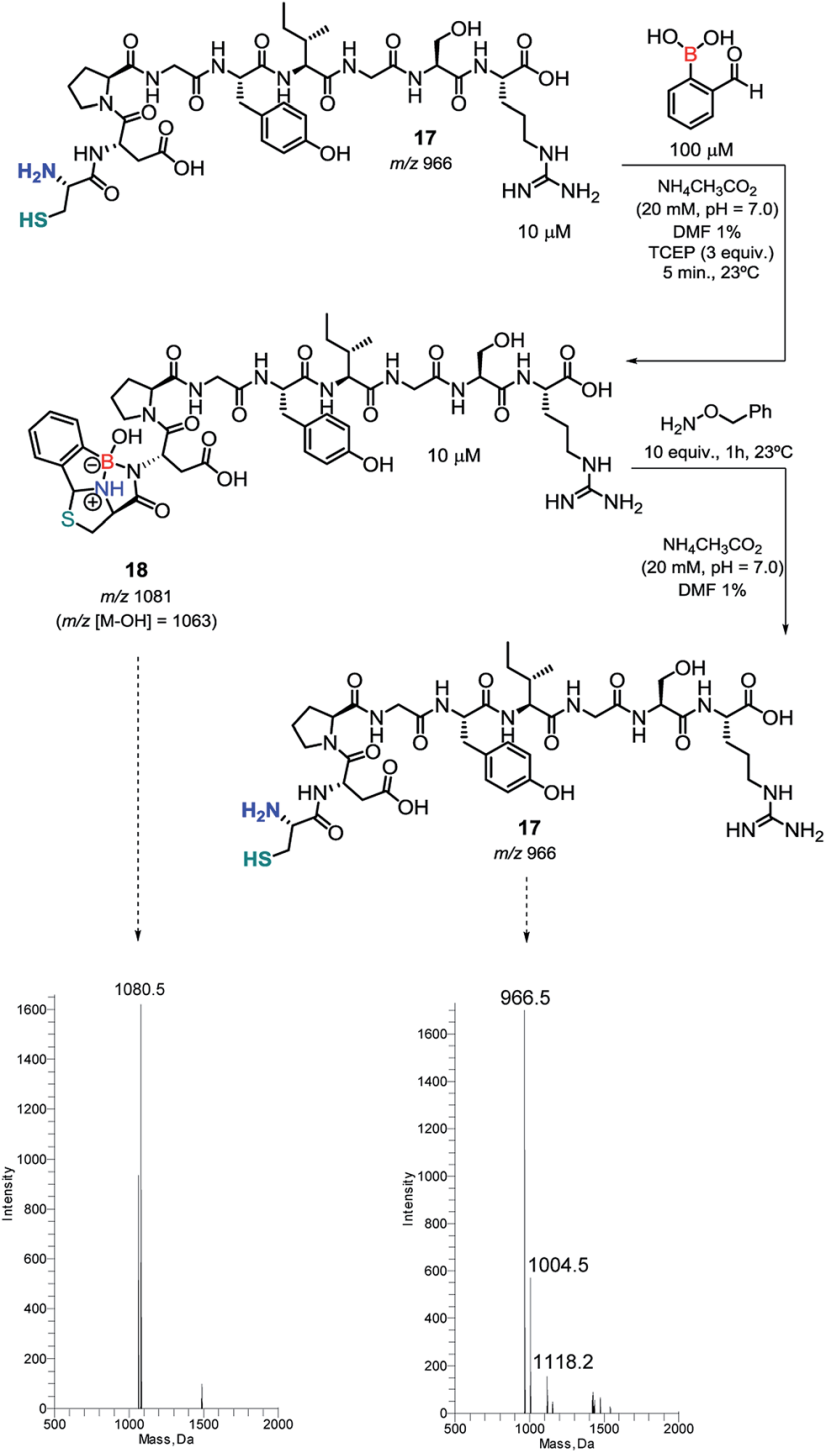
Condensation of 2FBBA with a laminin fragment and its reversibility promoted by benzyl hydroxylamine.

Then, we tested the condensation of 2FBBA with a laminin fragment that exhibits several residues that may interfere directly with the boronic acid function. As shown in Scheme 7, despite the presence of a Glu adjacent to the *N*-terminal or in chain Tyr and Ser residues, the assemblage with 2FBBA proceeded smoothly to yield the expected construct (*m*/*z* 1081/1063) under the optimized reaction conditions.

Thiazolidine is known to be a reversible linkage in the presence of hydroxylamine derivatives. Therefore, and to understand if this key feature of thiazolidines is retained in our newly formed constructs, we decided to treat compound **18** with 10 equiv. of benzyl hydroxylamine. As expected, the construct was fully hydrolyzed to the corresponding parent peptide.

**Scheme 8:**
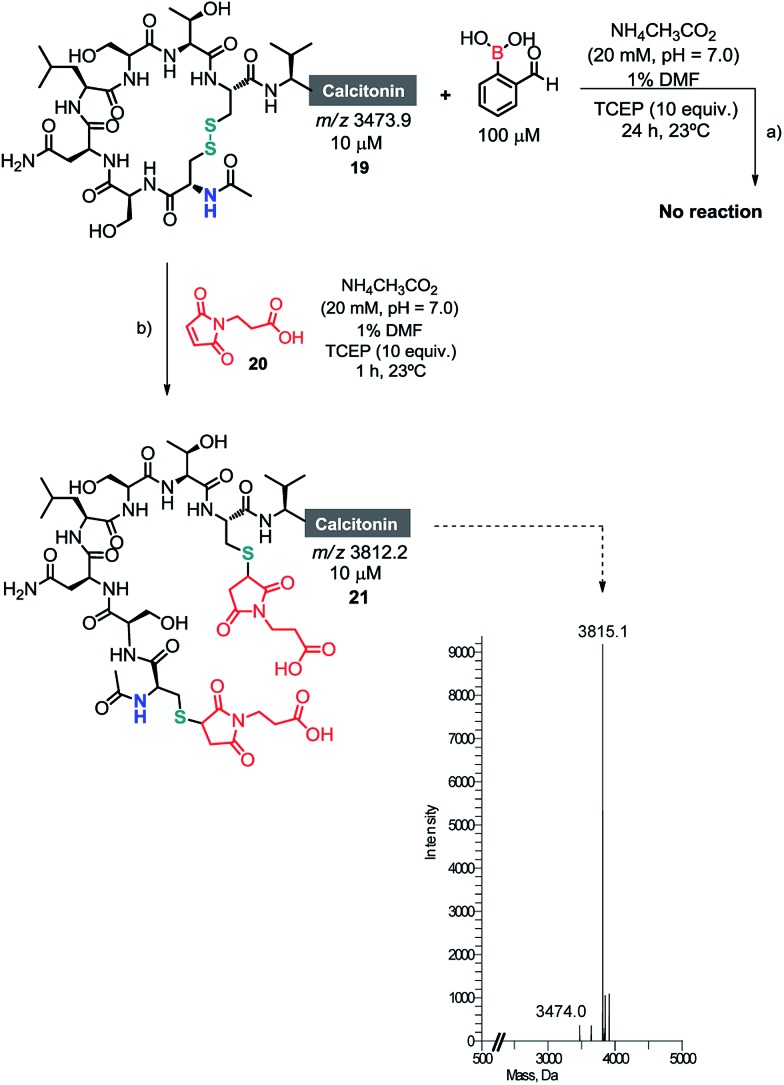
(a) Acetyl salmon failed to give any product in the presence of 2FBBA; (b) alkylation of the exposed thiol groups with maleimide **20**.

**Scheme 9:**
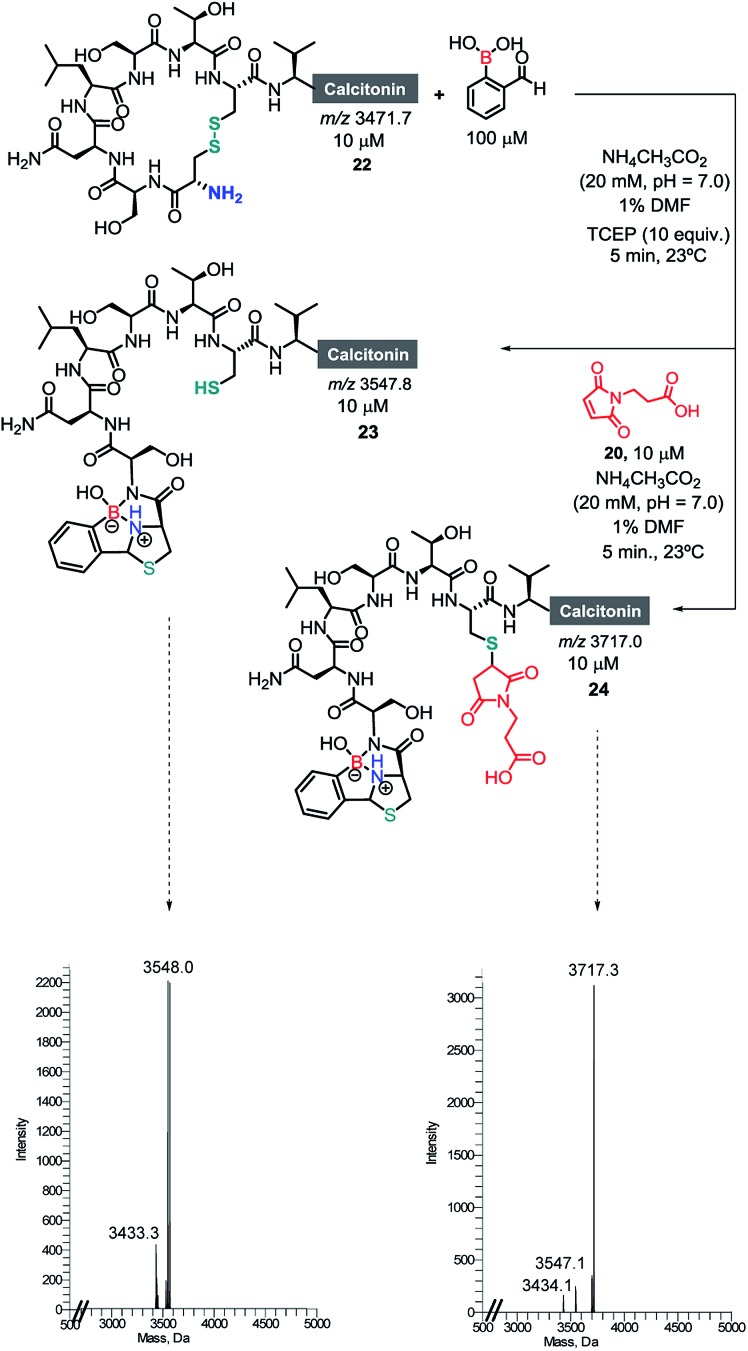
Selective modification of *N*-terminal and in-chain cysteines with 2FBBA and maleimide **20**, respectively.

Once we had established the assemblage of Cys *N*-terminal peptides with 2FBBA, the reaction was evaluated with peptides featuring in-chain Cys residues. To study this, the therapeutic peptide *N*-acetylated calcitonin was treated with TCEP to reduce the disulphide bridge, and then reacted first with a model maleimide and subsequently with 2FBBA. As expected, the exposed thiol groups were efficiently alkylated with maleimide **20** (Scheme 8). In contrast, under the same reducing conditions, the peptide failed to react with the boronic acid scaffold, suggesting that acetylation at the nitrogen precludes the generation of the construct with 2FBBA and supports the formation of the iminoboronate *en route* to the cyclisation. To further explore this observation, the non-acetylated calcitonin **22** was submitted to conjugation with 2FBBA in the presence of TCEP (Scheme 9). Gratifyingly, under these conditions, the reaction proceeded efficiently affording the conjugate **23** in less than 5 min. Next, we assumed that the formation of **23** occurred without reaction of the in-chain thiol group, making it available for one additional round of functionalisation. Hence, conjugate **23** was treated with maleimide **20**, and this simple protocol yielded the conjugate **24**, orthogonally modified at both Cys residues (Scheme 9).

**Scheme 10:**
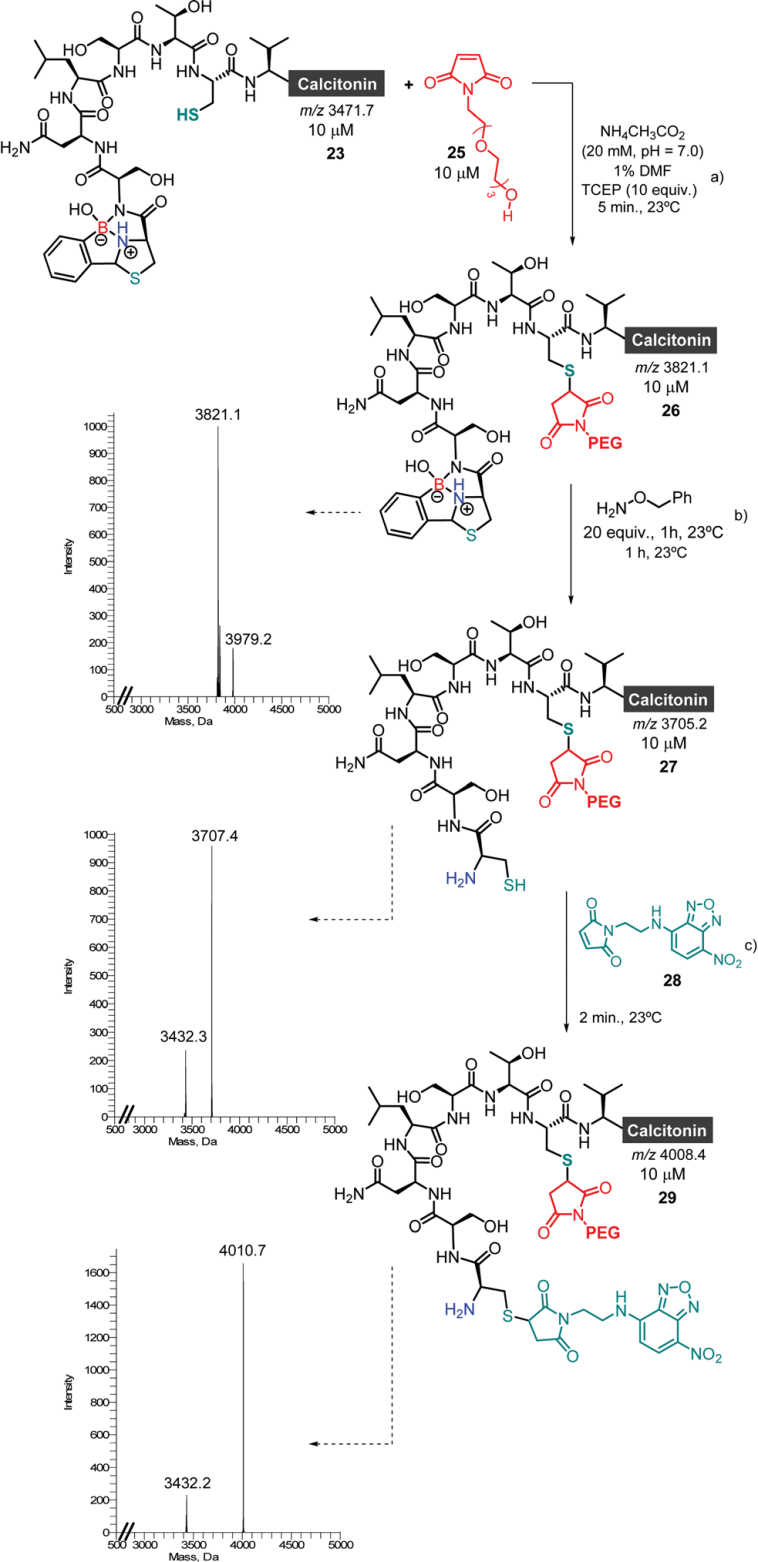
(a) Selective modification of *N*-terminal and in-chain Cys with 2FBBA and maleimide **24**, respectively; (b) removal of 2FBBA by addition of benzyl hydroxylamine; (c) selective modification of *N*-terminal Cys with maleimide **28**.

Finally, the ability to revert the functionalisation carried out with 2FBBA using benzyl hydroxylamine (Scheme 7) offers the possibility to use this scaffold as a protecting group for the *N*-terminal Cys and thus to engage in an interactive orthogonal dual modification. To test this, the conjugate **23** was functionalized with a PEG-maleimide, and subsequently treated using 20 equiv. of benzyl hydroxylamine (Scheme 9). Then, the boron construct was effectively removed, exposing the *N*-terminal sulfhydryl side chain that could now be simply modified using maleimide **28** (Scheme 10).

## Conclusions

In this study we showed that 2FBBA effectively reacts with the 1,2-aminothiol function of *N*-terminal Cys to generate a boronated thiazolidine featuring a B–N bond. The reaction is very rapid (2.38 ± 0.23 × 10^2^ M^–1^ s^–1^) and proceeds under mild conditions (pH 7.4, 23 °C) at a near stoichiometric ratio of reagents. DFT calculations performed on this system showed that the proximal B-atom has a dual role. On the one hand, it provides activation of the imine group by means of N–B coordination, promoting the formation of the S–C bond, and on the other, it affords additional stabilisation of the final product through multiple boron-coordination and the corresponding chelate effect. Regarding the reaction scope, the functionalisation proceeds efficiently with structurally diverse *N*-terminal Cys dipeptides (*e.g.*, Gly, Tyr, Ser and Lys), bearing functionalities that could in principle disturb the assemblage of the heterocyclic framework. Similarly, model peptides including C-ovalbumin and a laminin fragment reacted effectively with 2FBBA. The boronated thiazolidine constructs were shown to be stable in acidic conditions, at neutral pH and in slightly basic conditions (<pH 9), though the reaction was fully reversible upon the addition of benzyl hydroxylamine. The reaction was shown to be highly selective and 2FBBA was used to functionalize the *N*-terminal Cys of calcitonin in the presence of an in-chain thiol group. This selectivity profile was further explored in a dual functionalisation of calcitonin with a boronated thiazolidine/PEG-maleimide and in an interactive installation of two different maleimides onto this peptide. The enclosed results showcase 2FBBA reagents as a powerful tool to selectively functionalize biomolecules and unravel a new strategy for the orthogonal construction of more complex and well-defined bioconjugates.

## Supplementary Material

Supplementary information
