# Correction: Iminoboronates are efficient intermediates for selective, rapid and reversible *N*-terminal cysteine functionalisation

**DOI:** 10.1039/c6sc90045c

**Published:** 2016-07-20

**Authors:** Hélio Faustino, Maria J. S. A. Silva, Luís F. Veiros, Gonçalo J. L. Bernardes, Pedro M. P. Gois

**Affiliations:** a Research Institute for Medicines (iMed.ULisboa) , Faculty of Pharmacy , Universidade de Lisboa , Lisbon , Portugal . Email: pedrogois@ff.ulisboa.pt; b Centro de Química Estrutural , Instituto Superior Técnico , Universidade de Lisboa , Av. Rovisco Pais 1 , 1049-001 Lisbon , Portugal; c Department of Chemistry , University of Cambridge , Lensfield Road , CB2 1EW , Cambridge , UK; d Instituto de Medicina Molecular , Faculdade de Medicina , Universidade de Lisboa , Avenida Professor Egas Moniz , 1649-028 , Lisboa , Portugal

## Abstract

Correction for ‘Iminoboronates are efficient intermediates for selective, rapid and reversible *N*-terminal cysteine functionalisation’ by Hélio Faustino *et al.*, *Chem. Sci.*, 2016, **7**, 5052–5058.



## 

The authors regret that in the original article there are two instances of incorrect *m*/*z* values.

In Scheme 9, the *m*/*z* value of the starting material, calcitonin **22**, is herein corrected from 3471.7 to 3431.9 and in Scheme 10, the *m*/*z* value of the starting material **23** is herein corrected from 3471.7 to 3547.8.

The Royal Society of Chemistry apologises for these errors and any consequent inconvenience to authors and readers.

