# Supplementary material for: Iminoboronates are efficient intermediates for selective, rapid and reversible N-terminal cysteine functionalisation
Source: Chem Sci. 2016 Jun 16;7(8):5052–8. doi: 10.1039/c6sc01520d (PMC6018717; doi:10.1039/c6sc01520d)
Supplement: Supplementary file 1 [file SC-007-C6SC01520D-s001.pdf]

Electronic Supplementary Material (ESI) for Chemical Science.
This journal is © The Royal Society of Chemistry 2016

Electronic Supplementary Information

For:

Iminoboronates are Efficient Intermediates for Selective, Rapid and Reversible N-Terminal Cysteine Functionalisation

Hélio Faustino,[a] Maria J. S. A. Silva,[a] Luís F. Veiros,[b] Gonçalo J. L. Bernardes,[c] Pedro M. P. Gois*[a]

[a]Research Institute for Medicines (iMed.ULisboa), Faculty of Pharmacy, Universidade de Lisboa, Lisbon, Portugal.
Email: pedrogois@ff.ulisboa.pt
[b]Centro de Química Estrutural, Instituto Superior Técnico, Universidade de Lisboa, Av. Rovisco Pais 1, 1049-001 Lisbon, Portugal.
[c]Department of Chemistry, University of Cambridge, Lensfield Road, CN2 1EW, Cambridge.
Instituto de Medicina Molecular, Faculdade de Medicina, Universidade de Lisboa, Avenida Professor Egas Moniz, 1649-028, Lisboa, Portugal.

Index

1. General Remarks 3
2. Chemical Synthesis and Bioconjugation Procedures 4
2.1 Isolation of the products..4
2.2 UV-vis experiments:..7
2.3 Product stability:...10
2.4 Assays with model dipeptides 13
2.5 Assays with peptides 20
3. Computational Studies 32
4. NMR spectra 46

1. General Remarks

NMR spectra were recorded in a Bruker Avance II 300 Ultrashield Plus. All chemical shifts are reported downfield in parts per million (ppm) and coupling constants (J values) are expressed in Hertz (Hz). Low resolution ESI mass spectra were carried on an ion trap mass analyser (Thermo Scientific LCQ Fleet Ion Trap LC/MS) equipped with an electrospray interface. Pro Mass for Xcalibur (Version 2.8) was used as the deconvolution software. High resolution ESI mass spectra was recorded in a LTQ Orbitrap XL mass spectrometer (Thermo Fischer Scientific, Bremen, Germany) controlled by *LTQ Tune Plus 2.5.5* and *Xcalibur 2.1.0*. Elemental analysis was performed in a Flash 2000 CHNS-O analyzer (ThermoScientific, UK). The X-Ray diffraction experiment was recorded by Andrew Bond, at the Chemical Crystallography X-Ray Service of the University of Cambridge, UK. UV-vis spectra were recorded in a UV-visible spectrophotometer Shimadzu UV-1603. All solvents were of analytical reagent grade and were purchased from Alfa Aesar or Sigma-Aldrich. Calcitonin salmon, L-cysteine, 2-Formylphenylboronic acid and (3-Fluoro-2-formylphenyl)boronic acid were purchased from Alfa Aesar. Calcitonin Acetyl salmon, (3-formyl-2-thienyl)boronic acid, (2-acetylphenyl)boronic, benzaldehyde, TCEP, O-benzylhydroxylamine hydrochloride and 4-Chloro-7-nitro-2,1,3-benzoxadiazole (NBD-Cl) were purchased from Aldrich. Dipeptides were purchased from GeneCust. C-Ovalbumin was purchased from jpt innovative peptide solutions. Laminin fragment was purchased from Carbosynth. Maleimides **20**,[1] and **25**,[2] are known compounds and were prepared according to the described procedures.

[1] Elduque, X.; Sánchez, A.; Sharma, K.; Pedroso, E.; Grandas, A. *Bioconjug. Chem.* **2013**, *24* (5), 832–839.
[2] Hammaecher, C.; Joris, B.; Goormaghtigh, E.; Marchand-Brynaert, J. *European J. Org. Chem.* **2013**, *2013* (35), 7952–7959.

2. Chemical Synthesis and Bioconjugation Procedures

2.1 Isolation of the products

Synthesis of compound 1[3]

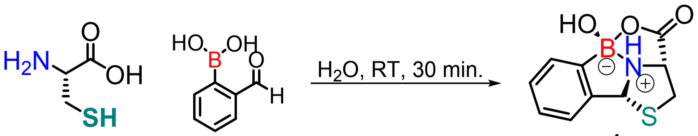

1

L-Cysteine (40.4 mg, 0.333 mmol) was dissolved in 0.5 mL of water and then added to a solution of 2-formylbenzeneboronic acid (50.0 mg, 0.333 mmol) in 2 mL of water previously prepared. The reaction occurred without stirring, at room temperature for 30 minutes. A precipitate was formed, decanted, and washed 3x with water. The crystals were dried under vacuum to give 48.9 mg of pure product. The mother liquor and washing water were combined and evaporated to give 28.8 mg of a residue, that ^{1}H-NMR analysis show that is the same product as that obtained in the crystals. Therefore, the total mass of **1** was 77.7 mg (0.333 mmol, 99% yield).

^{1}H NMR (300 MHz, DMSO-d_6) δ 8.68 (s, 1H, D_2O exchange), 7.40 – 7.31 (m, 1H), 7.29 – 7.21 (m, 3H), 7.17 – 7.10 (m, 1H), 6.08 (s, 1H), 4.77 (s, 1H, D_2O exchange), 4.65 (dd, J = 5.7, 2.5 Hz, 1H), 3.53 – 3.35 (m, 2H). **^{13}C NMR** (75 MHz, DMSO) δ 172.22 (C), 142.71 (C), 129.76 (CH), 128.16 (CH), 128.06 (CH), 122.56 (CH), 72.17 (CH), 64.79 (CH), 37.51 (CH_2). **LRMS** (m/z, ESI): 235.9 $[M+H]^+$, 217.9, 194.7. **Anal. Calcd** for $C_{10}H_{10}BNO_3S$: C, 51.10; H, 4.29; B, 4.60; N, 5.96; O, 20.42; S, 13.64. Found: C, 51.44; H, 4.28; N, 6.05; S, 13.35.

Synthesis of compound 2

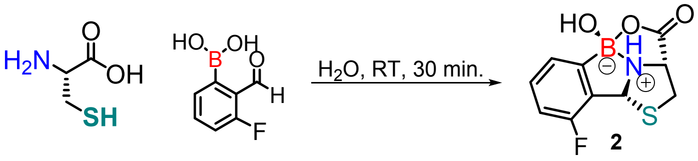

2

L-Cysteine (23.3mg, 0.192 mmol) was dissolved in 0.5 mL of water and added to a solution of 3-fluoro-2-formylbenzeneboronic acid (32.4 mg, 0.192 mmol) in 1 mL of water previously prepared. The reaction occurred without stirring, at room temperature for 30 min. A precipitate was formed, decanted, and washed 3x with water. The crystals were dried under vacuum to give 41.5 mg of pure product. The mother liquor and washing water were combined and evaporated to give 3.5 mg of a residue, that ^{1}H-

[3] CCDC 1473605 contains the crystallographic data for compound **1** that can be obtained from the CCDC via www.ccdc.cam.ac.uk/data_request/cif.

NMR analysis show that is the same product as that obtained in the crystals. Therefore, the total mass of **2** was 44.5 mg (0.176 mmol, 91.4%. yield).

^{1}H NMR (300 MHz, DMSO-d$_6$) δ 8.80 (s, 1H), 7.40 – 7.27 (m, 1H), 7.21 – 7.16 (m, 1H), 7.10 – 7.02 (m, 1H), 6.10 (s, 1H), 4.89 (s, 1H), 4.73 (d, J = 4.2 Hz, 1H), 3.61 – 3.47 (m, 2H). **^{13}C NMR** (75 MHz, DMSO) δ 172.00 (C), 158.81 + 155.53 (CF), 130.96 + 130.88 (CH), 128.81 + 128.64 (C), 125.76 + 125.72 (CH), 114.44 + 114.19 (CH), 68.25 (CH), 64.13 (CH), 37.94 (CH2). **LRMS** (m/z, ESI): 254.6 [M+H$^+$], 236.5, 208.6, 102.6. **Anal. Calc** for $C_{10}H_9BFNO_3S$: C, 47.46; H, 3.58; N, 5.54; S, 12.67. Found: C, 48.02; H, 3.80; N, 5.60; S, 12.24.

Synthesis of compound 3

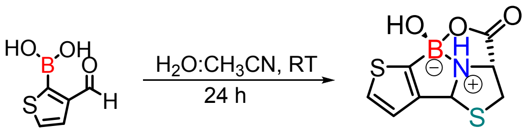

4:1 *dr*

L-Cysteine (23.3mg, 0.192 mmol) was dissolved in 0.5 mL of water:acetonitrile (1:1) and added to a solution of (3-formylthiophen-2-yl)boronic acid (30.0 mg, 0.192 mmol) in 2 mL of water:acetonitrile (1:1) previously prepared. The reaction occurred without stirring, at room temperature for 24 hours. The solvent was evaporated to a final volume of about 1 mL, a precipitate was formed, decanted and washed 3x with water. The precipitate was dried under vacuum to give 34.2 mg (0.142 mmol, 74%) of **3** as a 4:1 mixture of diastereoisomers. The mother liquor and filtrates were collected and evaporated until dryness to give 12.9 mg of a residue, that ^{1}H-NMR analysis shows that this fraction was impure. Therefore, we ignored this fraction.

^{1}H NMR (300 MHz, DMSO-d$_6$) δ 9.19 (t, J = 7.1 Hz, 0.2H, D_2O exchange), 9.01 (t, J = 7.0 Hz, 0.8H, D_2O exchange), 7.61 (d, J = 4.6 Hz, 0.8H), 7.58 (d, J = 4.8 Hz, 0.2H), 6.89 (d, J = 4.6 Hz, 0.8H), 6.85 (d, J = 4.7 Hz, 0.2H), 6.02 (d, J = 6.1 Hz, 1H), 4.99 (ls, 1H, D_2O exchange), 4.65 – 4.56 (m, 0.8H), 4.56 – 4.47 (m, 0.2H), 3.64 – 3.49 (m, 1H), 3.31 (dd, J = 12.3, 4.0 Hz, 0.8H), 3.22 (dd, J = 12.3, 4.9 Hz, 0.2H). **^{13}C NMR** (75 MHz, DMSO) δ 171.88 (C), 171.67 (C') 146.19 (C), 133.62 (C'H), 133.40 (CH), 121.75 (CH), 121.59 (C'H), 69.46 (C'H), 69.12 (CH), 65.28 (C'H), 64.65 (CH), 36.78 (CH_2), 36.01 (C'H_2). **LRMS** (m/z, ESI): 242.5 $[M+H]^+$, 216.6, 214.5, 195.6. **HRMS** Calculated for $C_9H_{10}BNNaO_3S_2$ [M-OH+MeO+Na]: 278.0093, found 278.0078.

Synthesis of compound 4

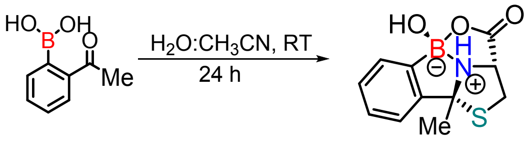

L-Cysteine (37.0 mg, 0.305 mmol) was dissolved in 0.5 mL of water and added to a solution of (2-acetylphenyl)boronic acid (50.1 mg, 0.306 mmol) in 4.1 mL of water and 0.6 mL of acetonitrile previously prepared. The reaction occurred without stirring, at room temperature for 24 hours. A precipitate was formed, decanted, and washed 3x with water. The crystals were dried under vacuum to give 27.9 mg (0.112 mmol, 37%) of **4**. The mother liquor and washing water were combined and evaporated to give 12.9 mg of a residue, that ^{1}H-NMR analysis show that this fraction was impure. Therefore, we ignored this fraction.

^{1}H NMR (300 MHz, DMSO-d$_6$) δ 8.40 (d, J = 7.3 Hz, 1H), 7.36 – 7.30 (m, 1H), 7.29 – 7.21 (m, 3H), 4.79 (ls, 1H), 4.71 – 4.59 (m, 1H), 3.60 (dd, J = 12.3, 6.1 Hz, 1H), 3.29 (dd, J = 12.3, 3.5 Hz, 1H), 1.96 (s, 3H). **^{13}C NMR** (75 MHz, DMSO) δ 172.06 (C), 147.47 (C), 129.45 (CH), 128.35 (CH), 127.93(CH), 121.49 (CH), 84.16 (C), 66.14 (CH), 36.79 (CH_2), 29.13 (CH_3). **LRMS** (m/z, ESI): 250.1 $[M+H]^+$, 232.6, 231.5, 102.6. **HRMS** Calculated for $C_{11}H_{13}BNO_3S$: 250.0709, found 250.0693.

Synthesis of compound 28

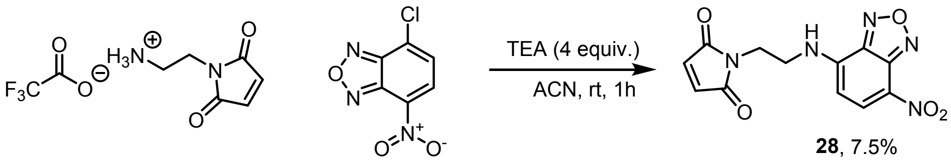

28, 7.5%

In a round bottom flask, under inert atmosphere, 4-Chloro-7-nitro-2,1,3-benzoxadiazole (NBD-Cl) (88 mg, 0.44 mmol) was dissolved in Acetonitrile (9 mL). After a solution of TEA (246 µL, 1.763 mmol) and 2-(2,5-dioxo-2,5-dihydro-1H-pyrrol-1-yl)ethan-1-aminium 2,2,2-trifluoroacetate (224 mg, 0.881 mmol)[4] in Acetonitrile (4.5 mL), was added and the solution was stirred at room temperature for 1 h. The reaction mixture was concentrated and the residue was purified by column chromatography (silica gel, hexane:EtOAc (3:1 - 1:1)) to afford **28** (10.1 mg, 0.033 mmol, 7.5 % yield).

^{1}H NMR (300 MHz, DMSO-*d*$_6$) δ 9.43 (s, 1H), 8.56 (d, *J* = 8.9 Hz, 1H), 7.03 (s, 2H), 6.47 (d, *J* = 8.9 Hz, 1H), 3.81 – 3.56 (m, 4H). **^{13}C NMR** (75 MHz, DMSO) δ 170.96 (CO), 145.02 (C), 144.46 (C), 144.03 (C), 137.97 (CH), 134.68 (CH), 121.20 (C), 99.16 (CH), 41.38 (CH_2), 35.32 (CH_2).

[4] Prepared according to the reported procedure for this compound: Schäfer, B.; Orbán, E.; Borics, A.; Huszár, K.; Nyeste, A.; Welker, E.; Tömböly, C. *Bioconjug. Chem.* **2013**, *24* (10), 1684–1697.

2.2 UV-vis experiments:

2.2.1 - UV-vis spectra

With benzaldehyde:

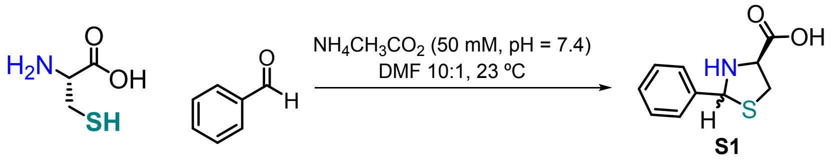

Reaction buffer: was prepared by mixing Ammonium acetate solution **pH 7.4** with dimethylformamide (DMF) in 10:1 ratio (vol:vol).

Reaction buffer (2.99 mL) was pipetted into the cuvette, followed by benzaldehyde (60 mM solution in DMF,10 µL, 0.60 µmol). The solution was mixed and a spectrum was measured. The reaction was then initiated by addition of L-cysteine (60 mM solution in DMF:H_2O 1:1,15 µL, 0.90 µmol), the cuvettes were capped, shaken and the and spectra was measured.

S1 is a known compound and was obtained according to the corresponding procedure as the reported mixture of diastereoisomers.[5]

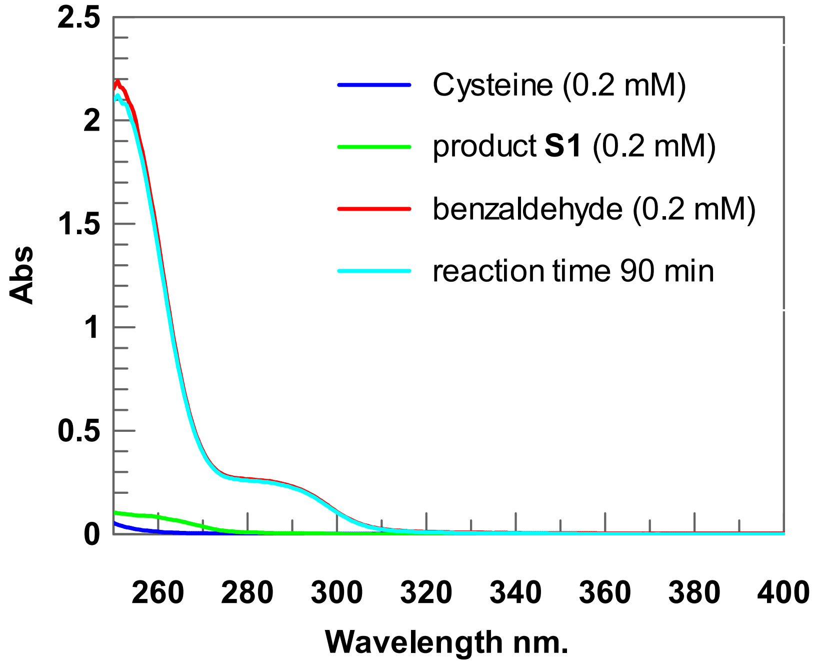

Figure S 1 - Absorvance spectra for cysteine, benzaldehyde, **S1** and at 90 min reaction time in reaction buffer.

---

[5] Khan, K. M.; Ullah, Z.; Lodhi, M. A.; Ali, M.; Choudhary, M. I.; Rahman, A. ur; Haq, Z. ul. *Mol. Divers.* **2006**, *10* (2), 223–231.

With 2FBBA:

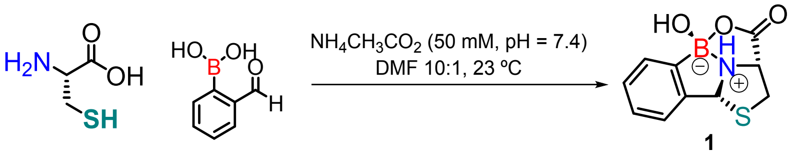

Reaction buffer (2.99 mL) was pipetted into the cuvette, followed by 2FBBA (60 mM solution in DMF,10 µL, 0.60 µmol). The solution was mixed and a spectrum was measured. The reaction was then initiated by addition of L-cysteine (60 mM solution in DMF:H_2O 1:1,15 µL, 0.90 µmol), the cuvettes were capped, shaken and the and spectra was measured.

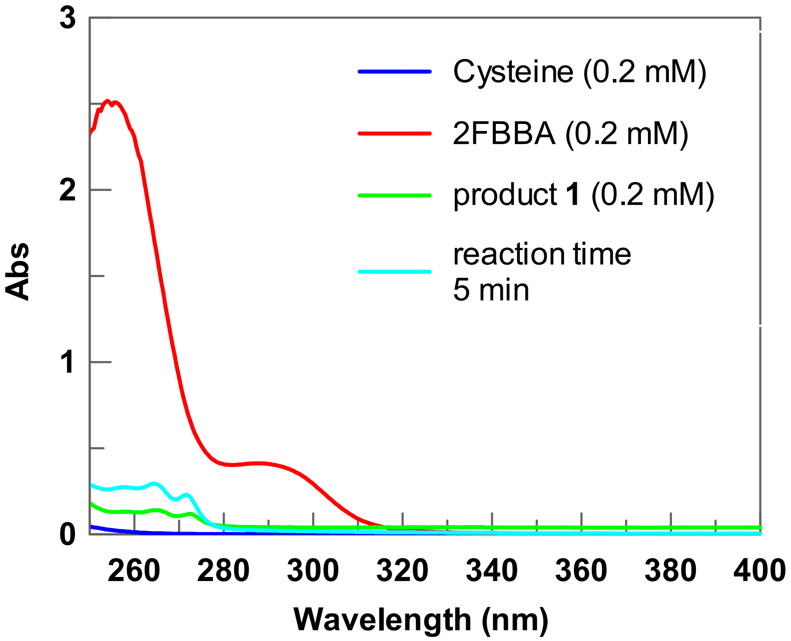

Figure S 2 - Absorbance spectra of cysteine, 2FBBA, product **1** and 5 min reaction time in the reaction buffer.

2.2.2 Determination of rate constant

Reactions were performed under pseudo-first-order conditions in quartz cuvettes (50 mm path length; total volume ~3 mL) with solutions pre-equilibrated at room temperature (23 °C). Reaction buffer (3 ml) was pipetted into the cuvette, followed by 2-formylphenylboronic acid (15 µL, 3.00 µmol) stock solution. The solution was mixed. The reaction was then initiated by addition of L-cysteine (in H_2O) (10, 15, 20, 25

or 30 mM) (15 µL). The cuvettes were capped, shaken and the absorbance at 288 nm was measured repeatedly at 1 second intervals over 150 seconds.

We measure the reaction adding the following concentrations of L-cysteine (15 µL in H_2O) in triplicate:

10 mM (concentration of cysteine in reaction mixture = 50 µM)

15 mM (concentration of cysteine in reaction mixture = 75 µM)

20 mM (concentration of cysteine in reaction mixture = 100 µM)

25 mM (concentration of cysteine in reaction mixture = 125 µM)

30 mM (concentration of cysteine in reaction mixture = 140 µM)

The k_{obs} (s^{-1}) values were calculated using the GraFit 5 software package. The k_{obs} values were then plotted against the concentration of cysteine to yield the second order rate constant (k_2, $M^{-1}s^{-1}$) from the slope of the line, to give k_2 = 2.38 ± 0.23 x 10^2 $M^{-1}s^{-1}$

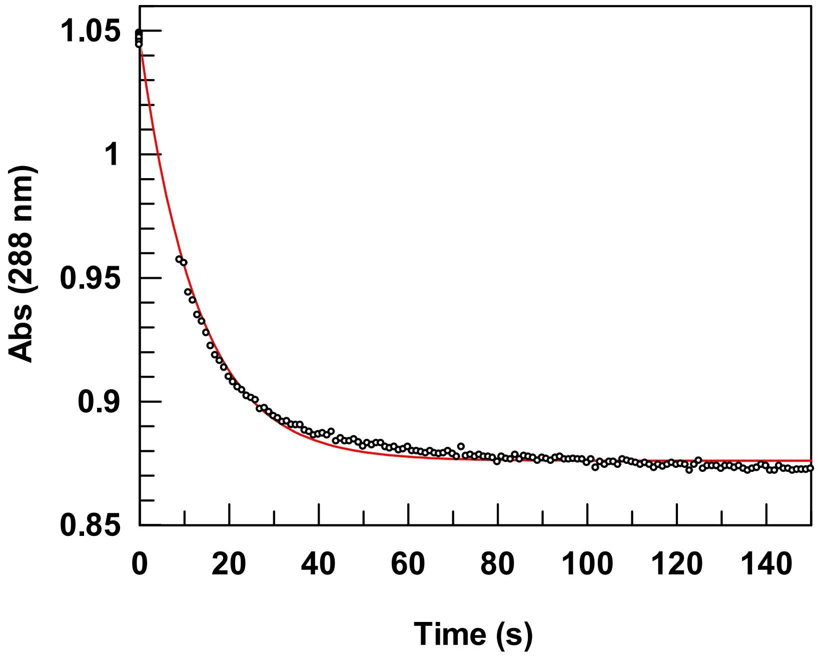

Figure S 3 - Absorbance of the reaction at 288 nm for 150 s. (k_{obs} = 7.72 x 10^{-2} s^{-1}).

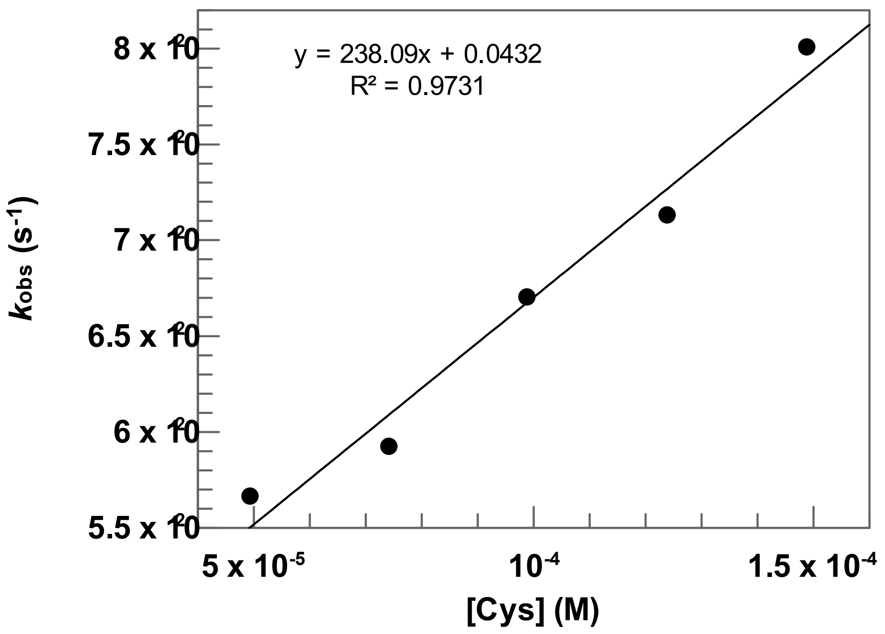

Figure S 4 - k_{obs} values plotted against the concentration of cysteine to yield the second order rate constant (k_2, $M^{-1}s^{-1}$) from the slope of the line.

2.3 Product stability:

Product stability at pH 7.4:

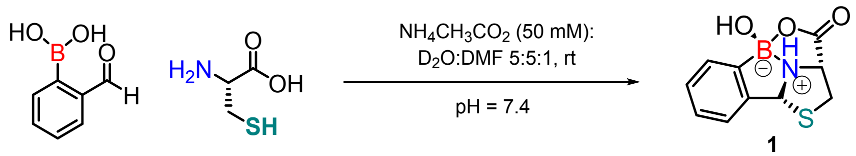

1

To a solution of 2-formylphenylboronic acid (5.0 mg, 0.033 mmol) in a mixture of Acetate buffer 50mM (0.25 ml):D_2O (0.25 ml) and DMF (0.025 ml) at pH = 7.4 was added L-cysteine (3.4 mg, 0.028 mmol). After the formation of the product at (about 90% conversion at 2min), new spectra were taken at 24h, 4 and 7days. No degradation of the product is observed.

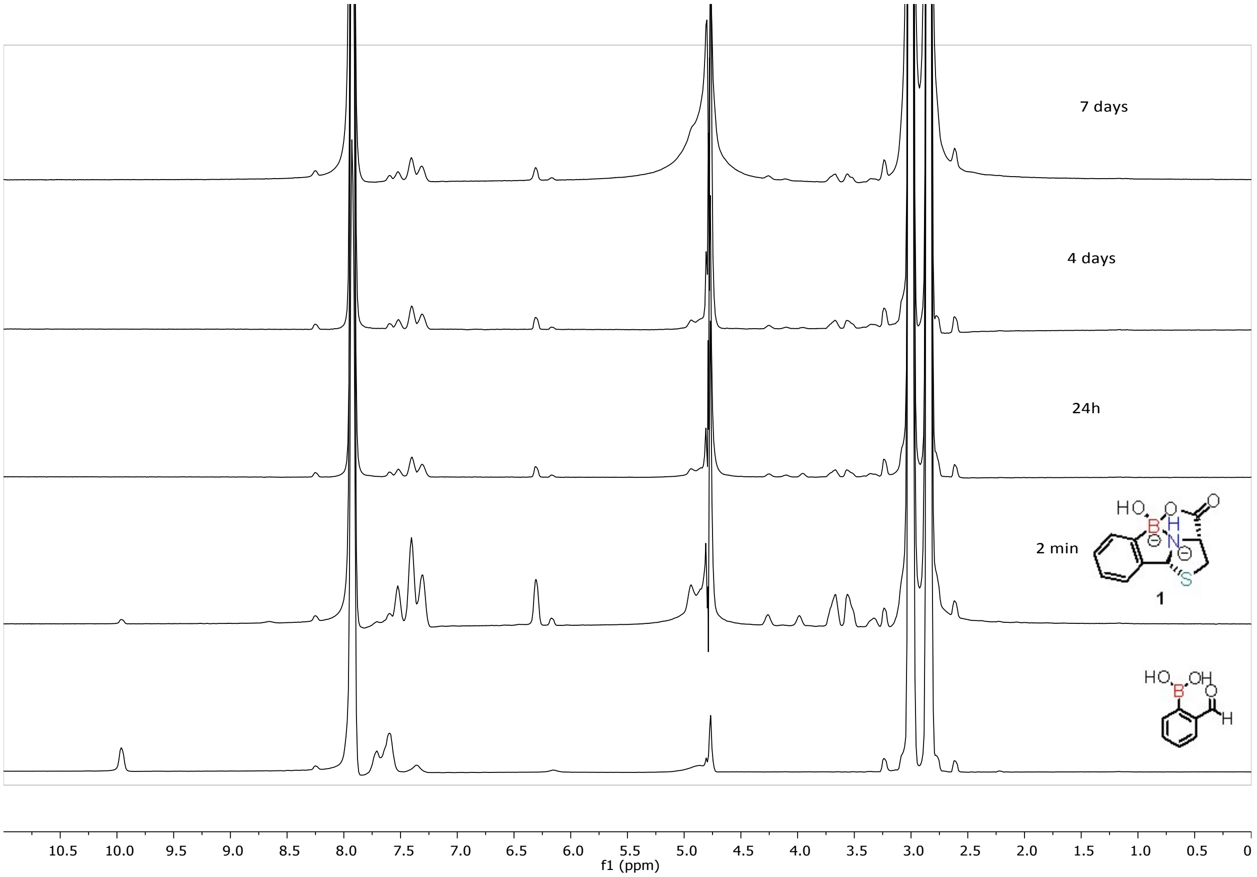

Figure S 5 - ^{1}H-NMR spectra of 2FBBA and product **1** in Acetate buffer 50mM (pH=7.4) and stability of the product overtime in those conditions.

Product stability at pH 4.5:

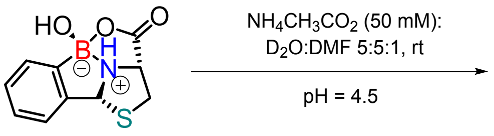

Compound **1** (2 mg, 8.51 µmol) was suspended in a mixture of Acetate buffer 50mM (0.25 ml):D_2O (0.250 ml) and DMF (0.025 ml) at pH=4.5. And ^{1}H-NMR was measured at: 30 min, 24 h, 4 and 7 days. No degradation of **1** (with a characteristic peak at δ 6.31 (s, 1H)) is observed by the appearance of new products in the NMR, however the product did start to precipitate overtime, and in the 7th day, almost no product was in solution.

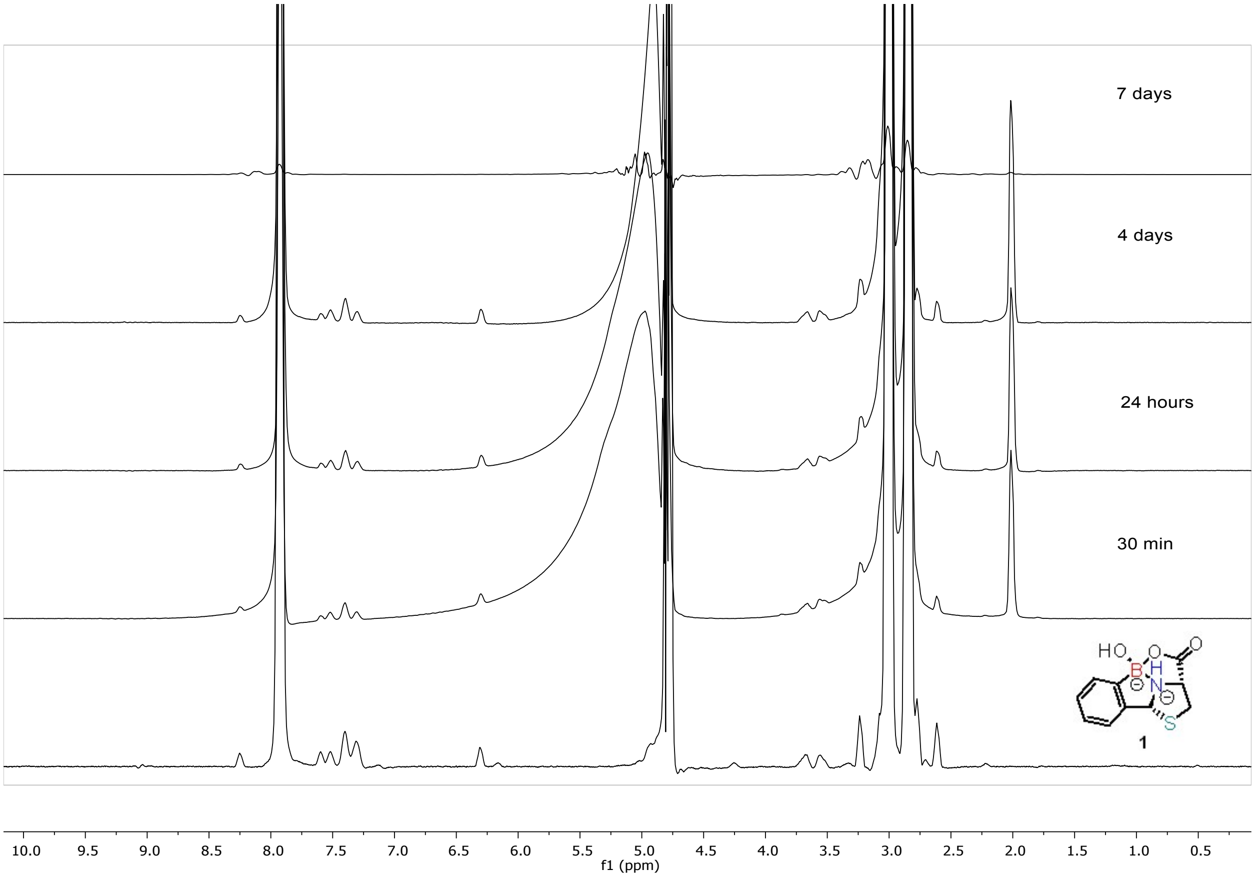

Figure S 6 - ^{1}H-NMR spectra of product **1** in Acetate buffer 50mM (pH=7.4) and stability of the product overtime at pH=4.5.

Product stability at pH 9.0:

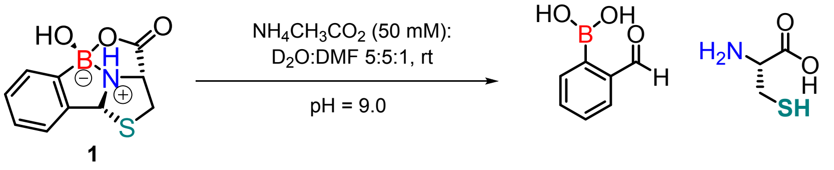

Compound **1** (2.0 mg, 8.5 µmol) was suspended in a mixture of Acetate buffer 50mM (0.25 ml):D_2O (0.250 ml) and DMF (0.025 ml) at pH=9.0. And ^{1}H-NMR was measured at: 30 min, 24 h, 4 days and 7 days. Product **1** (with a characteristic peak at δ 6.31 (s, 1H)) disappear and the characteristic peak of 2FBBA start to emerge after only 30 min.

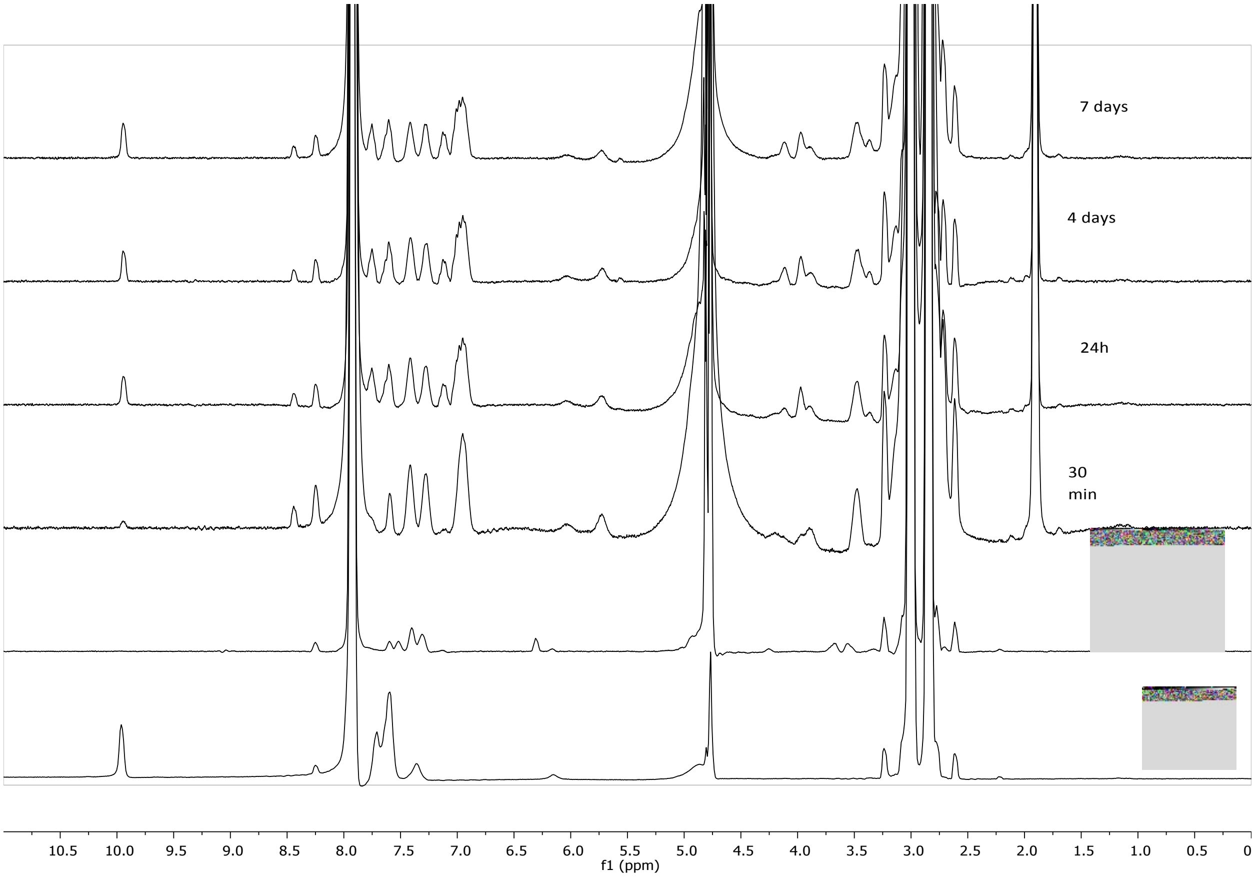

Figure S 7 - ^{1}H-NMR spectra of 2FBBA, product **1** in Acetate buffer 50mM (pH=7.4), and stability of the product overtime at pH=9.0.

2.4 Assays with model dipeptides

General procedure using TCEP (exemplified for the case of Cys-Ala-OMe)

A solution of Cys-Ala-OMe (15 mM in DMF,5.0 µL, 0.075 µmol) was added to acetate buffer 20 mM at pH 7.0, with 1% DMF (750 µL) followed by Tris(2-carboxyethyl)phosphine hydrochloride (TCEP) (30 mM in water, 7.5 µL, 0.225 µmol) and the solution mixed for 2 hours at 25 ºC. Then 2FBBA (30 mM in DMF, 7.5 µL, 0.225 µmol) was added and the mass checked in Positive Mode of ESI-MS.

General procedure without (exemplified for the case of Cys-Tyr-OMe)

A solution of Cys-Tyr-OMe (15 mM in DMF,5.0 µL, 0.075 µmol) was added to acetate buffer 20 mM at pH 7.0, with 1% DMF (750 µL), then 2FBBA (30 mM in DMF, 7.5 µL, 0.225 µmol) was added and the mass checked in Positive Mode of ESI-MS.

Cys-Ala-OMe

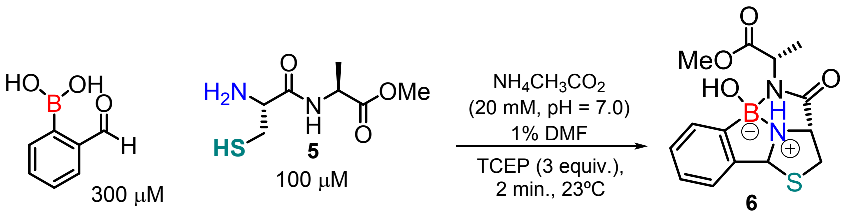

6
m/z 320
(*m/z* [M-OH] = 303)

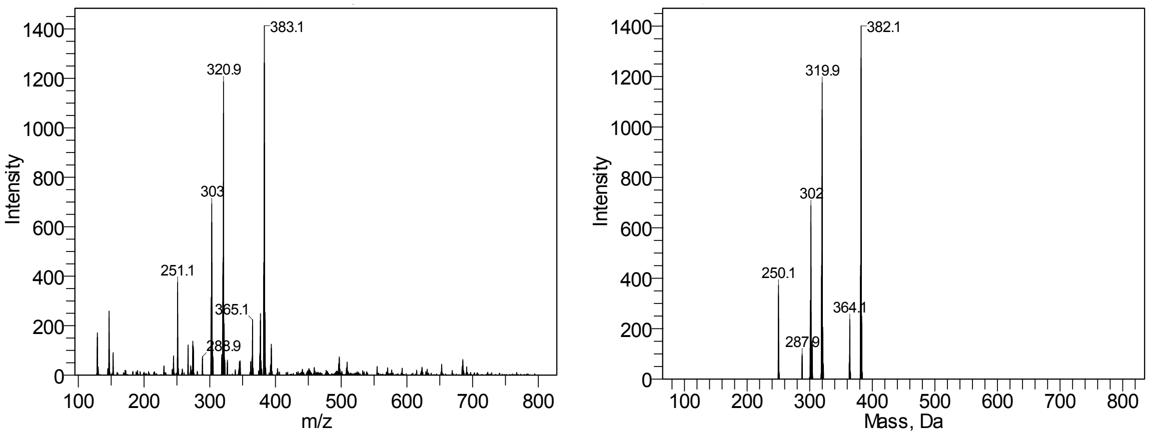

Figure S 8 - Raw (left) and deconvoluted (right) spectra of the reaction of Cys-Ala-OMe and 2FBBA at 2 min. Peaks at 250 and 382 m/z correspond to TCEP and a product between TCEP and 2FBBA respectively.

Cys-Gly-OMe

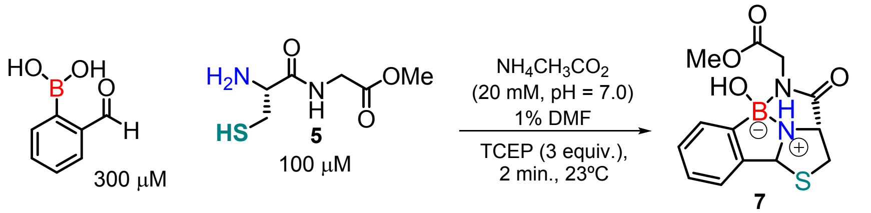

7
m/z 306
(*m/z* [M-OH] = 289)
(*m/z* [M+DMF] = 362)

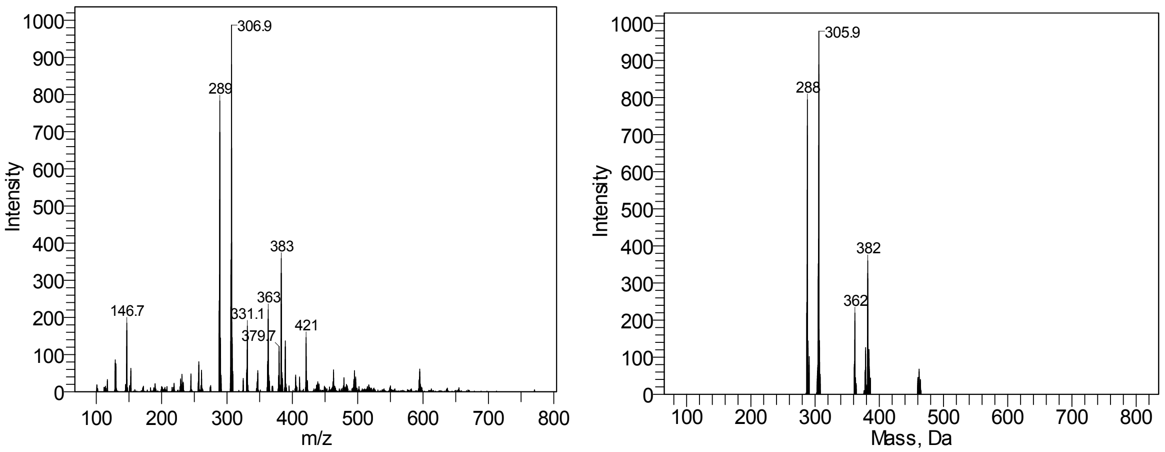

Figure S 9 - Raw (left) and deconvoluted (right) spectra of the reaction of Cys-Gly-OMe and 2FBBA at 2 min. Peaks at 250 and 382 m/z correspond to TCEP and a product between TCEP and 2FBBA respectively.

Cys-Leu-OMe

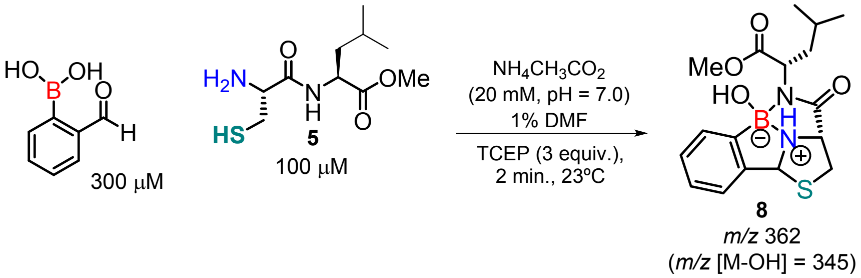

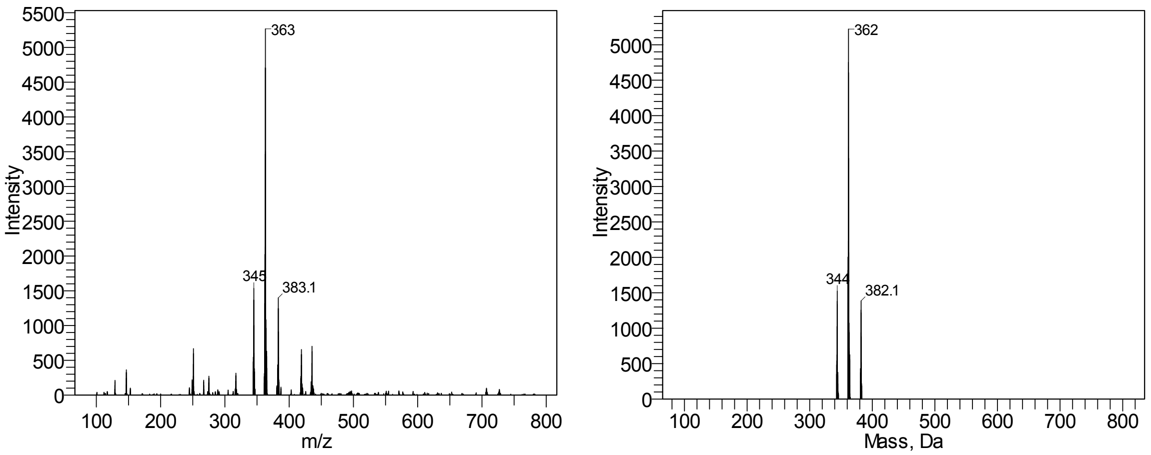

Figure S 10 - Raw (left) and deconvoluted (right) spectra of the reaction of Cys-Leu-OMe and 2FBBA at 2 min. Peaks at 250 and 382 m/z correspond to TCEP and a product between TCEP and 2FBBA respectively.

Cys-Phe-OMe

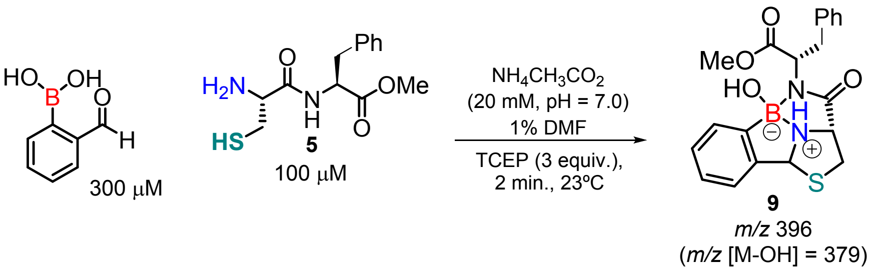

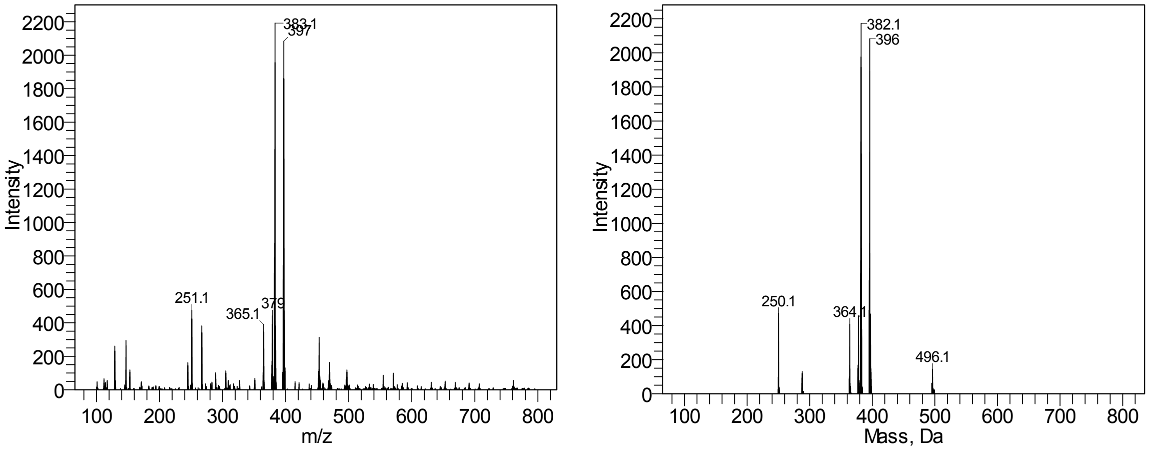

Figure S 11 - Raw (left) and deconvoluted (right) spectra of the reaction of Cys-Phe-OMe and 2FBBA at 2 min. Peaks at 250 and 382 m/z correspond to TCEP and a product between TCEP and 2FBBA respectively.

Cys-Tyr-OMe

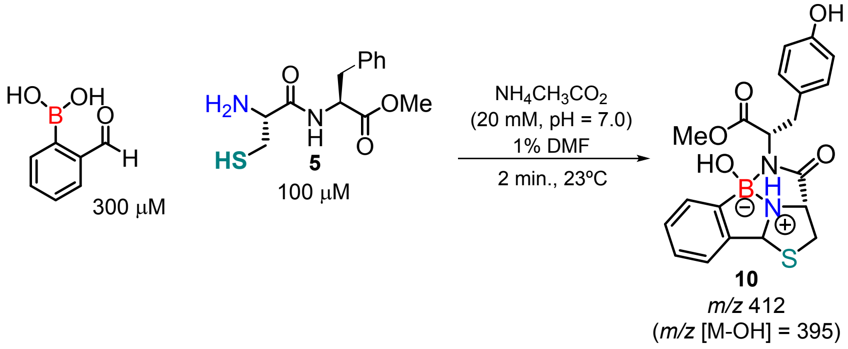

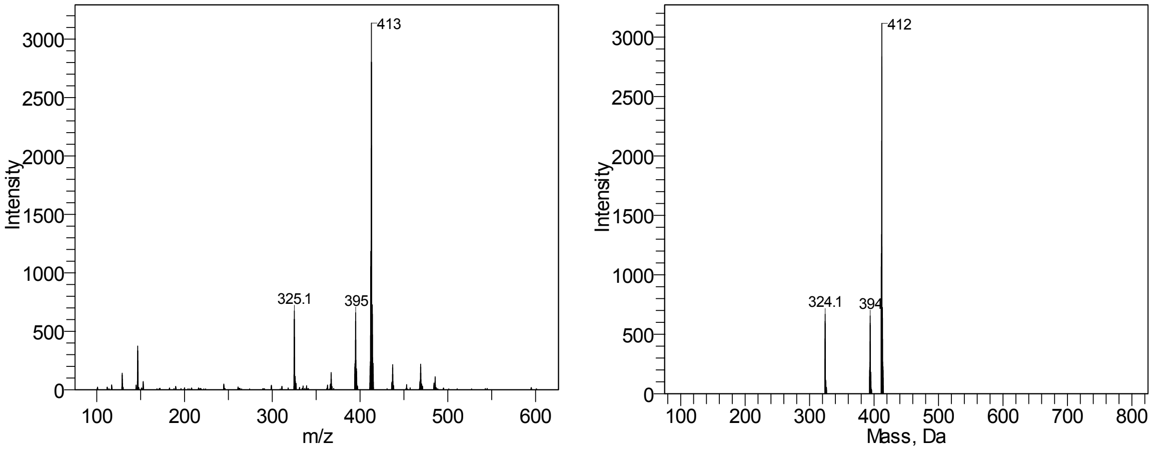

Figure S 12 - Raw (left) and deconvoluted (right) spectra of the reaction of Cys-Tyr-OMe and 2FBBA at 2 min. Peaks at 250 and 382 m/z correspond to TCEP and a product between TCEP and 2FBBA respectively.

Cys-Lys-OMe

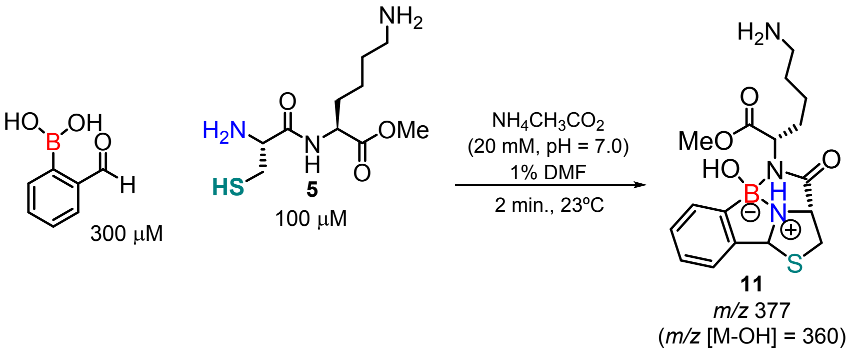

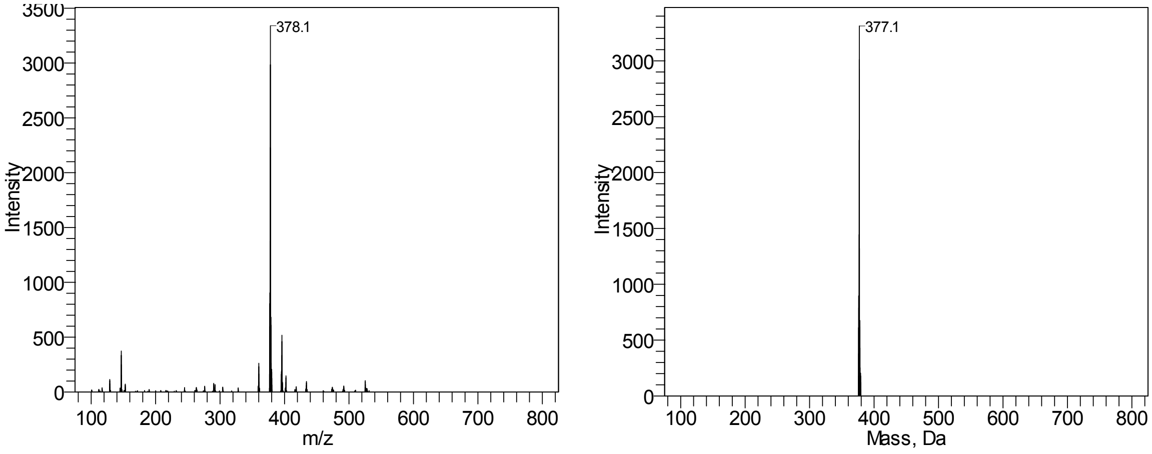

Figure S 13 - Raw (left) and deconvoluted (right) spectra of the reaction of Cys-Lys-OMe and 2FBBA at 2 min. Peaks at 250 and 382 m/z correspond to TCEP and a product between TCEP and 2FBBA respectively.

Cys-His-OMe

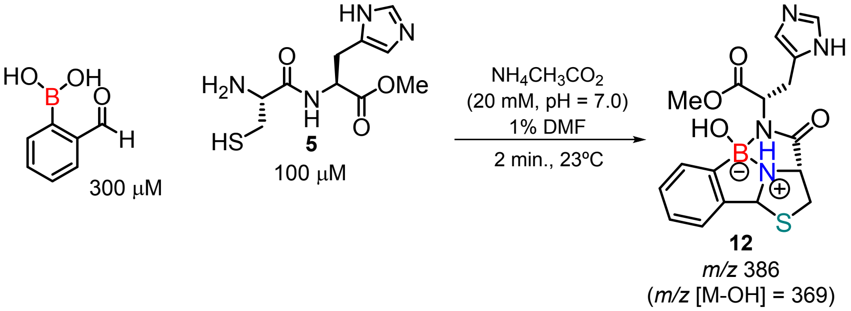

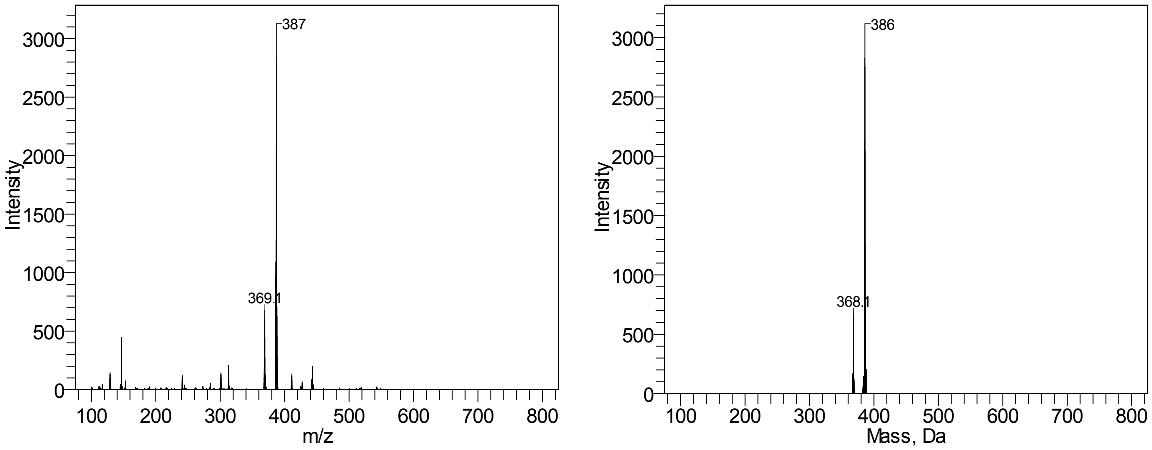

Figure S 14 - Raw (left) and deconvoluted (right) spectra of the reaction of Cys-His-OMe and 2FBBA at 2 min. Peaks at 250 and 382 m/z correspond to TCEP and a product between TCEP and 2FBBA respectively.

Cys-Ser-OMe

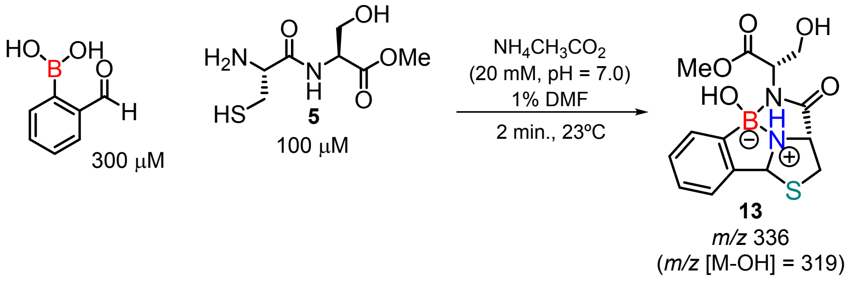

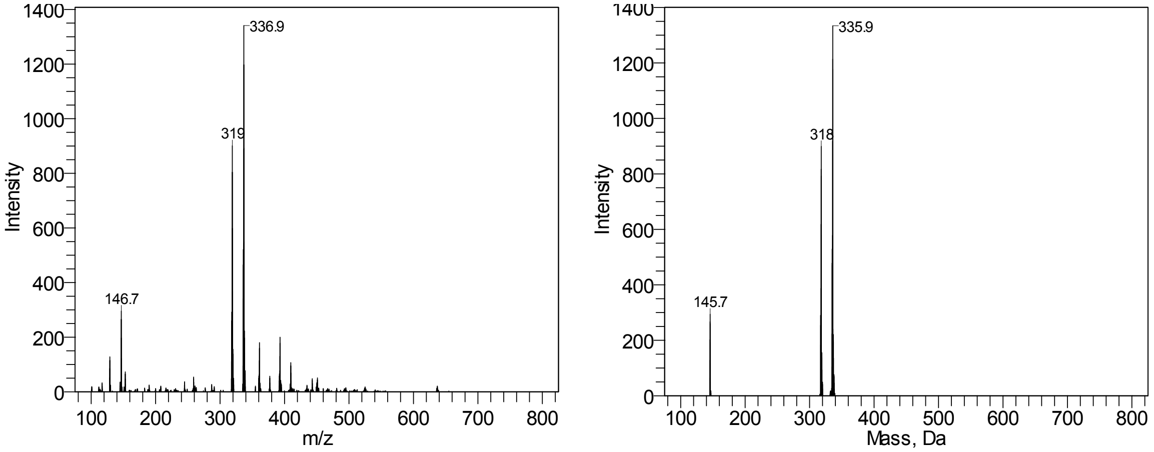

Figure S 15 - Raw (left) and deconvoluted (right) spectra of the reaction of Cys-Ser-OMe and 2FBBA at 2 min. Peaks at 250 and 382 m/z correspond to TCEP and a product between TCEP and 2FBBA respectively.

Cys-Glu-OMe

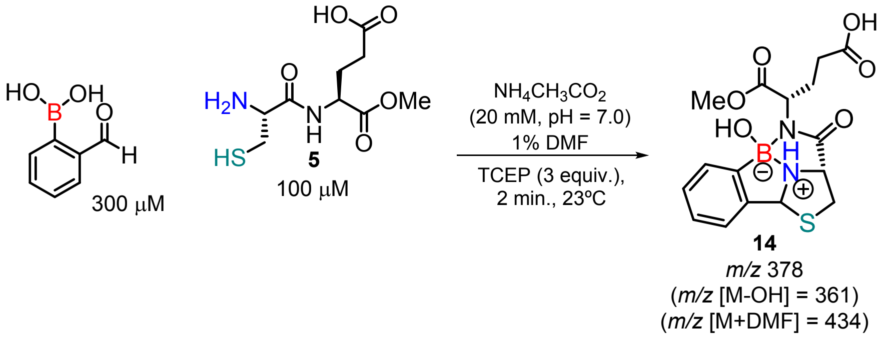

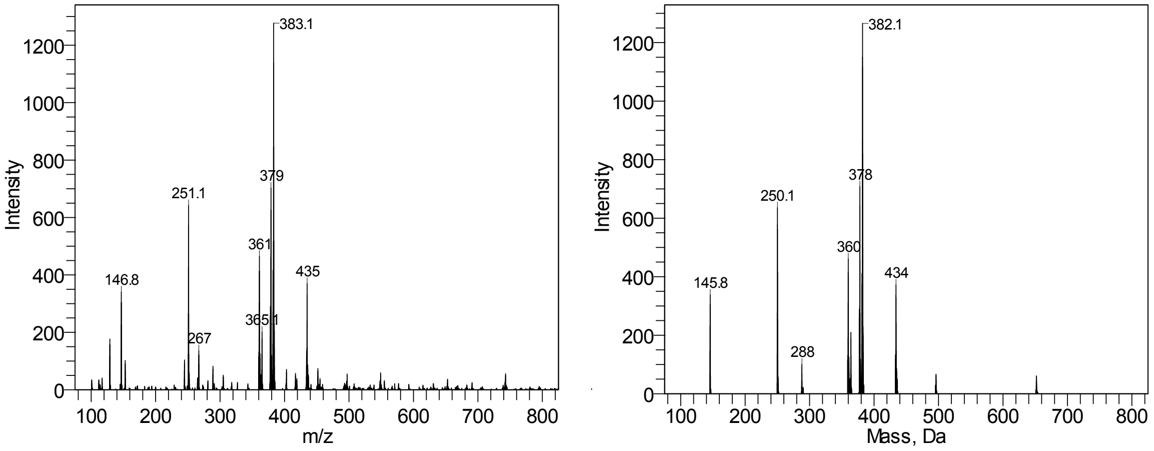

Figure S 16 - Raw (left) and deconvoluted (right) spectra of the reaction of Cys-Glu-OMe and 2FBBA at 2 min. Peaks at 250 and 382 m/z correspond to TCEP and a product between TCEP and 2FBBA respectively.

2.5 Assays with peptides

C-Ovalbumin+2FBBA

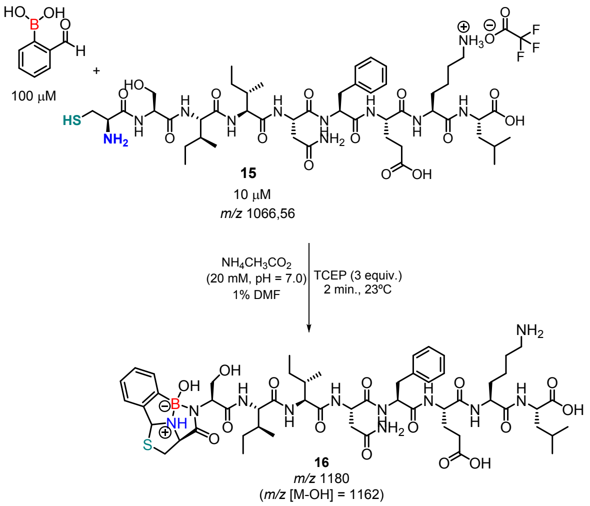

A solution of C-Ovalbumin (1mg/mL, 5.9 µL, 0.005 µmol) was added to Acetate buffer 20 mM pH = 7.0 + 1% DMF (0.5 ml) followed by Tris(2-carboxyethyl)phosphine hydrochloride (TCEP) (3.5 mM in water, 1 mg/mL, 4.3 µL, 0.015 µmol) and the solution mixed for 2 hour at 25 ºC. Then 2FBBA (10 mM in DMF, 5.0 µL, 0.050 µmol) was added and the mass checked in Positive Mode of ESI-MS.

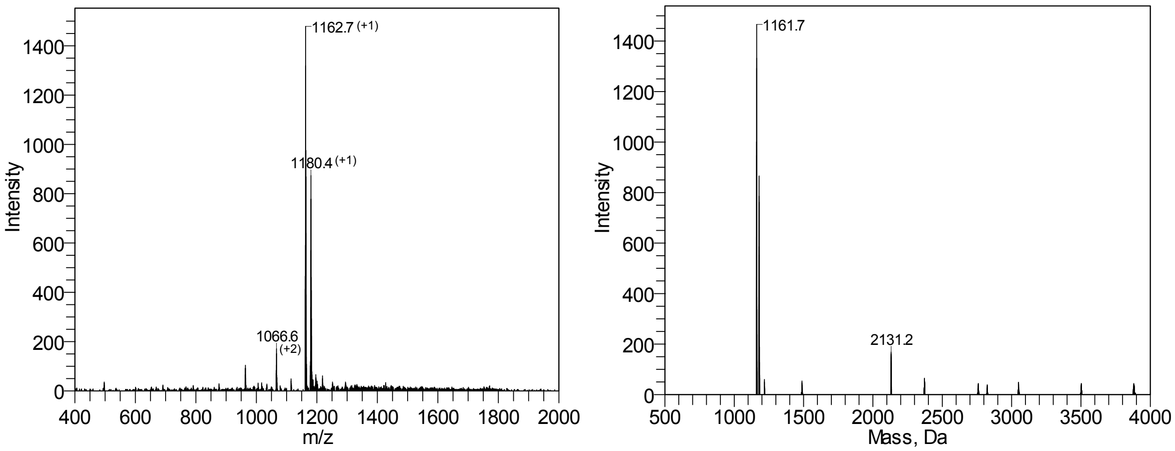

Figure S 17 - Raw (left) and deconvoluted (right) spectra of the reaction of C-Ovalbumin and 2FBBA at 2 min.

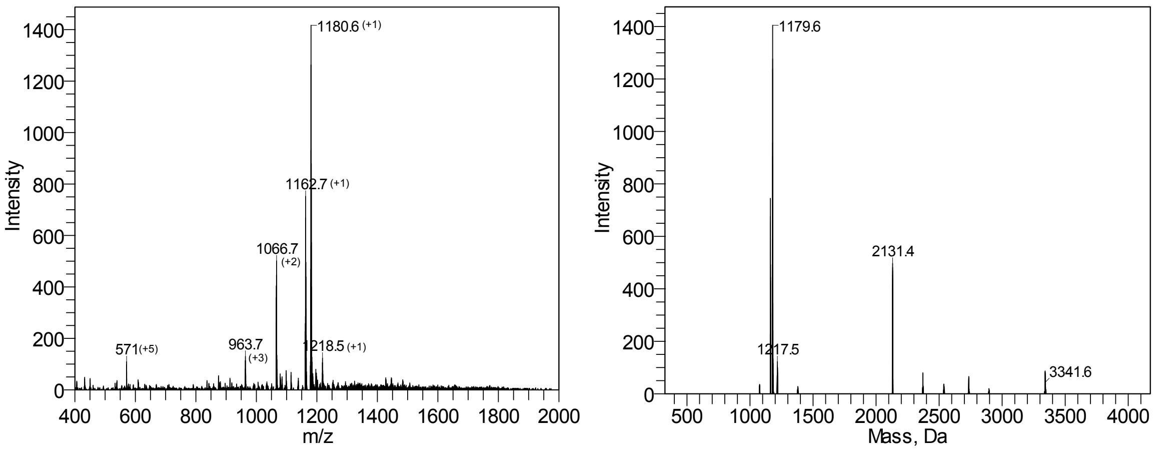

Figure S 18 - Raw (left) and deconvoluted (right) spectra of the reaction of C-Ovalbumin and 2FBBA at 24h

C-Ovalbumin+benzaldehyde

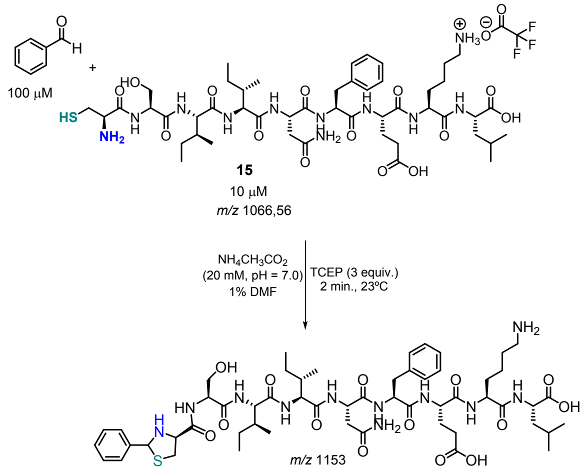

A solution of C-Ovalbumin (1mg/mL, 5.90 µL, 0.005 µmol) was added to Acetate buffer 20 mM pH = 7.0 + 1% DMF (0.5 ml) followed by Tris(2-carboxyethyl)phosphine hydrochloride (TCEP) (3.5 mM in water, 1 mg/mL, 4.3 µL, 0.015 µmol) and the solution mixed for 2 hour at 25 ºC. Then 2FBBA (10 mM in DMF, 5.0 µL, 0.050 µmol) was added and the mass checked in Positive Mode of ESI-MS.

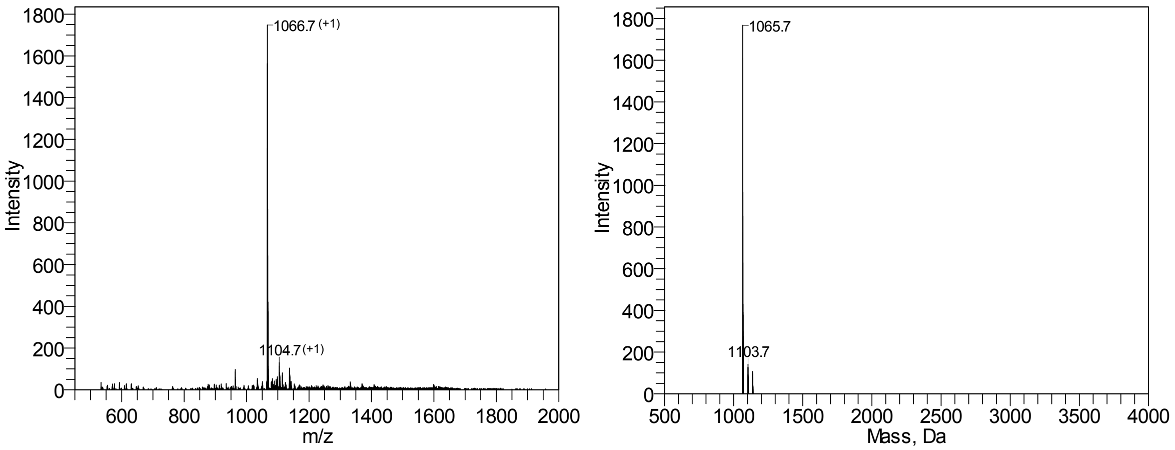

Figure S 19 - Raw (left) and deconvoluted (right) spectra of the reaction of C-Ovalbumin and benzaldehyde at 2 min.

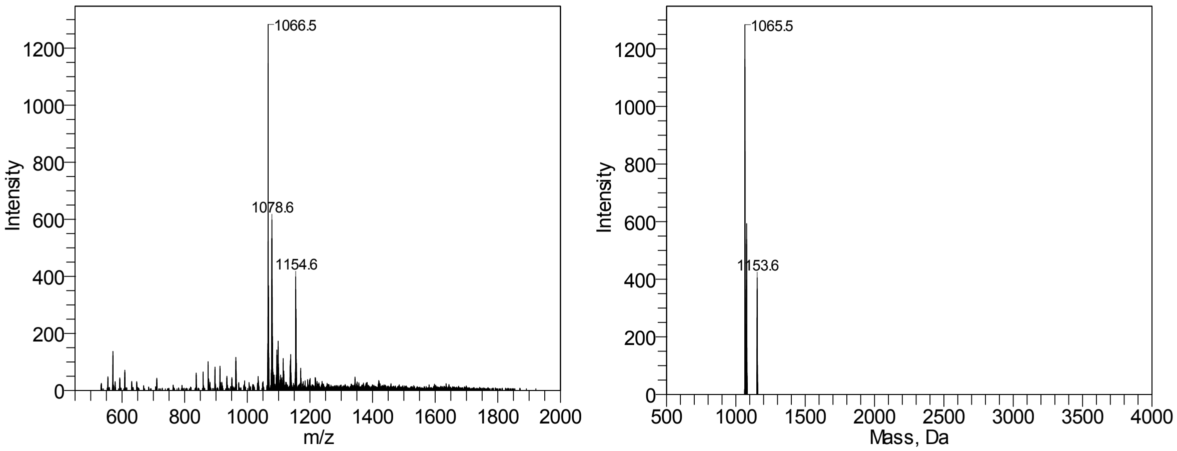

Figure S 20 - Raw (left) and deconvoluted (right) spectra of the reaction of C-Ovalbumin and benzaldehyde at 24h.

Laminin fragment + 2FBBA

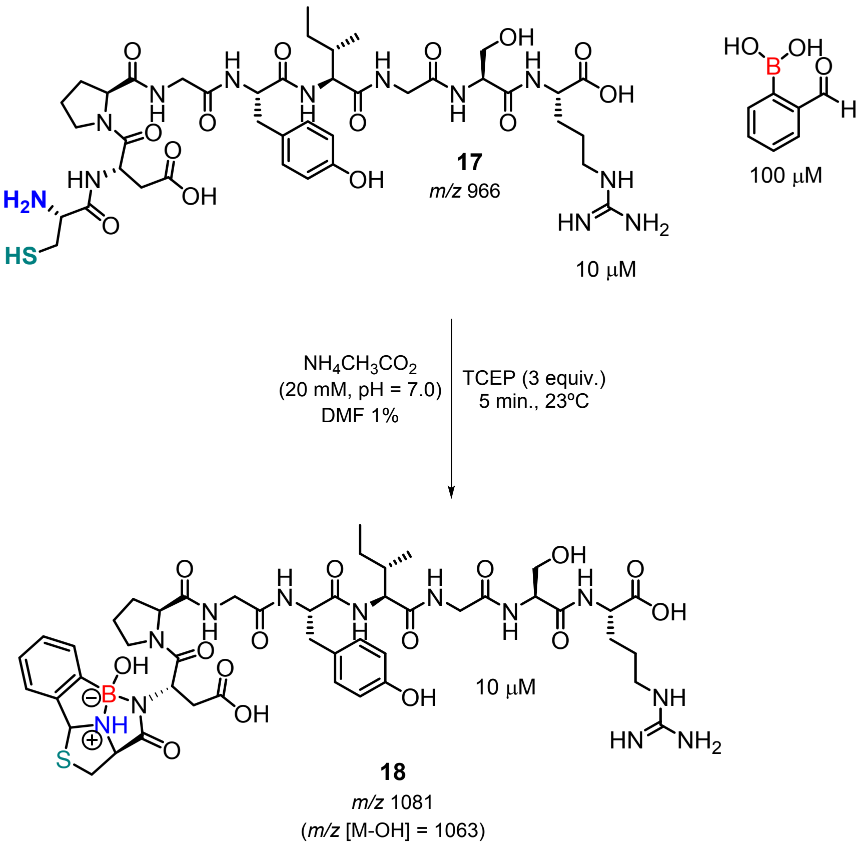

A solution of Laminin Fragment (1 mg/mL in H_2O, 0.925 mM, 5.4 µL, 0.005 µmol) was added to Acetate buffer 20 mM pH 7.0, DMF 1% (0.5 ml) followed by Tris(2-carboxyethyl)phosphine hydrochloride (TCEP) (3.5 mM in water, 1 mg/mL,4.3 µL, 0.015 µmol) and the solution mixed for 2 hours at 25 ºC. Then 2FBBA (10 mM in DMF,5.0 µL, 0.050 µmol) was added and the mass checked in Positive Mode of ESI-MS.

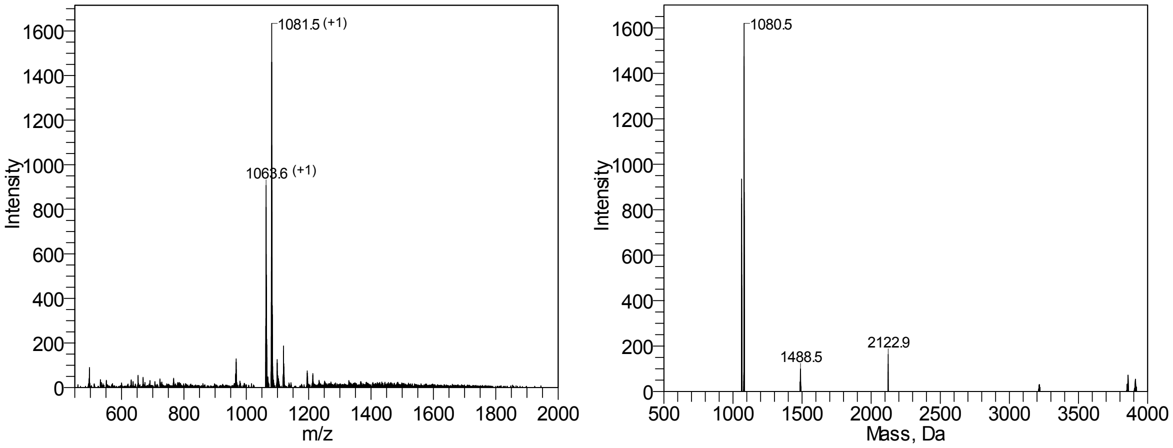

Figure S 21 - Raw (left) and deconvoluted (right) spectra of the reaction of Laminin fragment and 2FBBA at 5 min.

Laminin fragment + 2FBBA + benzyl hydroxylamine

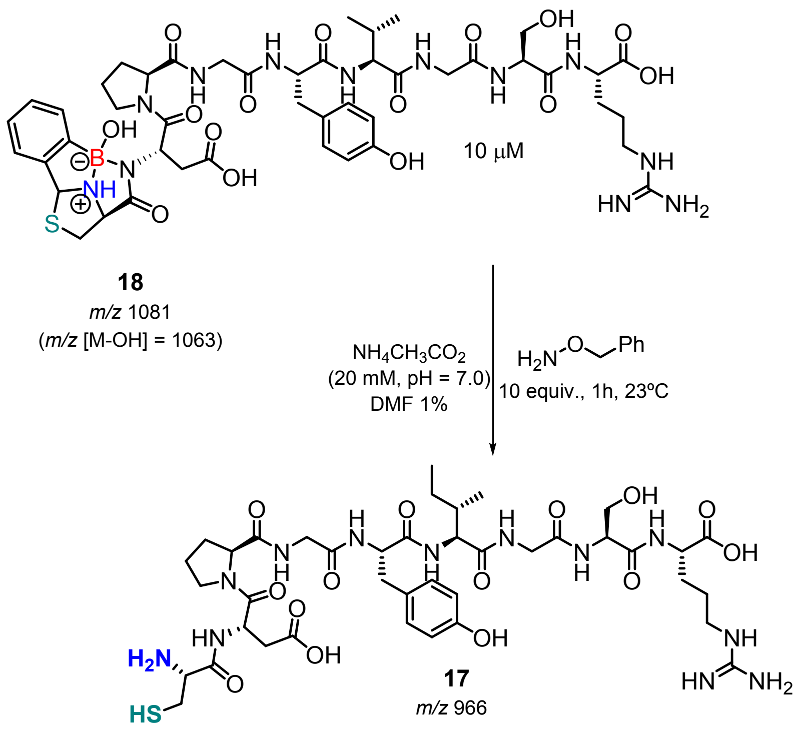

A solution of Laminin Fragment (1 mg/mL in H2O, 0.925 mM,5.4 µL, 0.005 µmol) was added to Acetate buffer 20 mM pH 7.0, DMF 1% (0.5 ml) followed by Tris(2-carboxyethyl)phosphine hydrochloride (TCEP) (3.5 mM in water, 1 mg/mL,4.3 µL, 0.015 µmol) and the solution mixed for 2 hours at 25 ºC. Then 2-2FBBA (10 mM in DMF,5.0 µL, 0.050 µmol) was added and solution mixed for 2 minutes. Finally *O*-benzylhydroxylamine hydrochloride (7.71 mM in DMF,6.5 µL, 0.050 µmol) was added, and the mass checked in Positive Mode of ESI-MS. After 1h of the addition of *O*-benzylhydroxylamine, most of the thiazolidne had been reconverted back to the free peptide.

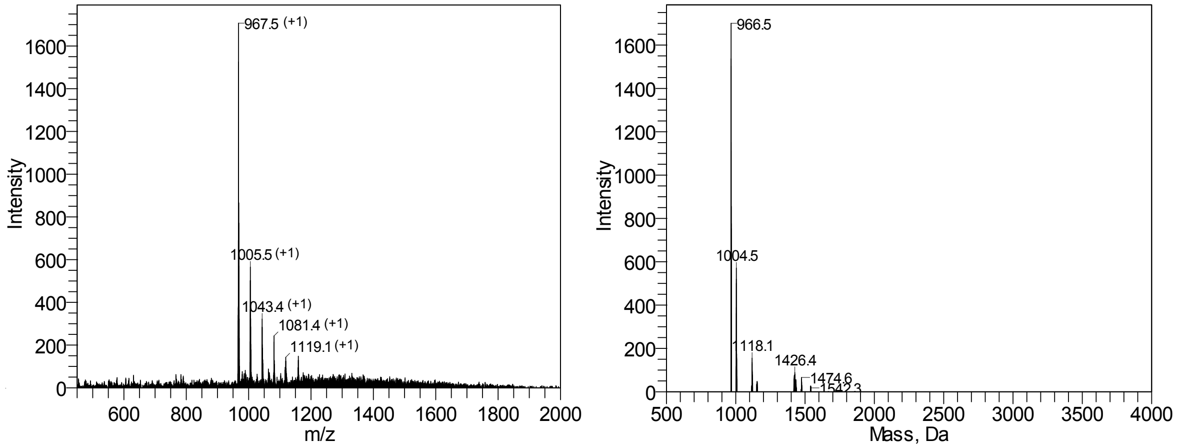

Figure S 22 - Raw (left) and deconvoluted (right) spectra of the reaction of **18** and benzyl hydroxylamine at 1hour.

Calcitonin Acetyl salmon + 20

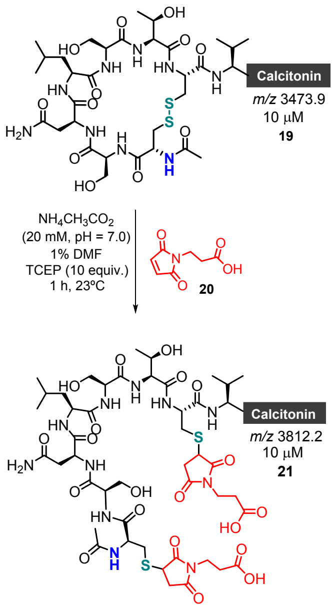

A solution of Calcitonin Acetyl salmon (0.287 mM in water, 1 mg/mL, 8.7 µL, 0.0025 µmol) was added to Acetate buffer 20 mM pH = 7 with 1% DMF (250 µL) followed by Tris(2-carboxyethyl)phosphine hydrochloride (TCEP) (3.5 mM in water, 1 mg/mL, 7.1 µL, 0.025 µmol) and the solution mixed for 2 hours. Then **20** (10 mM in DMF,5.0 µL, 0.050 µmol) was added, and the mass checked in Positive Mode of ESI-MS.

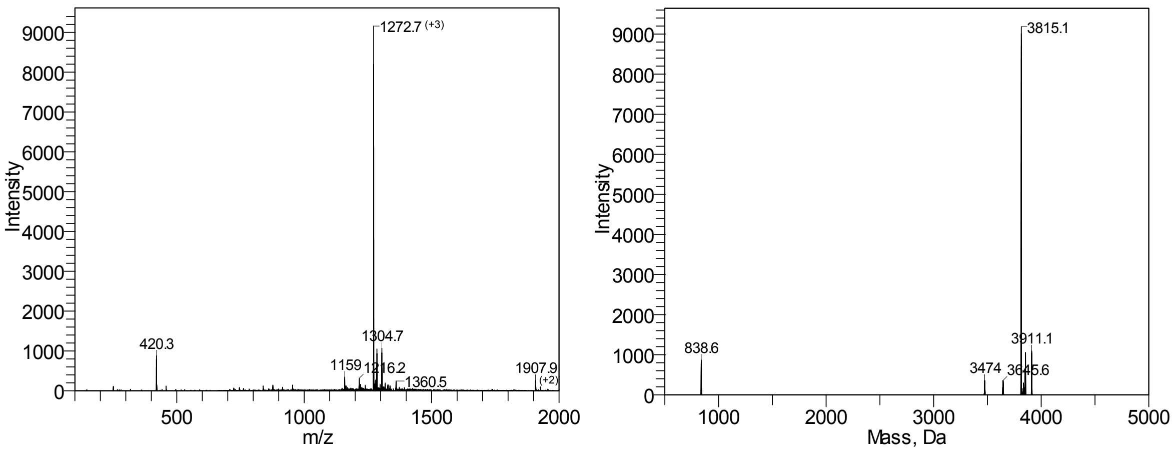

Figure S 23 - Raw (left) and deconvoluted (right) spectra of the reaction of Calcitonin Acetyl salmon and **20** at 1hour.

Calcitonin salmon + 2FBBA

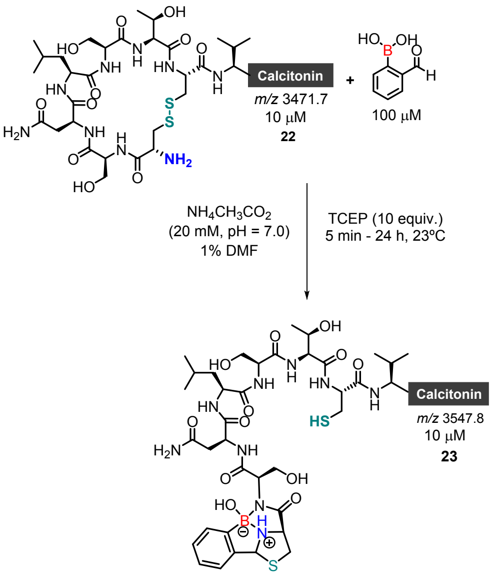

A solution of Calcitonin salmon (1mg/mL, 0.291 mM, 8.6 µL, 0.0025 µmol) was added to Acetate buffer 20 mM pH = 7 with 1% DMF (250 µL) followed by Tris(2-carboxyethyl)phosphine hydrochloride (TCEP) (3.5 mM in water, 1 mg/mL,7.1 µL, 0.025 µmol) and the solution mixed for 30 min at 25 ºC. Then 2FBBA (10 mM in DMF,2.5 µL, 0.025 µmol) was added and the mass checked in Positive Mode of ESI-MS.

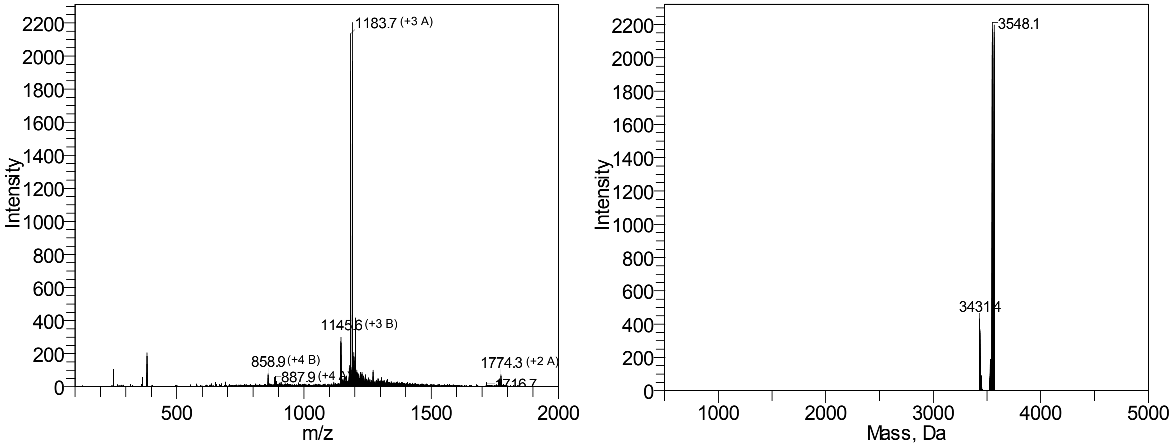

Figure S 24 - Raw (left) and deconvoluted (right) spectra of the reaction of Calcitonin salmon and 2FBBA at 5 min.

23 + 20

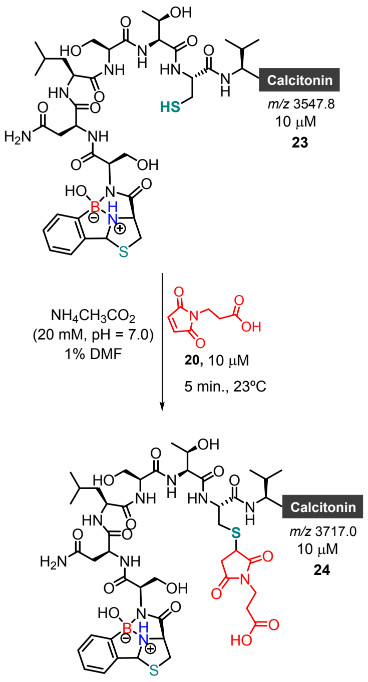

A solution of Calcitonin salmon (1mg/mL, 0.291 mM, 8.6 μL, 0.0025 μmol) was added to Acetate buffer 20 mM pH = 7 with 1% DMF (250 μL) followed by Tris(2-carboxyethyl)phosphine hydrochloride (TCEP) (3.5 mM in water, 1 mg/mL, 7.1 μL, 0.025 μmol) and the solution mixed for 30 min at 25 ºC. Then 2FBBA (10 mM in DMF,2.5 μL, 0.025 μmol) was added, the mixture stirred for another 5 min and finally 3-(2,5-dioxo-2,5-dihydro-1H-pyrrol-1-yl)propanoic acid (**20**, 5 mM in DMF,0.5 μL, 0.0025 μmol) was added and the mass checked in Positive Mode of ESI-MS.

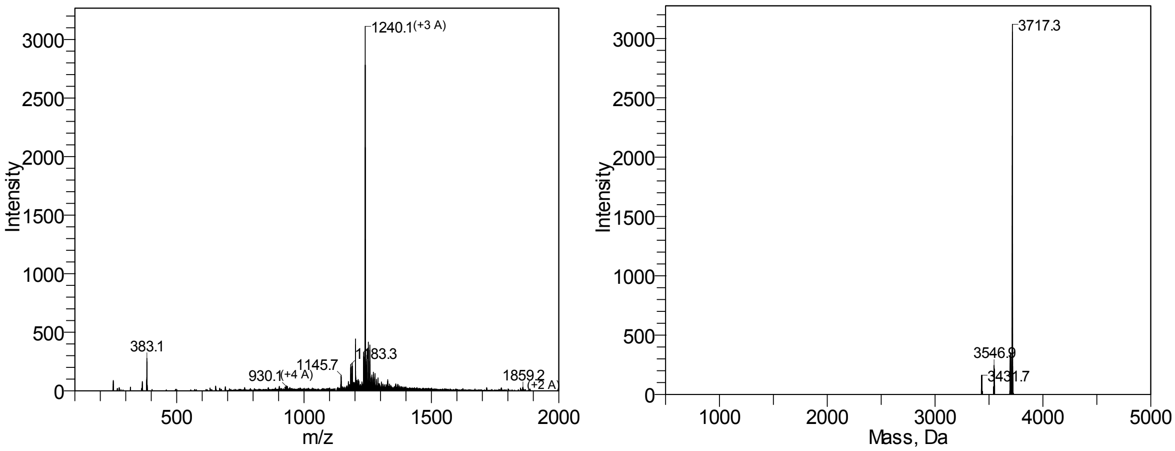

Figure S 25 - Raw (left) and deconvoluted (right) spectra of the reaction of **23** and **20** at 5 min.

23 + 25

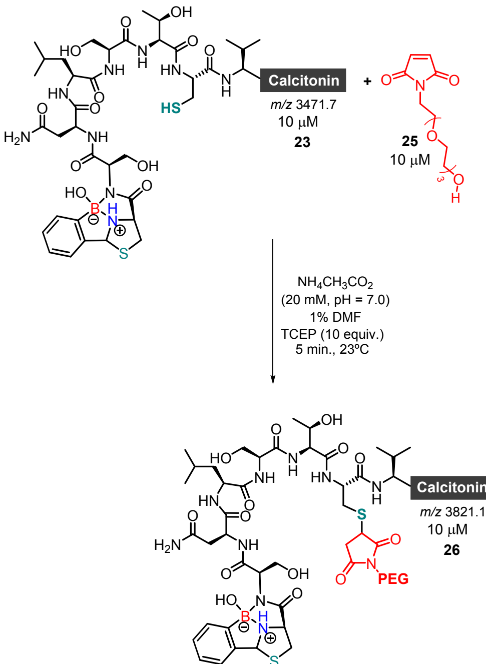

A solution of Calcitonin, salmon (1mg/mL, 0.291 mM, 8.6 µL, 0.0025 µmol) was added to Acetate buffer 20 mM pH = 7 with 1% DMF (250 µL) followed by Tris(2-carboxyethyl)phosphine hydrochloride (TCEP) (3.5 mM in water, 1 mg/mL, 7.1 µL, 0.025 µmol) and the solution mixed for 30 min at 25 ºC. Then (2-formylphenyl)boronic acid (10 mM in DMF,2.5 µL, 0.025 µmol) was added and stirred for further 2 min. Then **25** (5 mM in H_2O, 0.5 µL, 0.0025 µmol) was added and the mass checked in Positive Mode of ESI-MS.

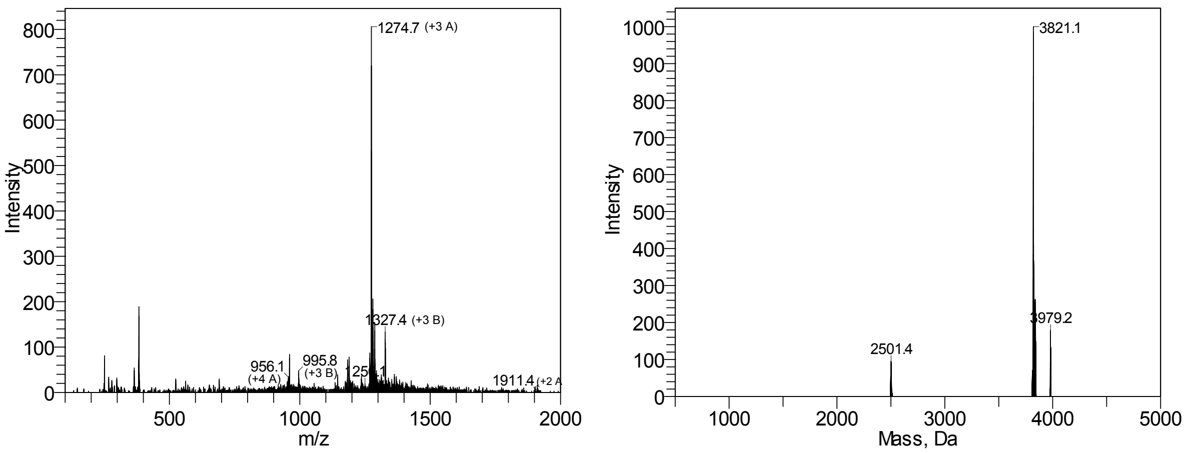

Figure S 26 - Raw (left) and deconvoluted (right) spectra of the reaction of **23** and **25** at 5 min.

26 + benzyl hydroxylamine

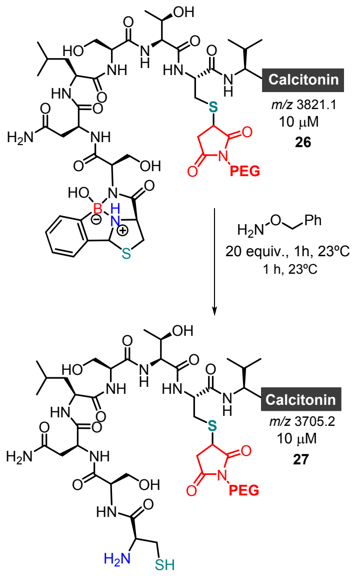

A solution of Calcitonin salmon (1mg/mL, 0.291 mM, 8.6 µL, 0.0025 µmol) was added to Acetate buffer 20 mM pH = 7 with 1% DMF (250 µL), followed by Tris(2-carboxyethyl)phosphine hydrochloride (TCEP) (3.5 mM in water, 1 mg/mL, 7.1 µL, 0.025 µmol) and the solution mixed for 30 min at 25 ºC. Then (2-formylphenyl)boronic acid (10 mM in DMF,2.5 µL, 0.025 µmol) was added and stirred for further 2 min. Then **25** (5 mM in H_2O, 0.5 µL, 0.0025 µmol) was added, stirred another 5 min and (benzyloxy)chloro-l5-azane (3.24 µL, 0.025 µmol) was added. Then the mass checked in Positive Mode of ESI-MS.

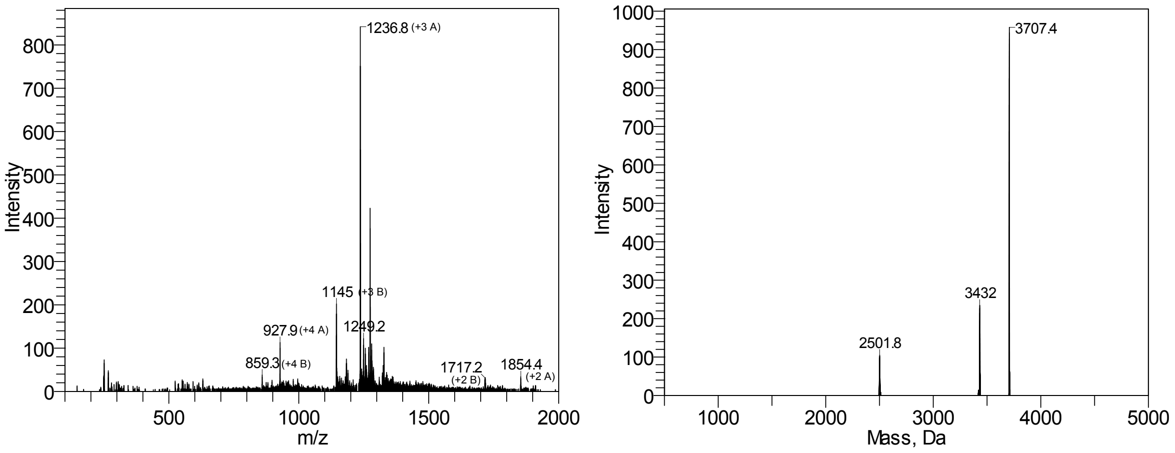

Figure S 27 - Raw (left) and deconvoluted (right) spectra of the reaction of **26** and benzyl hydroxylamine at 1 h.

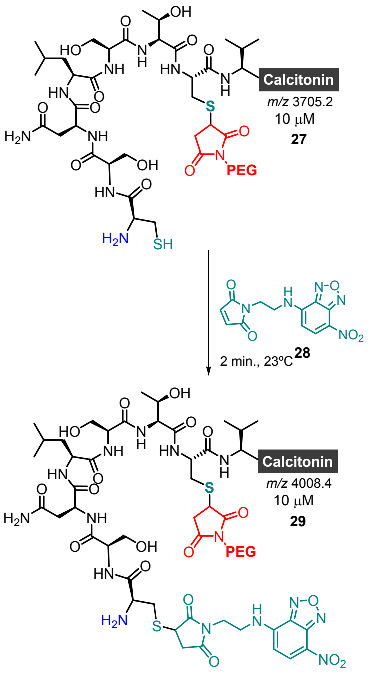

A solution of Calcitonin salmon (1mg/mL, 0.291 mM, 8.6 µL, 0.0025 µmol) was added to Acetate buffer 20 mM pH = 7 with 1% DMF (250 µL), followed by Tris(2-carboxyethyl)phosphine hydrochloride (TCEP) (3.5 mM in water, 1 mg/mL, 7.1 µL, 0.025 µmol) and the solution mixed for 30 min at 25 ºC. Then (2-formylphenyl)boronic acid (10 mM in DMF,2.5 µL, 0.025 µmol) was added and stirred for further 2 min. Then **25** (5 mM in H_2O, 0.5 µL, 0.0025 µmol) was added, stirred another 5 min and (benzyloxy)chloro-l5-azane (3.2 µL, 0.025 µmol) was added and the solution stirred for another 60 min, Finally 1-(2-((7-nitrobenzo[c][1,2,5]oxadiazol-4-yl)amino)ethyl)-1H-pyrrole-2,5-dione (**28**, 10mM in DMF,2.5 µL, 0.025 µmol) was added and the mass checked in Positive Mode of ESI-MS.

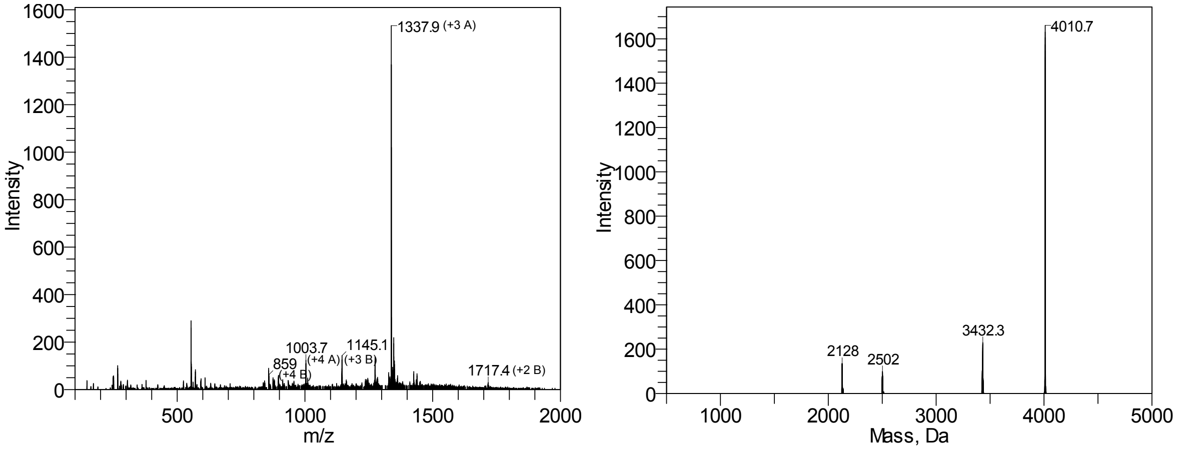

Figure S 28 - Raw (left) and deconvoluted (right) spectra of the reaction of **27** and **28** at 2 min.

3. Computational Studies

All calculations were performed using the GAUSSIAN 09 software package,[6] and the M06-2X functional, without symmetry constraints. That is a hybrid meta-GGA functional developed by Truhlar and Zhao,[7] and it was shown to perform very well for main-group kinetics, providing a good description of long range effects such as van der Waals interactions or π-π stacking.[8,9] The optimized geometries were obtained with a standard 6-31G+(d,p)[10] basis set, accounting for solvent effects (water) by means of the Polarisable Continuum Model (PCM) initially devised by Tomasi and coworkers[11] with radii and non-electrostatic terms of the SMD solvation model, developed by Truhler *et al*.[12]. Transition state optimizations were performed with the Synchronous Transit-Guided Quasi-Newton Method (STQN) developed by Schlegel *et al*,[13] following extensive searches of the Potential Energy Surface. Electronic energies were converted to free energy at 298.15 K and 1 atm by using zero point energy and thermal energy corrections based on structural and vibration frequency data calculated at the same level.

---

[6] Gaussian 09, Revision A.01, M. J. Frisch, G. W. Trucks, H. B. Schlegel, G. E. Scuseria, M. A. Robb, J. R. Cheeseman, G. Scalmani, V. Barone, B. Mennucci, G. A. Petersson, H. Nakatsuji, M. Caricato, X. Li, H. P. Hratchian, A. F. Izmaylov, J. Bloino, G. Zheng, J. L. Sonnenberg, M. Hada, M. Ehara, K. Toyota, R. Fukuda, J. Hasegawa, M. Ishida, T. Nakajima, Y. Honda, O. Kitao, H. Nakai, T. Vreven, J. A. Montgomery, Jr., J. E. Peralta, F. Ogliaro, M. Bearpark, J. J. Heyd, E. Brothers, K. N. Kudin, V. N. Staroverov, R. Kobayashi, J. Normand, K. Raghavachari, A. Rendell, J. C. Burant, S. S. Iyengar, J. Tomasi, M. Cossi, N. Rega, J. M. Millam, M. Klene, J. E. Knox, J. B. Cross, V. Bakken, C. Adamo, J. Jaramillo, R. Gomperts, R. E. Stratmann, O. Yazyev, A. J. Austin, R. Cammi, C. Pomelli, J. W. Ochterski, R. L. Martin, K. Morokuma, V. G. Zakrzewski, G. A. Voth, P. Salvador, J. J. Dannenberg, S. Dapprich, A. D. Daniels, Ö. Farkas, J. B. Foresman, J. V. Ortiz, J. Cioslowski, and D. J. Fox, Gaussian, Inc., Wallingford CT, 2009.

[7] Y. Zhao, D. G. Truhlar, *Theor. Chem. Acc.*, 2008, **120**, 215.

[8] Y. Zhao, D. G. Truhlar, *Acc, Chem. Res.*, 2008, **41**, 157.

[9] Y. Zhao, D. G. Truhlar, *Chem. Phys. Lett.*, 2011, **502**, 1.

[10] a) R. Ditchfield, W. J. Hehre, J. A. Pople, *J. Chem. Phys.* **1971**, *54*, 724; b) W. J. Hehre, R. Ditchfield, J. A. Pople, *J. Chem. Phys.* **1972**, *56*, 2257; c) P. C. Hariharan, J. A. Pople, *Mol. Phys.* **1974**, *27*, 209; d) M. S. Gordon, *Chem. Phys. Lett.* **1980**, *76*, 163; e) P. C. Hariharan, J. A. Pople, *Theor. Chim. Acta* **1973**, *28*, 213.

[11] a) M. T. Cancès, B. Mennucci, J. Tomasi, *J. Chem. Phys.* **1997**, *107*, 3032. b) M. Cossi, V. Barone, B. Mennucci, J. Tomasi, *Chem. Phys. Lett.* **1998**, *286*, 253. c) B. Mennucci, J. Tomasi, *J. Chem. Phys.* **1997**, *106*, 5151. d) J. Tomasi, B. Mennucci, R. Cammi, *Chem. Rev.* **2005**, *105*, 2999.

[12] A. V. Marenich, C. J. Cramer, D. G. Truhlar, *J. Phys. Chem. B*, **2009**, *113*, 6378-6396.

[13] a) C. Peng, P. Y. Ayala, H. B. Schlegel, M. J. Frisch, *J. Comput. Chem.* **1996**, *17*, 49; b) C. Peng, H. B. Schlegel, *Isr. J. Chem*. **1993**, *33*, 449.

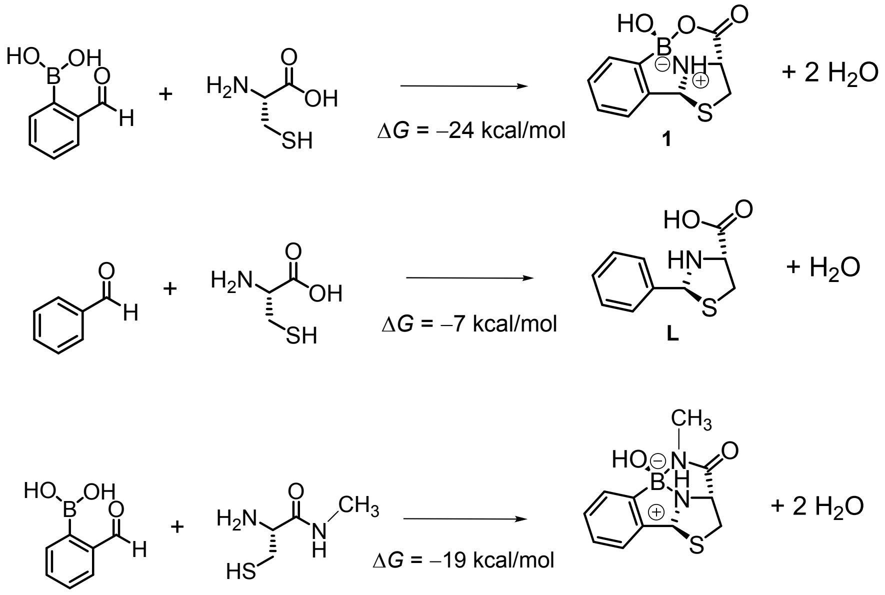

Figure S 29 - Free energy balance calculated for the reaction of Cys with 2FBBA (top), with benzaldehyde (middle) and between (*R*)-2-amino-3-mercapto-*N*-methylpropanamide with 2FBBA (bottom).

DFT calculated mechanism

The first part of the reaction corresponds to the formation of an imine intermediate resulting from the condensation of the amine group in Cys and the aldehyde carbonyl in the boronic acid. The model used for the calculations includes an explicit water molecule and the free energy profile obtained is represented in Figure S2 while a schematic illustration of the path is presented in Scheme S1.

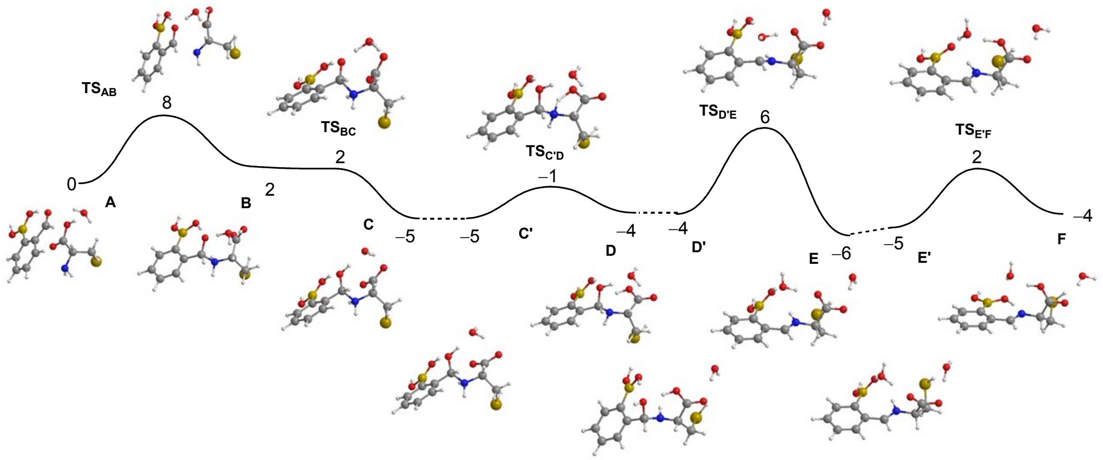

Figure S 30 - Free energy profile (kcal/mol) calculated for the formation of the imine intermediate from the reaction of 2FBBA and Cys.

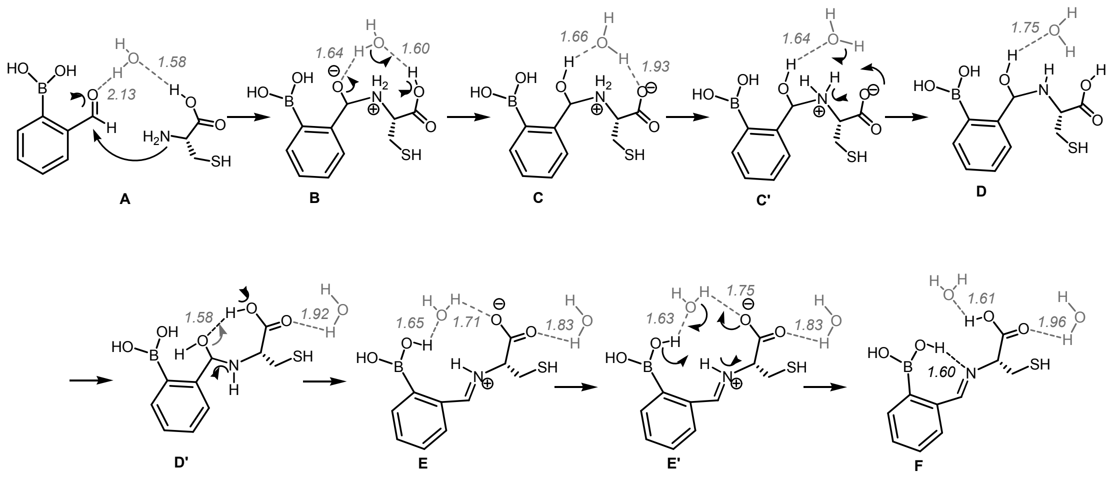

Scheme S1. Schematic representation of the mechanism of formation of the imine intermediate from the reaction of Cys and 2FBBA. H-bond distances in Å.

The reaction starts with the formation of a N–C bond between the Cys amine group and the carbonyl C-atom of 2FBBA, from **A** to **B**, in a process with a barrier of 8 kcal/mol and a slightly positive free energy balance (Δ*G* = 2 kcal/mol). In the next step, from **B** to **C**, there is protonation of the O-atom from the original carbonyl group while the carboxylic group of the amino acid is deprotonated. Thus, in **C** there is an ammonium ion (N^+) and a carboxylate group (COO^-). In this step the neighbour water molecule acts as proton shuttle, resulting in a barrierless and exergonic process (Δ*G* = –7 kcal/mol). From **C** to **C’**, there is a re-orientation of the water molecule and, then, in the following step (from **C’** to **D**) the N-atom of the ammonium ion in **C’** is deprotonated regenerating both the amine and the carboxylic groups, in **D**. This occurs with a small barrier ($\Delta G^‡$ = 4 kcal/mol) and is an almost thermoneutral process. (Δ*G* = 1 kcal/mol). After a re-orientation of the water molecule and the corresponding change in the H-bonds network (from **D** to **D’**), the reaction proceeds with protonation of the OH group involving the O-atom from the original aldehyde. The proton involved is originated the carboxylic group (COOH) and results in the loss of one water molecule, from **D’** to **E**. This step has the highest barrier so far ($\Delta G^‡$ = 10 kcal/mol) and is slightly exergonic (Δ*G* = –2 kcal/mol). In **E** there is an iminium ion and a carboxylate group, and two nearby water molecules: the one originally included in the model plus another, formed in the last step. A change in the H-bonds configuration transforms **E** into **E’** and, from here, the imine is produced in the last step of the mechanism (from **E’** to **F**) with deprotonation of the iminium ion and re-protonation of the carboxylate group. The proton transfer process is assisted by one neighbour water molecule and also by one of the OH groups attached to the boron atom, resulting in a barrier of $\Delta G^‡$ = 7 kcal/mol and a free energy balance of Δ*G* = 1 kcal/mol. The overall mechanism for the formation of the imine intermediate is rather facile with a barrier of 11 kcal/mol (measured between **C/C’** and $\mathbf{TS_{D'E}}$) and presents a favourable overall free energy balance of Δ*G* = –4 kcal/mol, as expected for a well known reversible reaction: formation of an imine from an amine and an aldehyde.

The second part of the mechanism goes from the imine to the final product, **1**, and the corresponding path is represented in Figure S3 (free energy profile) and Scheme S2 (schematic representation).

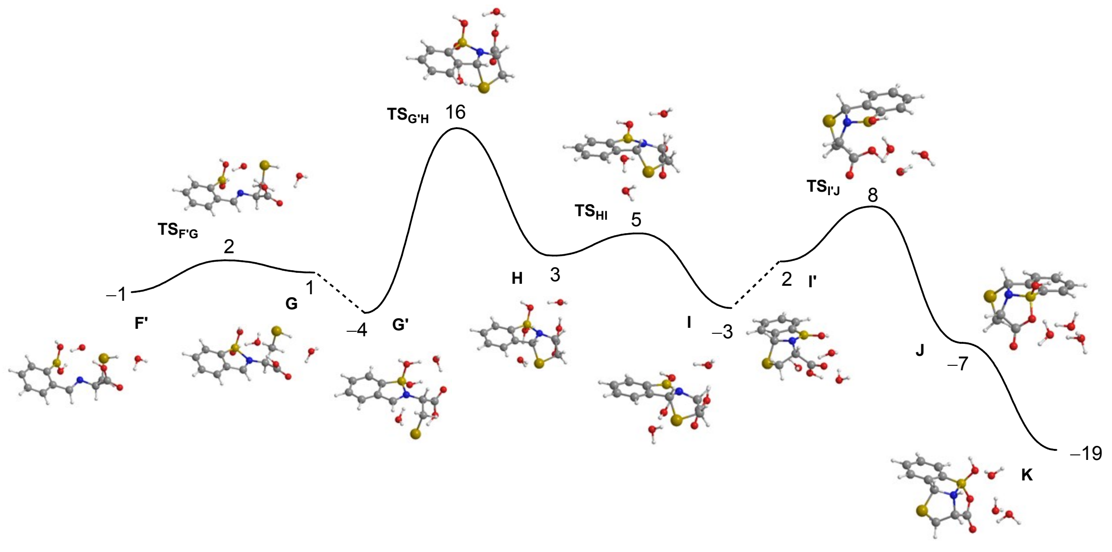

Figure S 31 - Free energy profile (kcal/mol) calculated for the formation of 1 from the imine intermediate. The free energy values refer to the initial reactants (A).

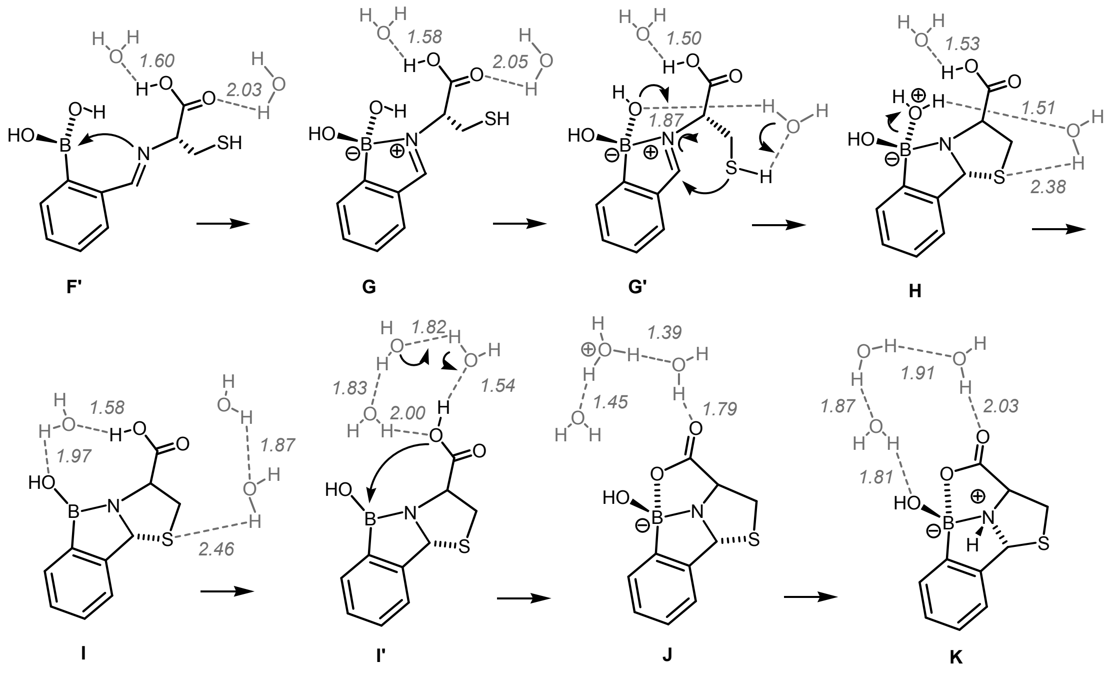

Scheme S2. Schematic representation of the mechanism of formation of **1** from the imine intermediate. H-bond distances in Å.

The second part of the reaction mechanism starts with **F'**, an imine similar to **F** but with a different conformation of the $B(OH)_2$ group. In the following step, from **F'** to **G** there is formation of a N–B bond and, thus, in **G** there is a new boron chelate 5-member ring (C_3NB), an iminium group ($C=N^+$) and a tetravalent, formally negative, B-atom. This step as a negligible barrier of 3 kcal/mol and is slightly endergonic (ΔG = 2 kcal/mol). From **G** to **G'**, there is a re-orientation of the $–CH(COOH)CH_2SH$ branch, attached to the N-atom, in a process that brings the S-atom and the C-atom of the iminium ion in the relative position necessary for the formation of the corresponding S–C bond, that will occur in the following step. Here, from **G'** to **H**, that bond is formed and, at the same time, there is transfer of a proton from the thiol to one of the OH groups bonded to the B-atom. Overall, in **H**, a second 5-member ring is formed, fused with the first one by the C–N bond, the nitrogen becomes an amine (tertiary), and there is one water molecule coordinated to the boron atom. The proton transfer from SH to OH is assisted by a neighbour water molecule, revealing once more the importance of the solvent molecules as shuttles in the proton exchange steps of the mechanism. Nevertheless, the barrier associated with this step ($\Delta G^‡$ = 20 kcal/mol) is the highest of the path and the free energy balance indicates an endergonic process (ΔG = 7 kcal/mol). The subsequent step, from **H** to **I**, corresponds to dissociation of the water molecule from the boron coordination in an easy process with a barrier of only 2 kcal/mol and a favourable balance of ΔG = –6 kcal/mol. A re-orientation of the three water molecules and of the COOH group brings **I** to **I'**. In the following step, from **I'** to **J**, there attack of the carboxylic group on the trivalent B-atom with simultaneous proton loss. Thus, in **J**, there is a third 5-member ring (BNC_2O) fused with the other two, forming another B-chelating ring. Also, in **J**, the proton lost by the carboxylic group is protonating a water molecule, forming an hydronium ion (H_3O^+) stabilized by H-bonds to the two surrounding water molecules. The barrier for this step is 6 kcal/mol and the process is exergonic by 9 kcal/mol. Proton exchange between the free hydronium ion and the amine N-atom forms the final product, **K**, that correspond to **1** with three water molecules. Of these molecules, one was included in the initial model while the other two are reaction by-products resulting from the two condensation processes along the path: formation of the imine from the initial reagents, and coordination of the carboxylic group to the boron atom. The final process, from **J** to **K**, is very favourable, from the thermodynamic point a view, with a free energy balance of ΔG = –12 kcal/mol, reflecting the basicity of the amine N-atom. This stability of the final product provides the reaction the driving force, being due in good part to multiple B-coordination and the corresponding chelate effect existing in the product.

The reaction is clearly favourable, from the thermodynamic point of view, with a balance of ΔG = –19 kcal/mol, corroborating the observed stability of the product. Moreover, the calculated overall energy barrier is $\Delta G^‡$ = 21 kcal/mol (measured from **C/C'** to $\mathbf{TS_{G'H}}$) being slightly high for the experimental observations of a fast room temperature reaction. That difference is most probably due to the simplicity of model employed, with a reduced number of explicit water molecules in a reaction were proton transfer process are of major importance. Importantly, the B-atom as a two-fold role in the reaction mechanism. On one hand, it provides activation of the imine group by means of N–B coordination, promoting the formation of the S–C bond and, on the other, it provides stabilization of the final product through multiple boron-coordination and the corresponding chelate effect.

Atomic coordinates of all optimized species

H_2O

H	0.309606	0.555609	0.407703
O	0.359183	-0.390182	0.219479
H	-0.355188	-0.554827	-0.408983

Cys

H	0.138635	0.782773	-2.205138
S	0.127099	-1.668401	-0.899771
O	1.000161	0.602546	1.624124
O	1.686201	2.042140	0.079665
N	-0.226284	1.523675	-1.606977
C	0.802372	1.178171	0.570169
C	-0.464658	1.008145	-0.264739
C	-0.993616	-0.422020	-0.172983
H	1.064486	-1.618017	0.062823
H	-1.202511	1.658262	0.222436
H	-1.920458	-0.488393	-0.746898
H	-1.212811	-0.686327	0.862273
H	1.324334	2.342365	-0.787685
H	-1.097150	1.849081	-2.015901

2FBBA

O	-1.478956	-0.990059	2.832160
O	1.100591	-1.114653	2.599077
B	1.283343	-0.624707	1.346176
C	-1.973553	-0.533419	1.806113
C	-1.271651	-0.135673	0.570348
C	-2.148300	0.303582	-0.432348
C	-1.677039	0.714425	-1.675460
C	-0.308291	0.689984	-1.914883
C	0.569410	0.257432	-0.917144
C	0.132169	-0.167158	0.343708
O	2.567906	-0.506717	0.874069
H	0.142568	-1.143668	2.832372
H	-3.065557	-0.395642	1.771543
H	-3.216011	0.319100	-0.229894
H	-2.369842	1.050057	-2.440083
H	0.084847	1.008836	-2.875467
H	1.632881	0.252101	-1.131979
H	3.218786	-0.793020	1.530292

Benzaldehyde

O	-0.470254	-1.001888	2.779659
H	1.496801	-0.563477	1.124431
C	-1.125691	-0.611177	1.824601
C	-0.542174	-0.178201	0.541930
C	-1.400923	0.271123	-0.466316
C	-0.876903	0.690556	-1.687302
C	0.502144	0.657612	-1.894616
C	1.361998	0.206280	-0.887262
C	0.842797	-0.210946	0.332206
H	-2.226467	-0.563199	1.890432
H	-2.473223	0.290510	-0.287507
H	-1.538407	1.042371	-2.472286
H	0.911955	0.985503	-2.845229
H	2.433348	0.183733	-1.059239

1 (N – *R*; C2 – *R*; B – *S*)

S	-1.113072	-1.729641	-0.530912
O	1.317410	-2.897562	2.132140
O	1.444437	-0.674192	1.925724
O	0.702334	1.293710	3.018387
H	0.472802	2.222830	2.895142
N	-0.905527	-0.184492	1.646210
H	-1.599829	0.277908	2.238104
B	0.590325	0.555673	1.812896
C	0.786097	-1.805662	2.040813
C	-0.726931	-1.597386	2.063559
H	-1.080329	-1.730276	3.089813
C	-1.462861	-2.494720	1.082950
H	-2.534602	-2.500507	1.289330
H	-1.075983	-3.514675	1.102081
C	-1.332284	-0.061418	0.206935
H	-2.387655	0.214159	0.171670
C	-0.420575	0.963991	-0.412673
C	-0.604821	1.518550	-1.677848
H	-1.462488	1.245782	-2.287699
C	0.334131	2.439631	-2.143024
H	0.210742	2.886555	-3.124831
C	1.427766	2.795641	-1.347661
H	2.149434	3.517136	-1.719250
C	1.589643	2.235857	-0.078436
H	2.438477	2.529601	0.534695
C	0.663359	1.306507	0.402887

Product of the benzaldehyde reaction (L)

S	-2.469867	0.121568	0.468914
O	-2.755390	-3.254561	-1.830726
O	-0.992164	-1.984288	-2.371059
H	-1.262745	-2.221070	-3.276662
H	0.075767	-1.178801	-0.741161
N	-0.312237	-1.426691	0.165287
H	0.818512	1.305074	2.530201
C	-1.815916	-2.558378	-1.495566
C	-1.480078	-2.295911	-0.035750
H	-1.309074	-3.288235	0.395884
C	-2.702042	-1.668946	0.663470
H	-2.697903	-1.927967	1.724590
H	-3.641811	-1.996439	0.218318
C	-0.685533	-0.191658	0.857758
H	-0.643545	-0.356788	1.937305
C	0.197033	0.973349	0.498664
C	0.319787	1.400676	-0.829038
H	-0.240766	0.899099	-1.615513
C	1.152169	2.470668	-1.148155
H	1.241439	2.795240	-2.180361
C	1.866439	3.127895	-0.143589
H	2.513571	3.962787	-0.394408
C	1.743688	2.710009	1.180297
H	2.295055	3.216293	1.966666
C	0.911313	1.635975	1.499034

Stereoisomers of 1 by order of energy

(N – *S*; C2 – *S*; B – *R*)

S	-2.812166	-0.633299	-0.728673
O	-0.389626	-2.729134	3.001881
O	1.093989	-1.962309	1.491298
N	-0.432674	-1.707674	-0.320481
H	-0.612570	-2.659003	-0.661678
B	1.132751	-1.390353	0.071203
C	-0.131336	-2.177061	1.952140
C	-1.093975	-1.509608	0.993235
H	-0.954134	-0.449278	1.245647
C	-2.568022	-1.712311	0.763501
H	-3.172340	-1.325315	1.583616
H	-2.830808	-2.747113	0.539989
C	-1.002061	-0.676649	-1.200776
H	-0.932466	-0.986914	-2.244093
C	-0.133632	0.533345	-0.899295
C	-0.412952	1.837466	-1.294085
H	-1.323655	2.072844	-1.839240
C	0.500474	2.843853	-0.975021
H	0.300081	3.868277	-1.274015
C	1.668852	2.539271	-0.272343
H	2.371494	3.330966	-0.029428
C	1.936483	1.224430	0.117123
H	2.850787	1.000975	0.661729
C	1.037552	0.200892	-0.188573
O	2.113729	-2.048157	-0.701416
H	2.148526	-2.995142	-0.508845

(N – *R*; C2 – *S*; B – *S*)

S	-2.791073	0.269231	1.197506

O	1.101882	-1.567468	0.674856
O	2.358725	0.262116	1.532551
H	2.691928	1.146548	1.338731
N	-0.223031	0.164642	1.505892
H	-0.284646	1.027144	2.056001
B	1.217650	-0.052765	0.757462
C	0.193009	-2.103524	1.476453
C	-0.602888	-1.035954	2.247166
H	-0.233015	-0.999895	3.273908
C	-2.153433	-1.162126	2.195839
H	-2.588684	-1.103448	3.192188
H	-2.451251	-2.100353	1.723338
C	-1.180175	0.218056	0.364527
H	-1.157207	-0.779011	-0.092329
C	-0.503121	1.115576	-0.632835
C	-1.052496	1.961140	-1.583401
H	-2.122728	2.142924	-1.626074
C	-0.183900	2.580088	-2.490396
H	-0.583864	3.257306	-3.238817
C	1.188060	2.335059	-2.437242
H	1.847727	2.823078	-3.148654
C	1.722163	1.475486	-1.468181
H	2.796417	1.308816	-1.430904
C	0.882523	0.848583	-0.550938
O	0.013529	-3.300548	1.591152

(N – *S*; C2 – *R*; B – *S*)

S	-2.636978	-0.752028	-0.346280
O	1.264564	-1.421404	1.965064
O	0.739996	0.717636	2.865169
H	0.766899	1.674995	2.749940
N	-0.553632	-0.524918	1.046968
H	-0.138352	-1.006984	0.237030
B	0.788099	0.029713	1.628285
C	0.246540	-2.278747	2.145116
C	-1.119079	-1.612636	1.787762
H	-1.601598	-1.261479	2.705001
C	-2.126499	-2.199220	0.776529
H	-3.020874	-2.590951	1.256817
H	-1.667144	-2.964342	0.149002
C	-1.397244	0.435577	0.398273
H	-1.906689	1.071918	1.125590
C	-0.275428	1.194417	-0.331385
C	-0.430759	2.047973	-1.413655
H	-1.410032	2.205277	-1.858019
C	0.696753	2.697262	-1.926423
H	0.594119	3.360170	-2.780115
C	1.948865	2.497972	-1.346780
H	2.816736	3.007695	-1.754901
C	2.097271	1.648990	-0.243517
H	3.081464	1.516334	0.198481
C	1.000785	0.971228	0.285194
O	0.388716	-3.415347	2.541554

A

O	-1.585900	1.339458	2.624304
H	-2.789210	-0.357459	3.074957
H	-0.656190	-1.526637	-2.082035
S	-1.016202	-4.107147	-0.850873
O	0.805970	-1.101432	0.001888
O	-0.383501	-1.442123	1.828615
O	1.014279	1.275148	2.412947
N	-1.356789	-0.932373	-1.641247
B	1.186462	1.685756	1.129908
C	-0.286817	-1.271550	0.518989
C	-1.574509	-1.387380	-0.281292
C	-2.117131	-2.826735	-0.154711
C	-2.078059	1.758257	1.579527
C	-1.371189	2.177702	0.355702
C	-2.247855	2.618789	-0.647619
C	-1.775170	3.041551	-1.885938
C	-0.405980	3.014922	-2.124740
C	0.470217	2.571223	-1.130602
C	0.032630	2.143604	0.129406

O	2.465737	1.748343	0.633402
H	-0.099542	-4.089529	0.133537
H	0.060009	1.242694	2.652193
H	-2.319643	-0.736118	0.195299
H	-3.040810	-2.889451	-0.735339
H	-2.353498	-3.078475	0.881345
H	-3.175161	1.832280	1.515905
H	-3.316407	2.626708	-0.448606
H	-2.466881	3.383552	-2.648713
H	-0.011722	3.337959	-3.083498
H	1.533244	2.559499	-1.347341
H	3.119073	1.469582	1.290468
O	-2.745461	-1.307909	2.880872
H	-1.333110	-1.440285	2.176008
H	-3.460166	-1.479506	2.250452
H	-2.221909	-1.060315	-2.161864

TS_{AB}

O	-1.509712	0.761076	1.822617
H	-2.579245	-0.244108	2.964611
H	-0.762874	-0.737211	-1.018910
S	-0.981487	-3.736140	-1.513505
O	0.526852	-1.799278	0.732322
O	-0.722462	-2.215237	2.509884
O	1.026029	1.084634	1.922077
N	-1.666325	-0.679923	-0.549139
B	1.270887	1.771800	0.777423
C	-0.581521	-1.897783	1.229427
C	-1.840898	-1.776234	0.384360
C	-2.158195	-3.143495	-0.254312
C	-1.923074	1.101661	0.691652
C	-1.219514	2.042489	-0.230432
C	-2.064458	2.634093	-1.176154
C	-1.577640	3.544189	-2.110354
C	-0.222306	3.858806	-2.106128
C	0.623161	3.260846	-1.171419
C	0.163695	2.346884	-0.209453
O	2.577194	2.030599	0.432328
H	0.067885	-3.917659	-0.694629
H	0.047123	0.930872	2.028196
H	-2.692064	-1.544005	1.032855
H	-3.111995	-3.048911	-0.779835
H	-2.281321	-3.895282	0.529082
H	-2.995471	0.994268	0.483505
H	-3.120149	2.374161	-1.177760
H	-2.250512	3.996599	-2.832012
H	0.179862	4.564324	-2.826746
H	1.678462	3.514826	-1.187725
H	3.194664	1.665796	1.081747
O	-2.848663	-1.016823	3.504128
H	-1.625299	-1.972555	2.863935
H	-3.731023	-1.260097	3.192741
H	-2.402797	-0.680579	-1.253574

B

O	-1.305286	0.607132	1.786777
H	-2.426421	-0.012199	2.805845
H	-0.390641	-0.643331	-0.447494
S	-0.723521	-3.487076	-1.534297
O	0.405479	-2.208334	1.238131
O	-1.182981	-2.372053	2.778645
O	1.151130	0.831341	1.607672
N	-1.403454	-0.601402	-0.262377
B	1.331968	1.642113	0.540487
C	-0.766593	-2.109661	1.553048
C	-1.828982	-1.799605	0.501889
C	-2.071519	-3.016395	-0.399943
C	-1.748792	0.720801	0.519533
C	-1.203875	1.869593	-0.321768
C	-2.133647	2.516473	-1.140663
C	-1.756270	3.571875	-1.968882
C	-0.428167	3.986823	-1.981950
C	0.502465	3.337274	-1.172072
C	0.152868	2.272709	-0.324921

O	2.611514	1.981984	0.152658
H	0.168098	-3.847639	-0.597666
H	0.137584	0.696748	1.780250
H	-2.781625	-1.554541	0.977672
H	-2.925667	-2.793049	-1.044372
H	-2.347006	-3.862864	0.232494
H	-2.847398	0.726864	0.459854
H	-3.170542	2.190017	-1.127995
H	-2.496215	4.063442	-2.593115
H	-0.116009	4.810201	-2.617230
H	1.536354	3.668764	-1.194936
H	3.271843	1.581635	0.735265
O	-2.973429	-0.625549	3.383526
H	-2.035006	-1.872507	3.007100
H	-3.852133	-0.664257	2.982133
H	-1.879316	-0.572725	-1.172996

TS$_{BC}$

O	-1.362077	0.649815	1.788096
H	-2.219443	0.058013	2.668439
H	-0.331042	-0.671405	-0.313141
S	-0.727219	-3.462575	-1.564667
O	0.338531	-2.346699	1.289579
O	-1.316857	-2.410053	2.785797
O	1.135918	0.826552	1.617152
N	-1.356954	-0.623712	-0.224668
B	1.309006	1.639038	0.545284
C	-0.842530	-2.198132	1.598071
C	-1.849658	-1.826468	0.500575
C	-2.093810	-3.007757	-0.445121
C	-1.769075	0.697923	0.493723
C	-1.224338	1.849688	-0.338340
C	-2.150722	2.492671	-1.163415
C	-1.773119	3.556920	-1.980130
C	-0.448773	3.983045	-1.975338
C	0.478807	3.335668	-1.160151
C	0.129081	2.263203	-0.323074
O	2.586556	1.976535	0.158159
H	0.165427	-3.782916	-0.614753
H	0.145854	0.700641	1.811793
H	-2.815329	-1.563022	0.939048
H	-2.928803	-2.752214	-1.102834
H	-2.395974	-3.869281	0.154090
H	-2.863618	0.675025	0.404368
H	-3.184677	2.157520	-1.164159
H	-2.510449	4.046311	-2.609061
H	-0.136787	4.813422	-2.601442
H	1.510004	3.675651	-1.170402
H	3.248777	1.580267	0.741563
O	-2.751038	-0.586566	3.363454
H	-2.134862	-1.615495	3.092044
H	-3.676692	-0.647377	3.085683
H	-1.737810	-0.596635	-1.178920

C

O	-1.324019	0.704351	1.796416
H	-1.970093	0.237338	2.398538
H	-0.249095	-0.713862	-0.097992
S	-0.673804	-3.517730	-1.468931
O	0.313718	-2.062828	1.465827
O	-1.448064	-2.924336	2.576069
O	1.315413	0.911588	1.590345
N	-1.278613	-0.638849	-0.164939
B	1.378826	1.677185	0.466277
C	-0.904087	-2.307583	1.621831
C	-1.857175	-1.844843	0.500392
C	-2.119965	-2.969601	-0.499936
C	-1.735447	0.672975	0.471892
C	-1.205803	1.838087	-0.339160
C	-2.158235	2.477688	-1.137166
C	-1.814963	3.558651	-1.946191
C	-0.498582	4.005255	-1.960363
C	0.454594	3.359507	-1.174663
C	0.142970	2.268421	-0.345318

O	2.621759	1.995454	-0.022280
H	0.075304	-3.931202	-0.431140
H	0.399310	0.744813	1.889752
H	-2.818090	-1.553065	0.933481
H	-2.853546	-2.633188	-1.237548
H	-2.551190	-3.811982	0.044124
H	-2.825847	0.638283	0.404002
H	-3.186595	2.127758	-1.123466
H	-2.573378	4.044073	-2.552311
H	-0.210432	4.849816	-2.578602
H	1.479232	3.716348	-1.202159
H	3.330008	1.632751	0.528469
O	-2.918352	-0.630886	3.445337
H	-2.571015	-1.535637	3.317956
H	-3.827453	-0.655561	3.115783
H	-1.531485	-0.606587	-1.160524

C’

O	-1.372181	0.780603	1.796111
H	-1.965598	0.248011	2.396855
H	-0.233498	-0.708444	0.069892
S	-0.677503	-3.539789	-1.452226
O	0.144955	-1.951978	1.693467
O	-1.529075	-3.358821	2.236240
O	1.268182	0.996161	1.647497
N	-1.247331	-0.627362	-0.121614
B	1.351782	1.737426	0.508554
C	-1.010093	-2.428255	1.574604
C	-1.898842	-1.846015	0.452653
C	-2.172914	-2.887676	-0.628737
C	-1.748578	0.691918	0.464676
C	-1.214653	1.843783	-0.363199
C	-2.155081	2.450042	-1.200359
C	-1.806652	3.517491	-2.025033
C	-0.497294	3.984388	-2.014841
C	0.444839	3.370674	-1.191001
C	0.127902	2.293762	-0.345459
O	2.602168	2.061974	0.044223
H	-0.148687	-4.135389	-0.367248
H	0.344006	0.830294	1.924697
H	-2.859670	-1.539835	0.877718
H	-2.783319	-2.449288	-1.422617
H	-2.736639	-3.706521	-0.180329
H	-2.835839	0.637185	0.366862
H	-3.178146	2.084616	-1.205251
H	-2.555559	3.976700	-2.662634
H	-0.205816	4.819555	-2.644202
H	1.464562	3.742168	-1.200892
H	3.300679	1.724567	0.622821
O	-2.797343	-0.609213	3.525202
H	-2.160052	-1.190933	3.964930
H	-3.430095	-1.209140	3.104706
H	-1.382315	-0.585060	-1.138763

TS$_{C'D}$

O	-1.584346	1.014332	1.840753
H	-2.049659	0.367480	2.429464
H	-0.204070	-0.894995	0.947252
S	-0.626404	-3.265922	-1.591916
O	0.063082	-1.794527	1.776654
O	-1.329422	-3.549951	2.051930
O	1.093572	1.091184	1.710800
N	-1.124902	-0.565727	0.135227
B	1.247193	1.624917	0.466059
C	-1.003732	-2.480056	1.536698
C	-1.877316	-1.798667	0.462637
C	-2.138908	-2.710616	-0.729015
C	-1.804910	0.716843	0.482650
C	-1.284063	1.839258	-0.389891
C	-2.217473	2.475068	-1.209840
C	-1.834535	3.508176	-2.064220
C	-0.500687	3.901043	-2.108312
C	0.434083	3.256879	-1.298241
C	0.075095	2.223864	-0.418214

O	2.510759	1.698845	-0.062271
H	-0.155180	-4.036866	-0.595054
H	0.159303	1.093314	2.007526
H	-2.840426	-1.538665	0.916134
H	-2.733984	-2.179592	-1.475531
H	-2.709319	-3.578011	-0.394039
H	-2.876288	0.583556	0.302101
H	-3.256448	2.158615	-1.180339
H	-2.575571	3.995780	-2.690048
H	-0.185966	4.702864	-2.769218
H	1.473083	3.570302	-1.343937
H	3.183283	1.365012	0.548870
O	-2.708548	-0.707847	3.595040
H	-1.991108	-1.242472	3.964459
H	-3.312450	-1.345170	3.188311
H	-0.814936	-0.558646	-0.839176

D

O	-1.610566	1.004777	1.793977
H	-2.080316	0.356414	2.369137
H	0.223160	-1.030303	1.380824
S	-0.485955	-3.277572	-1.529525
O	0.101567	-1.835552	1.936982
O	-1.405428	-3.453568	2.098551
O	1.038732	0.989743	1.630478
N	-1.160894	-0.510187	0.029576
B	1.190099	1.510931	0.376649
C	-1.029487	-2.423382	1.568463
C	-1.812579	-1.744911	0.442685
C	-2.025042	-2.738514	-0.701581
C	-1.845930	0.720396	0.420227
C	-1.331812	1.872956	-0.415575
C	-2.257345	2.575913	-1.187449
C	-1.851134	3.627336	-2.009031
C	-0.503779	3.971405	-2.068178
C	0.422818	3.261777	-1.304183
C	0.039150	2.209126	-0.458897
O	2.436289	1.486378	-0.196627
H	-0.018117	-4.025540	-0.513803
H	0.102819	1.049049	1.929398
H	-2.807166	-1.536713	0.864744
H	-2.628652	-2.259393	-1.475881
H	-2.565578	-3.612852	-0.337429
H	-2.930836	0.620447	0.277337
H	-3.306494	2.294902	-1.148309
H	-2.584166	4.167536	-2.600386
H	-0.173428	4.785593	-2.706102
H	1.472680	3.536310	-1.361180
H	3.098617	1.085417	0.384234
O	-2.796932	-0.717404	3.556376
H	-2.089673	-1.151811	4.053708
H	-3.310875	-1.439045	3.167976
H	-0.962444	-0.508056	-0.967885

D’

O	-1.076764	0.317299	0.851908
H	-1.655107	0.960996	1.290215
H	-0.852763	-1.133474	1.439289
S	0.048103	-3.890533	-2.223836
O	-0.694862	-2.096587	1.673614
O	-1.026897	-4.128301	0.897715
O	1.597748	0.462009	0.489619
N	-0.993581	-1.098490	-1.047247
B	1.632233	1.053475	-0.739581
C	-1.085225	-2.919041	0.718629
C	-1.632226	-2.323348	-0.589487
C	-1.625846	-3.390985	-1.682561
C	-1.462158	0.152216	-0.544922
C	-0.970057	1.325333	-1.373441
C	-1.949657	2.025265	-2.081988
C	-1.617365	3.096732	-2.909683
C	-0.284499	3.472694	-3.038922
C	0.696002	2.771646	-2.337904
C	0.389678	1.697005	-1.486361

O	2.842924	1.144333	-1.378048
H	0.372011	-4.576408	-1.112914
H	0.703227	0.468394	0.878603
H	0.020742	-1.172361	-1.089545
H	-2.683266	-2.085832	-0.372315
H	-2.112465	-2.984651	-2.570396
H	-2.178083	-4.273818	-1.361432
H	-2.554659	0.132460	-0.566217
H	-2.990173	1.726235	-1.986742
H	-2.397496	3.628924	-3.445304
H	-0.006400	4.305468	-3.677509
H	1.733688	3.073493	-2.447309
H	3.563272	0.774405	-0.847947
O	1.642393	-5.085199	1.444757
H	1.911798	-5.408689	0.575696
H	0.721727	-4.797168	1.329566

$TS_{D'E}$

O	-0.979089	0.355846	1.011513
H	-1.676087	0.805891	1.515521
H	-0.915318	-0.655596	1.313748
S	0.534940	-3.755698	-1.984050
O	-0.742625	-2.035690	1.533416
O	-1.252551	-4.109814	0.905756
O	1.662690	0.319607	0.230412
N	-0.985184	-1.114447	-1.100801
B	1.603057	0.847467	-1.025862
C	-1.147945	-2.885630	0.680272
C	-1.570437	-2.395081	-0.724115
C	-1.257286	-3.439871	-1.791305
C	-1.498445	0.061858	-0.752936
C	-1.014496	1.308014	-1.407275
C	-2.022433	2.142812	-1.896210
C	-1.703857	3.295989	-2.611580
C	-0.368826	3.612852	-2.838954
C	0.635441	2.780215	-2.342778
C	0.347518	1.620751	-1.609230
O	2.704523	0.762214	-1.832047
H	0.710132	-4.383554	-0.805999
H	0.853199	0.473391	0.748592
H	-0.030965	-1.153167	-1.451854
H	-2.658706	-2.258965	-0.685750
H	-1.605321	-3.089889	-2.764952
H	-1.767321	-4.373018	-1.558233
H	-2.559140	0.037091	-0.516839
H	-3.062802	1.880960	-1.725211
H	-2.495471	3.935619	-2.988477
H	-0.104775	4.506073	-3.396519
H	1.673986	3.046045	-2.518866
H	3.457370	0.345015	-1.388311
O	1.283641	-4.953732	1.816242
H	1.573572	-5.537045	1.103274
H	0.375010	-4.693516	1.573407

E

O	-0.309020	0.542256	1.404729
H	-0.013363	0.757968	2.299452
H	-0.621941	-0.392950	1.440338
S	1.004986	-3.709765	-1.421389
O	-0.975942	-2.063689	1.426956
O	-1.693968	-4.092795	0.811596
O	1.678117	0.144771	-0.283336
N	-0.888814	-1.155065	-1.124143
B	1.524014	0.821782	-1.452263
C	-1.368651	-2.902075	0.579556
C	-1.462983	-2.482056	-0.904454
C	-0.769071	-3.500710	-1.807329
C	-1.587571	-0.106329	-1.398595
C	-1.126101	1.237062	-1.711684
C	-2.175383	2.124738	-2.005716
C	-1.915846	3.438357	-2.380259
C	-0.596363	3.865020	-2.481821
C	0.451565	2.981241	-2.202855
C	0.224837	1.663354	-1.801637

O	2.522456	0.821163	-2.384454
H	0.829737	-4.327318	-0.237010
H	0.979843	0.344194	0.400466
H	0.130697	-1.099393	-1.030996
H	-2.522829	-2.434107	-1.172865
H	-0.819895	-3.174434	-2.847905
H	-1.280419	-4.458628	-1.718541
H	-2.664298	-0.266340	-1.427677
H	-3.201433	1.773754	-1.944937
H	-2.735777	4.114008	-2.598602
H	-0.374001	4.884572	-2.781584
H	1.474003	3.336691	-2.293848
H	3.310355	0.347821	-2.078825
O	0.431782	-5.126208	2.324721
H	0.862051	-5.730531	1.706390
H	-0.354776	-4.793864	1.848519

E’

O	0.154780	0.876502	2.067941
S	1.077919	-2.957353	-0.042183
O	-0.566969	-3.757678	3.653291
H	0.641306	0.656273	2.874150
O	-1.784987	-1.039489	1.824922
H	0.553770	-3.318509	1.144429
H	0.196475	-4.239005	3.310237
O	-2.100691	-3.210978	1.366386
H	-0.542316	0.188338	1.980770
O	1.739788	0.784348	-0.030355
H	1.161553	0.831013	0.782436
N	-0.914082	-0.488645	-0.685216
H	0.107241	-0.404413	-0.685851
B	1.390536	1.619122	-1.043368
C	-1.804928	-2.030276	1.056646
C	-1.458501	-1.825333	-0.442391
H	-2.400102	-1.901305	-0.996831
C	-0.504707	-2.897978	-0.952633
H	-0.248644	-2.708999	-1.997526
H	-1.001183	-3.865455	-0.891413
C	-1.662889	0.562036	-0.718303
H	-2.731546	0.367475	-0.634130
C	-1.277946	1.953958	-0.880685
C	-2.378105	2.829370	-0.893291
H	-3.378284	2.425351	-0.766509
C	-2.201881	4.196467	-1.072359
H	-3.059617	4.860271	-1.073194
C	-0.916497	4.693528	-1.259771
H	-0.758550	5.757152	-1.410291
C	0.179405	3.824422	-1.264825
H	1.172326	4.236513	-1.421321
C	0.038851	2.449516	-1.064240
O	2.230625	1.799060	-2.106156
H	3.061275	1.310688	-2.007032
H	-1.142422	-3.605488	2.877765

$TS_{E'F}$

O	-0.049641	0.949917	2.066686
S	0.930479	-3.013582	-0.342618
O	0.207491	-4.430265	2.935546
H	0.426117	1.087901	2.898285
O	-1.124151	-1.197557	2.306740
H	0.639963	-3.614791	0.828668
H	0.118120	-5.391803	2.937770
O	-2.008992	-3.123784	1.689856
H	-0.646965	-0.201969	2.107464
O	1.466212	0.724701	0.128935
H	0.660253	0.918597	1.282769
N	-0.960256	-0.332567	-0.354101
H	0.073801	-0.177753	-0.238019
B	1.386924	1.665350	-0.811155
C	-1.556328	-2.013335	1.404718
C	-1.526113	-1.650116	-0.086486
H	-2.570833	-1.638158	-0.413797
C	-0.793232	-2.737614	-0.875731
H	-0.752811	-2.450979	-1.928275

H	-1.356713	-3.667213	-0.795544
C	-1.697540	0.676277	-0.680907
H	-2.767157	0.477992	-0.738836
C	-1.282763	2.035466	-0.989459
C	-2.372519	2.893205	-1.230088
H	-3.383753	2.502983	-1.156840
C	-2.172057	4.228761	-1.556196
H	-3.020383	4.881933	-1.730729
C	-0.870131	4.707645	-1.665921
H	-0.691407	5.746601	-1.926742
C	0.214241	3.852433	-1.448662
H	1.219423	4.250089	-1.551661
C	0.053136	2.508554	-1.099268
O	2.459971	2.032674	-1.614812
H	3.250714	1.527581	-1.378204
H	-0.672600	-4.092678	2.697711

F

O	0.352830	0.923472	1.989162
S	0.946007	-3.068034	-0.078521
O	-0.172928	-4.432268	3.080974
H	0.756219	0.967622	2.867791
O	-1.172247	-1.140015	2.239044
H	0.523862	-3.678235	1.046522
H	-0.307632	-5.388323	3.064651
O	-2.233684	-2.982317	1.679866
H	-0.623328	-0.350169	1.929484
O	1.550390	0.447052	-0.665579
H	1.090068	0.806992	1.368597
N	-0.900549	-0.368950	-0.427078
H	0.659484	-0.015285	-0.527997
B	1.390545	1.729292	-1.086809
C	-1.658973	-1.957606	1.334220
C	-1.507996	-1.660458	-0.156092
H	-2.530347	-1.703099	-0.557098
C	-0.708220	-2.794978	-0.806166
H	-0.545785	-2.551062	-1.857959
H	-1.282185	-3.721055	-0.756151
C	-1.689497	0.602799	-0.685615
H	-2.771374	0.426648	-0.690132
C	-1.307704	1.995100	-0.991760
C	-2.417282	2.846303	-1.113266
H	-3.413808	2.434181	-0.980766
C	-2.271501	4.201293	-1.393404
H	-3.146698	4.837972	-1.474120
C	-0.993845	4.719966	-1.567740
H	-0.852356	5.773606	-1.788406
C	0.113866	3.877541	-1.461792
H	1.101461	4.302520	-1.607933
C	0.005781	2.509220	-1.173752
O	2.509521	2.445488	-1.440734
H	3.312872	1.913681	-1.351612
H	-1.014968	-4.050401	2.783271

F’

O	0.382608	0.465173	2.194161
S	1.224858	-2.372583	0.388746
O	-0.368819	-4.282642	2.896139
H	0.886431	0.164537	1.416909
O	-1.496256	-1.308893	2.223836
H	0.764178	-3.126095	1.408933
H	-0.200400	-5.193267	2.621229
O	-2.520676	-3.039449	1.332261
H	-0.835051	-0.559956	2.059466
O	2.092171	1.309124	-0.158426
H	0.060619	1.344335	1.943271
N	-1.032018	-0.209410	-0.300175
H	1.968691	1.960072	0.546330
B	1.105881	1.343237	-1.112758
C	-1.865464	-2.016310	1.177407
C	-1.433923	-1.598198	-0.223382
H	-2.290305	-1.823176	-0.874027
C	-0.269505	-2.504825	-0.656570
H	0.034904	-2.215029	-1.664530

H	-0.601512	-3.544494	-0.681989
C	-1.811038	0.598016	-0.904036
H	-2.784038	0.283586	-1.303995
C	-1.433541	2.014551	-1.068130
C	-2.445930	2.973269	-1.169570
H	-3.484996	2.653934	-1.178359
C	-2.124285	4.327933	-1.232761
H	-2.912476	5.071163	-1.301626
C	-0.787270	4.720934	-1.203146
H	-0.528814	5.774534	-1.249623
C	0.222892	3.758620	-1.130801
H	1.261775	4.080100	-1.134389
C	-0.075889	2.392310	-1.074346
O	1.236064	0.532982	-2.209810
H	2.015808	-0.038711	-2.158508
H	-1.244173	-4.068374	2.535864

$TS_{F'G}$

O	0.089522	0.558365	2.204025
S	1.374155	-2.203296	0.889780
O	-0.682288	-4.391651	2.741637
H	0.770850	0.231599	1.587324
O	-1.739007	-1.222926	1.973461
H	0.715591	-2.999284	1.757024
H	-0.437217	-5.251981	2.377003
O	-2.491394	-2.997870	0.909151
H	-1.057658	-0.466035	1.951496
O	1.829358	0.946646	-0.240285
H	-0.158457	1.433581	1.868252
N	-0.822211	-0.140629	-0.521123
H	1.803951	1.664883	0.405408
B	0.822006	1.061155	-1.201886
C	-1.839795	-1.961917	0.889831
C	-1.091733	-1.566005	-0.383709
H	-1.738544	-1.886876	-1.208983
C	0.194233	-2.392861	-0.493387
H	0.723946	-2.078895	-1.395142
H	-0.061047	-3.449024	-0.598261
C	-1.807222	0.673342	-0.510357
H	-2.842034	0.362069	-0.330838
C	-1.498096	2.077896	-0.793731
C	-2.448115	3.096656	-0.726408
H	-3.473040	2.870799	-0.443617
C	-2.058888	4.401697	-1.027342
H	-2.781884	5.210000	-0.980226
C	-0.736749	4.665634	-1.388512
H	-0.437608	5.682856	-1.623995
C	0.205363	3.631746	-1.453640
H	1.229061	3.861057	-1.739913
C	-0.164013	2.318175	-1.163232
O	1.068880	0.459538	-2.434350
H	1.782376	-0.191197	-2.375251
H	-1.452292	-4.108751	2.222892

G

O	-0.338498	0.673503	2.435933
S	1.380774	-1.921497	1.237185
O	-0.708686	-4.375132	2.606504
H	0.543990	0.331163	2.216863
O	-1.889773	-1.285011	1.801924
H	0.712754	-2.829503	1.977298
H	-0.254567	-5.150139	2.251182
O	-2.395021	-3.042230	0.577046
H	-1.285997	-0.476816	1.905401
O	1.729801	0.625508	-0.585106
H	-0.410738	1.521642	1.973276
N	-0.782044	-0.058073	-0.596977
H	1.696750	1.064601	0.273621
B	0.529471	0.818820	-1.340603
C	-1.806975	-1.976489	0.684923
C	-0.899804	-1.505104	-0.453104
H	-1.350390	-1.896418	-1.372247
C	0.455821	-2.213115	-0.311151
H	1.110151	-1.902678	-1.124871

H	0.285747	-3.286647	-0.414261
C	-1.779802	0.729019	-0.396181
H	-2.746409	0.384742	-0.026503
C	-1.505014	2.126147	-0.719379
C	-2.375615	3.199717	-0.540293
H	-3.369268	3.051290	-0.126456
C	-1.926805	4.468370	-0.908209
H	-2.574083	5.330881	-0.784984
C	-0.642564	4.630201	-1.437442
H	-0.307201	5.623578	-1.722025
C	0.213285	3.533804	-1.609550
H	1.204911	3.689902	-2.027916
C	-0.212194	2.258653	-1.249226
O	0.660480	0.336107	-2.685064
H	1.314591	-0.372664	-2.741377
H	-1.407578	-4.176137	1.964365

G’

O	2.888942	-2.374622	1.273315
S	-3.102745	-2.558767	-0.100240
O	-0.692430	-0.537186	-3.165005
H	2.661968	-1.416596	1.156049
O	1.427786	-3.354130	-0.534454
H	-2.575563	-3.337167	-1.055342
H	0.169787	-0.451459	-2.714835
O	-0.418934	-3.022363	-1.691686
H	1.968688	-3.001110	0.268933
O	2.080908	0.126263	0.716763
H	2.637772	-2.601569	2.180020
N	-0.288409	-0.563014	0.075045
H	1.835733	0.658906	1.484461
B	1.136587	0.248068	-0.373074
C	0.236756	-2.818983	-0.682067
C	-0.313127	-1.963023	0.475998
H	0.385510	-2.043869	1.316338
C	-1.641828	-2.516310	0.999845
H	-1.947867	-1.913465	1.859523
H	-1.447246	-3.525021	1.370229
C	-1.289220	0.243239	0.097128
H	-2.292390	-0.048804	0.395196
C	-0.937080	1.604890	-0.304394
C	-1.808936	2.686909	-0.401140
H	-2.864546	2.578982	-0.167503
C	-1.280050	3.912073	-0.810445
H	-1.925966	4.779970	-0.897589
C	0.080993	4.024362	-1.107515
H	0.477978	4.984408	-1.425140
C	0.938617	2.920161	-1.002552
H	1.993430	3.037425	-1.239352
C	0.433959	1.689237	-0.596491
O	1.653679	-0.338639	-1.580898
H	2.223787	-1.100827	-1.407237
H	-1.071940	-1.327967	-2.753183

$TS_{G'H}$

O	2.671069	-1.889481	1.941424
S	-2.609359	-0.980102	-0.426685
O	-0.976945	-0.993792	-2.873319
H	2.575234	-1.020004	1.456907
O	1.819523	-3.117009	-0.095027
H	-1.846041	-0.987654	-1.668436
H	-0.042281	-0.889399	-2.520478
O	0.091376	-3.452069	-1.420999
H	2.104812	-2.676375	0.788671
O	2.285256	0.135126	0.352911
H	2.053539	-1.862051	2.685589
N	-0.041508	-0.700474	0.393638
H	3.026029	0.490255	-0.155119
B	1.062919	0.050314	-0.474780
C	0.548189	-2.953125	-0.405783
C	-0.302780	-2.123490	0.567062
H	-0.057003	-2.459978	1.583558
C	-1.816748	-2.416824	0.351069
H	-2.318372	-2.561285	1.308949

H	-1.970106	-3.299524	-0.268664
C	-1.157904	0.080550	0.601449
H	-1.661989	0.023626	1.570407
C	-0.933521	1.429491	0.013278
C	-1.789668	2.525333	0.092832
H	-2.734558	2.461651	0.627211
C	-1.402228	3.707342	-0.539848
H	-2.047678	4.579588	-0.501633
C	-0.183749	3.771949	-1.224848
H	0.105554	4.698532	-1.713045
C	0.662742	2.660109	-1.280560
H	1.611741	2.735677	-1.808048
C	0.292152	1.466337	-0.658873
O	1.361952	-0.568839	-1.779108
H	2.000865	-1.291760	-1.705419
H	-1.020410	-1.892644	-3.233582

H

O	2.851554	-2.208076	1.610611
S	-2.613436	-0.980377	-0.164335
O	-0.774425	-0.721098	-2.936873
H	2.730666	-1.266814	1.317252
O	1.614920	-3.153360	-0.400609
H	-1.402870	-0.923013	-2.212784
H	0.554569	-0.648376	-2.227714
O	-0.315587	-3.279070	-1.453168
H	2.057900	-2.822368	0.460852
O	2.272045	0.123544	0.514483
H	2.372039	-2.295548	2.446271
N	-0.080343	-0.679393	0.544737
H	2.936991	0.642634	0.043374
B	1.003844	0.091346	-0.198583
C	0.314213	-2.904767	-0.477989
C	-0.313219	-2.120377	0.680904
H	0.122616	-2.517598	1.607771
C	-1.834754	-2.363483	0.731379
H	-2.177148	-2.339684	1.768317
H	-2.116084	-3.316100	0.282966
C	-1.314703	0.074833	0.635001
H	-1.658649	0.200373	1.670656
C	-1.062533	1.395114	-0.031911
C	-1.965795	2.448972	-0.161100
H	-2.978288	2.371388	0.228254
C	-1.541692	3.610977	-0.807934
H	-2.229351	4.442909	-0.928162
C	-0.236114	3.709517	-1.302086
H	0.080625	4.620274	-1.802615
C	0.657580	2.645994	-1.152677
H	1.671362	2.736956	-1.538443
C	0.247777	1.473553	-0.512370
O	1.384183	-0.519011	-1.632261
H	1.905498	-1.340147	-1.598625
H	-0.777790	-1.509724	-3.499889

TS_{HI}

O	2.885644	-2.296161	1.786937
S	-2.581636	-1.028201	-0.155880
O	-1.108457	-0.739487	-3.090620
H	2.808745	-1.352440	1.536678
O	1.682003	-3.101649	-0.341789
H	-1.569134	-1.011504	-2.273025
H	0.489812	-0.601358	-2.405855
O	-0.225150	-3.159283	-1.442986
H	2.097620	-2.822142	0.540363
O	2.317551	-0.015681	0.413448
H	2.373852	-2.400875	2.601089
N	-0.076350	-0.651358	0.592715
H	2.921634	0.610187	-0.007135
B	0.993137	0.155199	-0.006933
C	0.388902	-2.835340	-0.440495
C	-0.262128	-2.097090	0.734541
H	0.183485	-2.476768	1.661989
C	-1.775914	-2.375637	0.773108
H	-2.130554	-2.346347	1.805450

H	-2.027685	-3.340199	0.332665
C	-1.336700	0.079991	0.622650
H	-1.686420	0.231117	1.652264
C	-1.071393	1.395200	-0.051710
C	-1.986296	2.420284	-0.281798
H	-3.031598	2.315048	-0.001434
C	-1.527472	3.591527	-0.886987
H	-2.222386	4.401824	-1.086617
C	-0.178718	3.732352	-1.233661
H	0.161470	4.653285	-1.698354
C	0.727406	2.699594	-0.983874
H	1.774536	2.819364	-1.254071
C	0.281475	1.517025	-0.390250
O	1.335528	-0.460560	-1.917311
H	1.832685	-1.290586	-1.966525
H	-1.071895	-1.539333	-3.633883

I

O	2.834930	-2.192607	1.873995
S	-2.625444	-1.044856	-0.082710
O	-0.821163	-0.372335	-2.887155
H	2.789049	-1.296329	1.495483
O	1.625520	-3.237590	-0.172721
H	-1.228153	-0.746774	-2.085514
H	0.978195	-0.869471	-2.842878
O	-0.185804	-3.034037	-1.413304
H	2.028321	-2.916119	0.693436
O	2.175162	-0.256600	-0.060225
H	2.333258	-2.158540	2.700619
N	-0.148120	-0.650513	0.669172
H	2.739341	0.427288	-0.448813
B	0.873893	0.159601	0.084517
C	0.382850	-2.823639	-0.356446
C	-0.297687	-2.098785	0.813320
H	0.141796	-2.455816	1.750588
C	-1.809843	-2.401432	0.826106
H	-2.174663	-2.403692	1.855123
H	-2.043488	-3.358331	0.359404
C	-1.417501	0.073362	0.721762
H	-1.749752	0.212969	1.757584
C	-1.158838	1.390024	0.044915
C	-2.074728	2.410228	-0.192482
H	-3.116447	2.312564	0.102093
C	-1.620620	3.565271	-0.832480
H	-2.317959	4.370911	-1.041876
C	-0.278233	3.698115	-1.206027
H	0.051921	4.606505	-1.700963
C	0.633718	2.674245	-0.945112
H	1.676379	2.784448	-1.233352
C	0.191583	1.508992	-0.315828
O	1.917678	-1.130044	-2.906513
H	2.218163	-1.167887	-1.987142
H	-1.146715	-0.930626	-3.607371

I’

O	2.842358	-2.839328	-1.375222
S	-2.665046	-1.234479	-0.522506
O	0.361552	-0.354677	-2.468556
H	3.072780	-1.883934	-1.363014
O	0.439896	-2.516553	-0.587043
H	-0.122033	0.470461	-2.315467
H	2.034284	-0.050556	-1.787021
O	1.027102	-3.536848	1.316685
H	1.373204	-2.786487	-0.906594
O	1.651865	-0.343917	1.138601
H	3.378027	-3.255588	-0.685165
N	-0.781078	-0.626676	1.192171
H	2.326461	0.074889	0.577407
B	0.396682	0.120043	0.861409
C	0.294751	-2.778725	0.699693
C	-0.888451	-2.071202	1.349385
H	-0.890510	-2.370762	2.400557
C	-2.223925	-2.514316	0.700235
H	-2.999644	-2.566756	1.466540

H	-2.146309	-3.486612	0.209850
C	-1.974230	0.008737	0.644077
H	-2.731754	0.196413	1.413224
C	-1.489542	1.281913	0.004477
C	-2.263593	2.248266	-0.631811
H	-3.342069	2.140046	-0.714351
C	-1.615069	3.359447	-1.174068
H	-2.193556	4.122533	-1.686114
C	-0.226908	3.504095	-1.064245
H	0.254974	4.378363	-1.491517
C	0.535881	2.536027	-0.409556
H	1.613417	2.655419	-0.324937
C	-0.097496	1.413828	0.129568
O	2.915138	-0.070069	-1.352650
H	3.514044	0.407757	-1.941441
H	0.022397	-0.974750	-1.798402

$TS_{I'J}$

O	2.957169	-2.063252	-0.840528
S	-3.394156	-0.836820	0.646524
O	-0.082193	-0.695079	-2.469070
H	2.962366	-1.363906	-1.552698
O	0.896083	-1.585923	0.367622
H	-0.284189	-0.275617	-1.616723
H	1.665011	-0.271244	-2.722150
O	0.416601	-3.747460	0.756615
H	1.831324	-1.851230	-0.126296
O	1.383546	0.081430	2.283801
H	3.750198	-1.925954	-0.302017
N	-0.971929	-0.601196	1.778829
H	2.101023	0.649463	1.973234
B	0.338113	0.020008	1.374800
C	0.146559	-2.561308	0.842241
C	-1.141384	-2.013033	1.425404
H	-1.440250	-2.633282	2.275156
C	-2.184773	-2.136843	0.293853
H	-2.671262	-3.113003	0.296328
H	-1.726892	-1.961743	-0.684611
C	-2.050202	0.255251	1.236463
H	-2.500022	0.863505	2.027972
C	-1.406319	1.164966	0.207372
C	-2.052068	2.021584	-0.681922
H	-3.137280	2.062118	-0.733432
C	-1.272564	2.818935	-1.521765
H	-1.754293	3.479596	-2.236609
C	0.125401	2.779063	-1.451682
H	0.715029	3.409297	-2.110926
C	0.760615	1.934686	-0.540320
H	1.846888	1.908809	-0.488033
C	-0.008018	1.109327	0.286767
O	2.641270	-0.178303	-2.675499
H	2.973188	-0.421573	-3.550011
H	-0.046988	-1.641266	-2.265990

J

O	3.340090	-0.986315	-0.517392
S	-3.480398	-0.683464	0.786296
O	0.015436	-0.953098	-2.488097
H	2.898535	-0.737354	-1.812322
O	0.918466	-1.610499	0.646901
H	-0.218093	-0.470147	-1.674997
H	1.437331	-0.757971	-2.693519
O	0.178108	-3.652527	0.107992
H	2.548600	-1.316688	-0.039206
O	1.312838	0.037854	2.387844
H	3.593866	-0.161490	-0.075679
N	-1.015273	-0.759770	1.821851
H	2.006152	0.622707	2.057902
B	0.389480	-0.315776	1.328580
C	0.006286	-2.551029	0.617674
C	-1.290180	-2.084439	1.257325
H	-1.586976	-2.817526	2.016318
C	-2.388892	-2.000768	0.183368
H	-2.943190	-2.935152	0.088573
H	-1.980857	-1.723820	-0.793120
C	-2.003843	0.235427	1.370745
H	-2.355707	0.860106	2.198526
C	-1.326067	1.093908	0.316030
C	-1.933805	2.055281	-0.491449
H	-3.004229	2.238752	-0.431780
C	-1.144984	2.771210	-1.393373
H	-1.602006	3.513765	-2.041151
C	0.232654	2.537112	-1.469453
H	0.835758	3.101288	-2.175452
C	0.830182	1.582785	-0.642659
H	1.903260	1.409791	-0.712052
C	0.051247	0.841164	0.255072
O	2.466144	-0.580492	-2.779491
H	2.847763	-1.212728	-3.410350
H	-0.170095	-1.886091	-2.298256

K

O	-0.572085	0.451497	3.803303
S	-2.923705	-0.981704	-1.276497
O	0.220618	-2.143632	2.978382
O	1.176550	-3.137189	0.023590
O	0.888227	1.040201	1.521464
N	-1.013704	-0.369287	0.482842
B	0.504032	0.318049	0.352932
C	0.658859	-2.036763	0.136082
C	-0.840180	-1.806471	0.116780
C	-1.438669	-2.026927	-1.275524
C	-1.944591	0.331731	-0.467923
C	-1.047467	1.118733	-1.397184
C	-1.489227	1.791990	-2.535960
C	-0.559679	2.499519	-3.295469
C	0.788368	2.526755	-2.919343
C	1.213199	1.849664	-1.776857
C	0.292825	1.134594	-1.002033
O	1.340740	-0.923513	0.254695
H	-0.016049	0.796154	3.075023
H	-0.183735	0.793283	4.618941
H	0.017633	-1.270951	3.361697
H	0.849734	1.993695	1.374789
H	-1.370351	-0.305140	1.440247
H	-1.324585	-2.444995	0.854937
H	-1.712819	-3.071419	-1.423903
H	-0.752881	-1.711421	-2.067188
H	-2.622510	0.973414	0.098111
H	-2.535400	1.765160	-2.829690
H	-0.882734	3.031001	-4.185592
H	1.503282	3.078568	-3.522596
H	2.262456	1.876574	-1.492401
H	1.188848	-2.190677	2.882417
O	2.946124	-2.630974	2.276717
H	2.648183	-2.971854	1.416845
H	3.301395	-3.397464	2.745468

4. NMR spectra

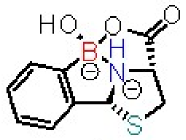

1

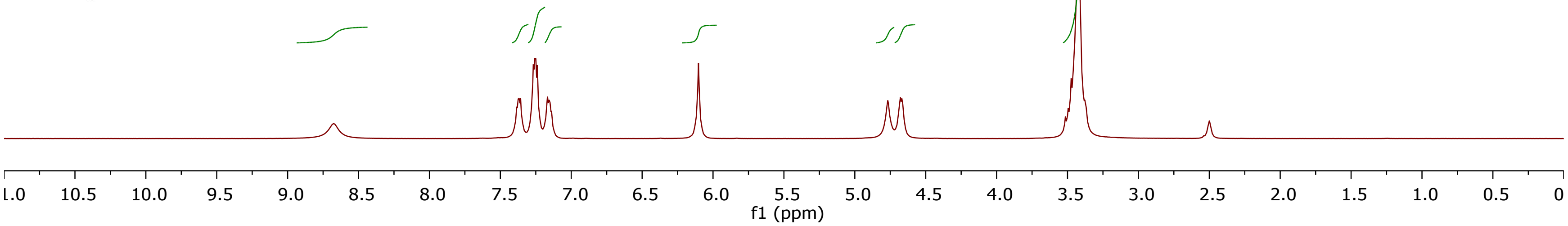

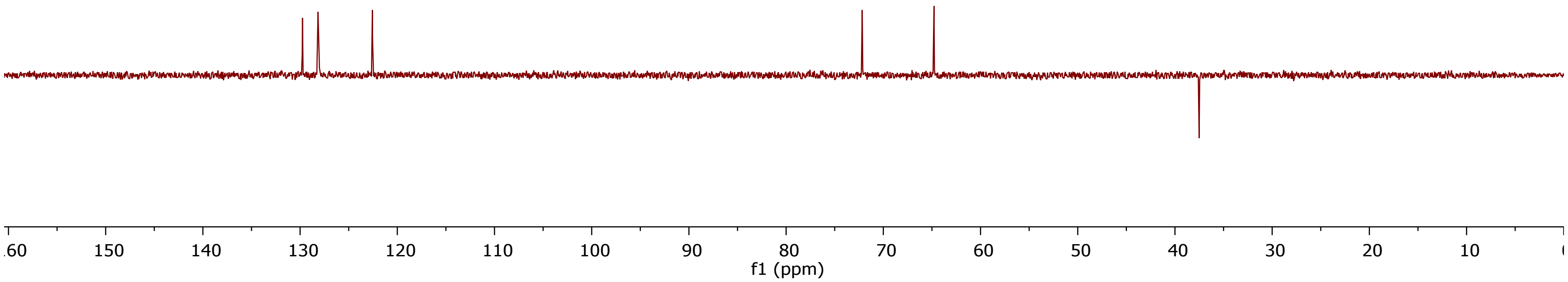

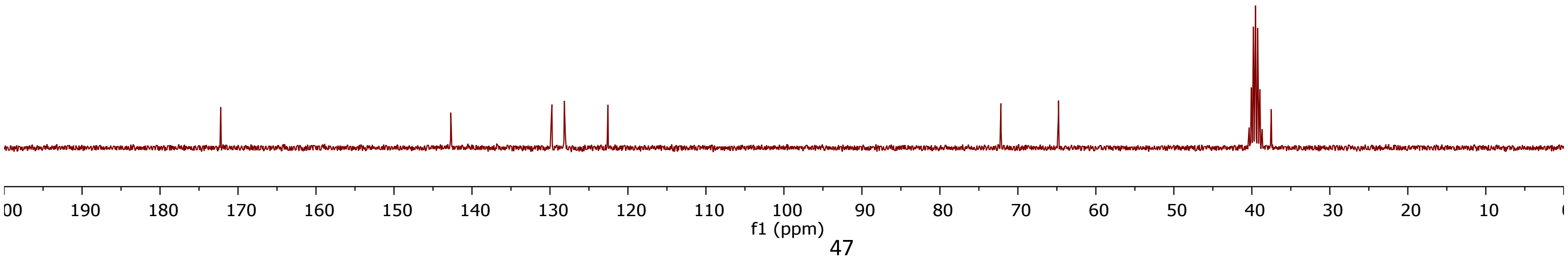

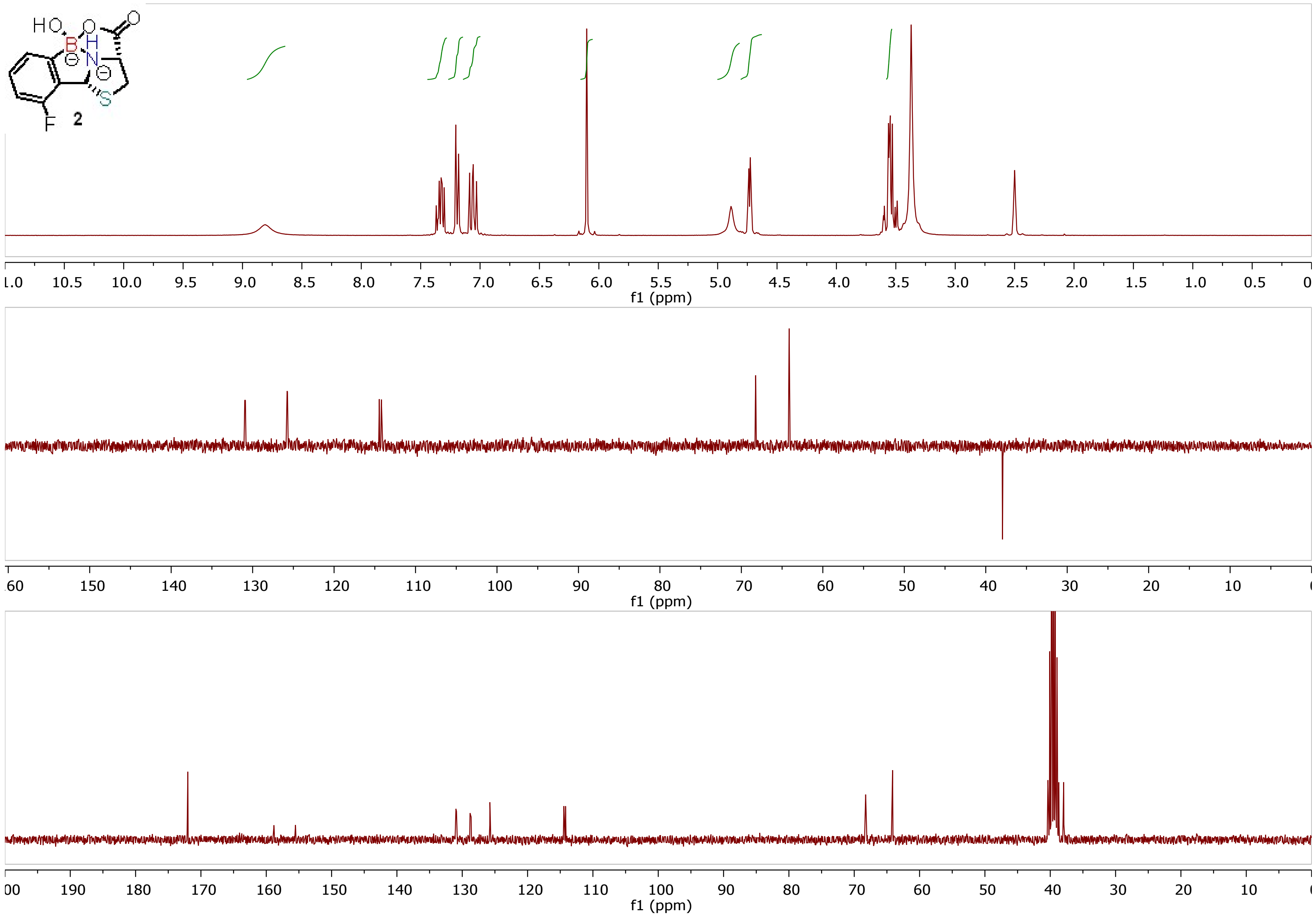
HO
O
O
B
H
N
S
F
2
f1 (ppm)
f1 (ppm)
f1 (ppm)

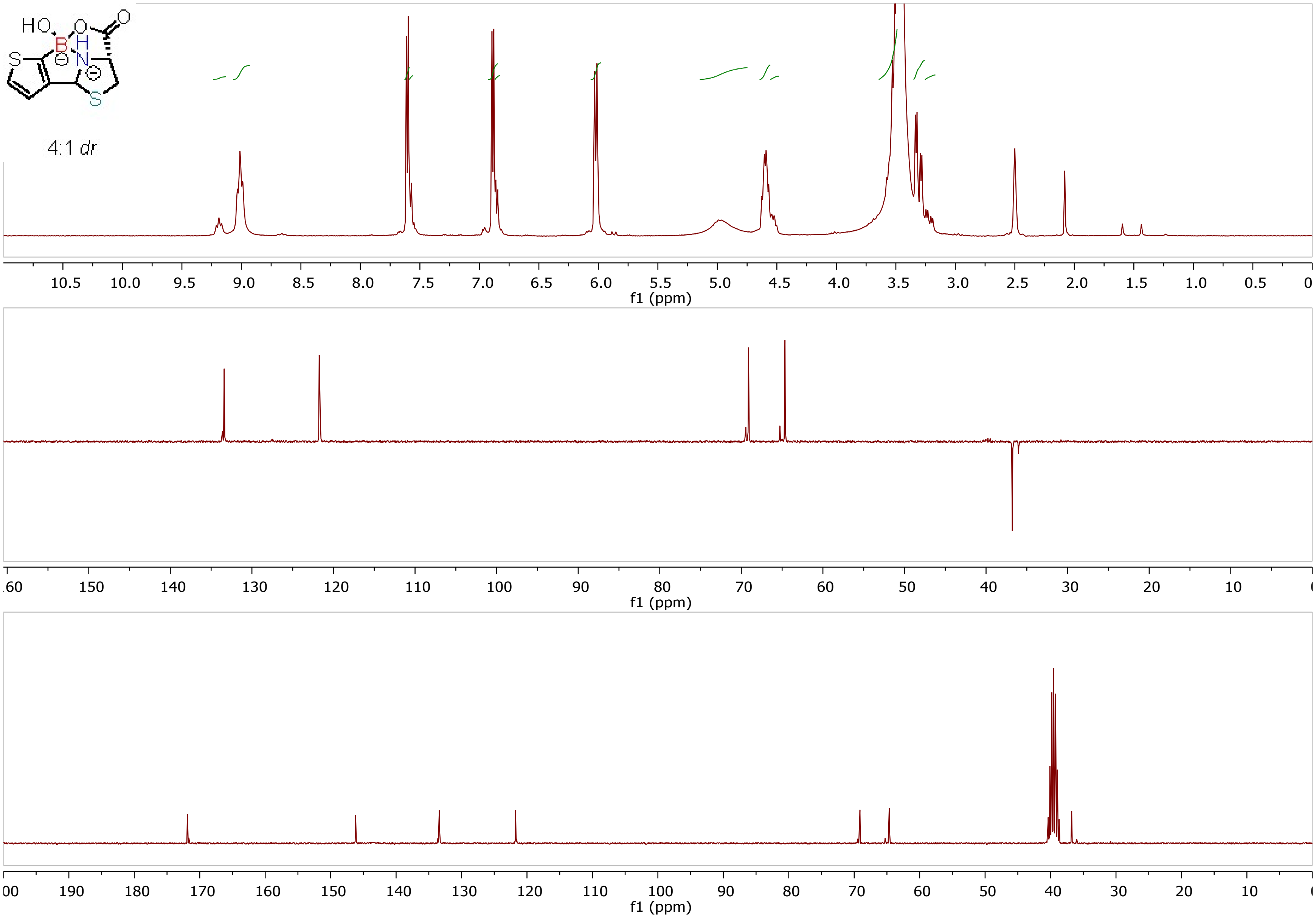
HO
O
O
B
H
N
S
S
4:1 dr
f1 (ppm)
f1 (ppm)
f1 (ppm)

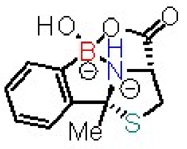
HO
O
O
B
H
N
Me
S

4

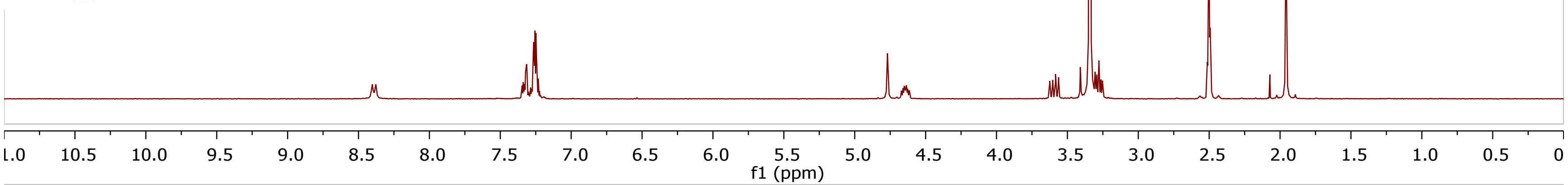
10.5 10.0 9.5 9.0 8.5 8.0 7.5 7.0 6.5 6.0 5.5 5.0 4.5 4.0 3.5 3.0 2.5 2.0 1.5 1.0 0.5 0
f1 (ppm)

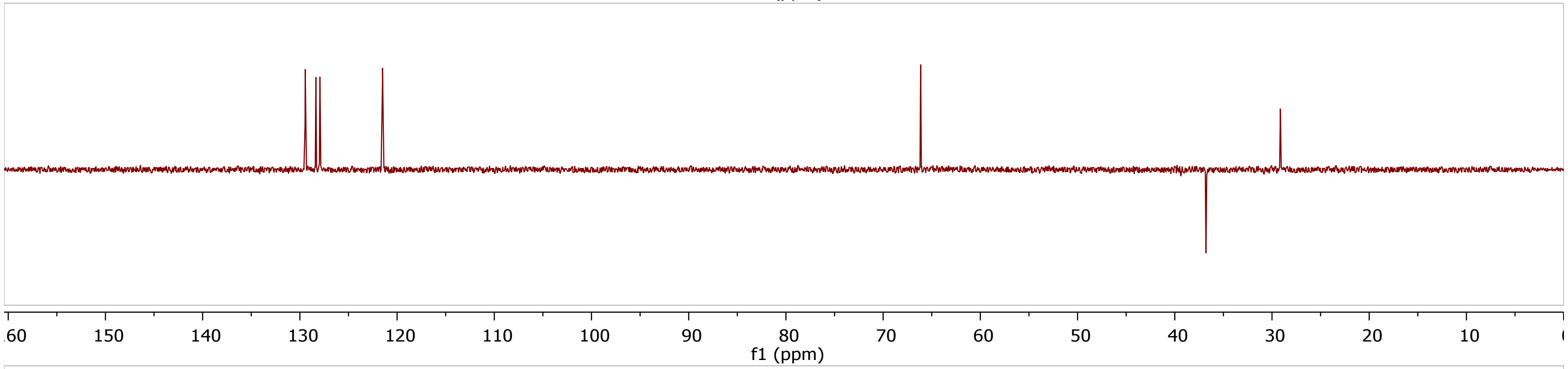
150 140 130 120 110 100 90 80 70 60 50 40 30 20 10
f1 (ppm)

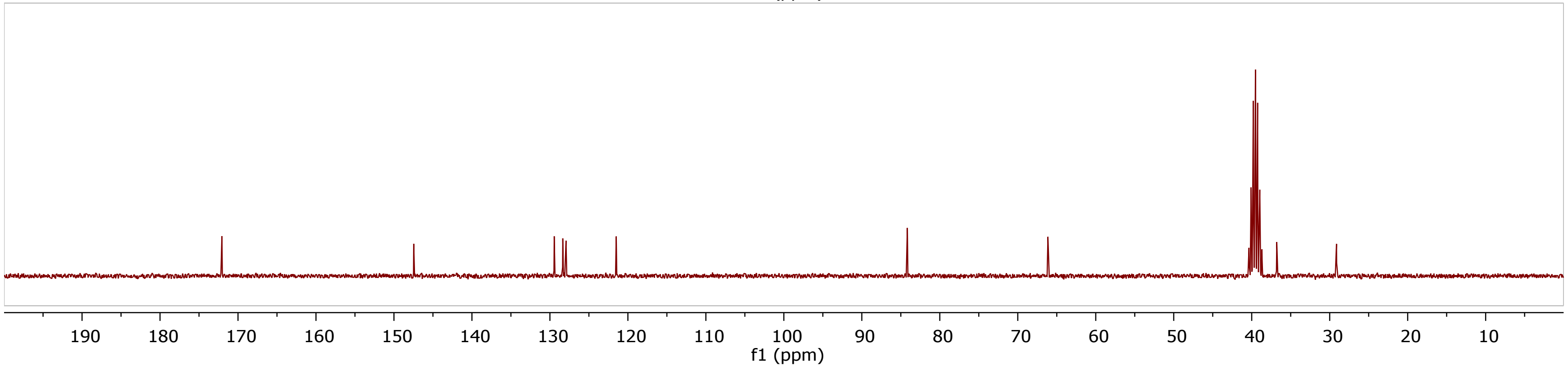
190 180 170 160 150 140 130 120 110 100 90 80 70 60 50 40 30 20 10
f1 (ppm)

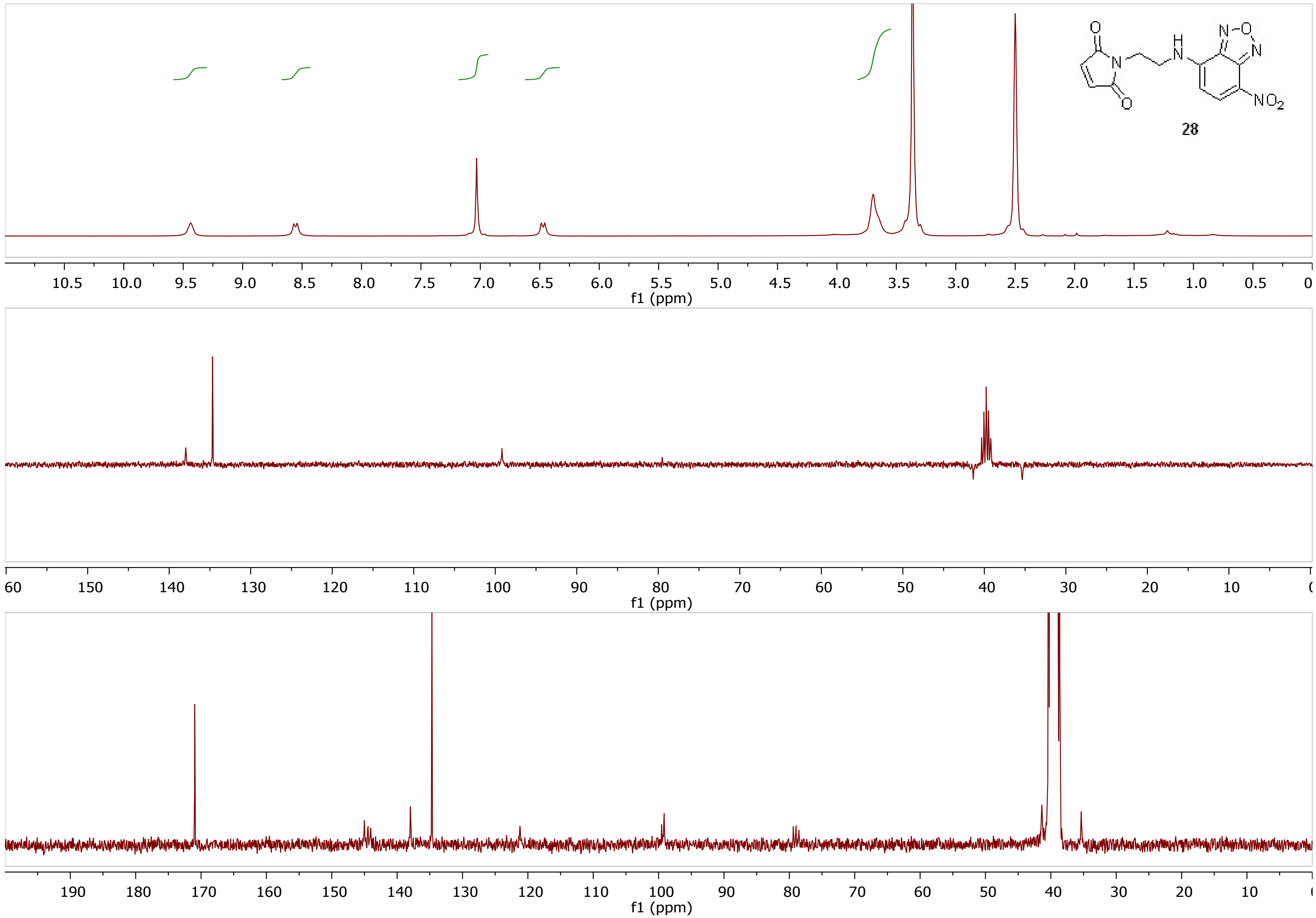
28
NO2
f1 (ppm)
f1 (ppm)
f1 (ppm)
